# Phase I Metabolism of Novel Phencyclidine Derivative 3‐Cl‐PCP: *In Vitro* Studies With Pooled Human Liver Microsomes and Investigation of a Post‐Mortem Case

**DOI:** 10.1002/dta.70097

**Published:** 2026-05-24

**Authors:** Johannes Kutzler, Tobias Veit, Thomas Keller, Volker Auwärter

**Affiliations:** ^1^ Institute of Forensic Medicine, Forensic Toxicology Medical Center – University of Freiburg Freiburg im Breisgau Germany; ^2^ Medical Faculty University of Freiburg Freiburg im Breisgau Germany; ^3^ Hermann Staudinger Graduate School University of Freiburg Freiburg im Breisgau Germany; ^4^ Institute of Forensic Medicine, Forensic Toxicology University of Salzburg Salzburg Austria

**Keywords:** dissociative anesthetics, high‐resolution mass spectrometry, human metabolism, new designer drugs, postmortem toxicology

## Abstract

Assessing fatalities linked to new psychoactive substances (NPS) often remains challenging. This study investigates a fatal intoxication involving the novel phencyclidine derivative 3‐chloro‐phencyclidine (3‐Cl‐PCP), analyzing its phase I metabolism aided by pooled human liver microsomes (pHLMs) and supported by in silico models. Postmortem samples (bile, urine, cardiac and femoral blood, and gastric contents) and three syringes were collected from a fatal intoxication case. Quantification was performed using liquid chromatography–tandem mass spectrometry (LC–MS/MS). In vitro metabolism studies employed pHLM incubation, with metabolites tentatively identified by liquid chromatography‐quadrupole time‐of‐flight mass spectrometry (LC‐QTOF‐MS). To evaluate phase II metabolism, urine and bile samples were measured with and without β‐glucuronidase treatment. 3‐Cl‐PCP concentrations ranged from 610 ng/mL in cardiac blood to 830 ng/mL in femoral blood. Higher levels were detected in urine (1200 ng/mL) and bile (4300 ng/mL). Seven phase I metabolites were tentatively identified in vitro and postmortem, primarily involving hydroxylation of the cyclohexyl (M1–M3) and piperidine (M4–M5) rings. Additionally, piperidine ring‐opened carboxyl (M6) and ring‐opened alcohol (M7) metabolites were tentatively identified. Post‐enzyme urine analysis revealed extensive glucuronidation of metabolites M1–M5, with M4 (hydroxyl piperidine) showing the highest abundance. Bile showed elevated abundance of unconjugated metabolites, particularly M6. Metabolites M4–M7 were detected in all investigated matrices. Unchanged 3‐Cl‐PCP predominantly accumulated in bile. Urine analysis should prioritize the parent compound along with the metabolites hydroxy cyclohexyl (M3), hydroxy piperidine (M4), and piperidine ring‐opened carboxyl (M6) and should include a prior β‐glucuronidase cleavage step.

## Graphical abstract

A fatal 3‐chloro‐phencyclidine (3‐Cl‐PCP) intoxication was investigated by analyzing postmortem samples and a pooled human liver microsomes assay. Tentative metabolite identification was performed by liquid chromatography‐quadrupole time‐of‐flight mass spectrometry (LC‐QTOF‐MS). Seven phase I metabolites were identified. Biotransformation included hydroxy cyclohexyl (M3), hydroxy piperidine (M4), and piperidine ring‐opened carboxyl (M6).

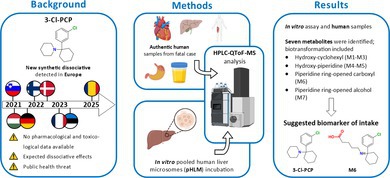

## 1 Introduction

Over the last decades, the number and diversity of new psychoactive substances (NPS) emerging on the illicit drug market have increased substantially. The European Drug Agency (EUDA) is currently monitoring about 1000 NPS that have emerged over the last 20 years, with 47 of them being reported in Europe for the first time in 2025. Since 2014, more than 300 previously identified NPS continue to be detected annually across the European Union, reflecting the diversity of the designer drug market [1]. Globally, this trend is reflected by the increase in the number of different NPS reported each year, rising from 162 in 2010 to 527 in 2023 [2]. These substances are generally classified based on their molecular structure or their pharmacological effects, with major subclasses including synthetic cannabinoids, designer stimulants, designer opioids, and novel dissociatives.

Phencyclidine (PCP) was originally developed in 1956 as a dissociative anesthetic and became a popular street drug during the 1960s [3]. However, its severe adverse effects led to its withdrawal from clinical use, and it was replaced by the equipotent ketamine, which has a more benign side effect profile [4]. Although the exact mechanisms of PCP's side effects are unknown, the formation of reactive metabolites and their irreversible binding to proteins in the central nervous system have been hypothesized to play a major role [5]. Despite these risks, PCP‐based dissociative anesthetics continued to appear on the illicit drug market. “Dissociative anesthetics” were originally defined as agents producing a distinctive form of anesthesia characterized by analgesia, amnesia, and catalepsy, while minimally affecting respiratory function. Today, however, the term “dissociative drugs” has broadened to encompass not only anesthetic compounds but also hallucinogenic substances that induce “dissociative” states, including sensory distortions, hallucinations, and dream‐like or trance‐like experiences, often combined with a feeling of alienation from the body [3]. From a molecular structure perspective, many of these substances belong to the class of arylcyclohexylamines. Between the late 1960s and the 1990s, more than 14 arylcyclohexylamine derivatives were synthesized for nonmedical use [3]. With the rise of online drug availability and so‐called “legal highs,” a new wave of arylcyclohexylamine analogs appeared on the drug market. With the emergence of methoxetamine in 2010 [6], at least a dozen additional dissociative drugs have since appeared that were not previously reported in the scientific literature. In recent years, the group of dissociatives has become more diverse. The recent emergence of uncontrolled PCP or ketamine alternatives includes 3‐Cl‐PCP (1‐[1‐(3‐chlorophenyl)‐cyclohexyl]‐piperidine) [7], tilmetamine (2‐(methylamino)‐2‐(thiophen‐2‐yl)‐cyclohexan‐1‐one) [8], 3‐Me‐PCE (*N*‐ethyl‐1‐(3‐methylphenyl)‐cyclohexan‐1‐amine) [1, 9], fluorexetamine (2‐(ethylamino)‐2‐(3‐fluorophenyl)‐cyclohexan‐1‐one) [10], 2‐fluoro‐deschloro‐*N‐*ethylketamine (2‐FDCNEK, 2‐(ethylamino)‐2‐(2‐fluorophenyl)‐cyclohexanone) [10, 11, 12], *N*‐propyl ephenidine (*N*‐(1,2‐diphenylethyl)propan‐1‐amine) [13], and hydroxetamine (2‐(ethylamino)‐2‐(3‐hydroxyphenyl)‐cyclohexanone) [14, 15, 16].

The NPS market is characterized by a continuously high rate of newly emerging compounds. Among them, arylcyclohexylamines represent one subclass exhibiting dissociative effects primarily through *N*‐methyl‐d‐aspartate (NMDA) receptor antagonism [4]. The structural diversity of arylcyclohexylamines facilitates the continuous emergence of new analogs designed to circumvent existing drug regulations while retaining or modifying their psychoactive properties [17, 18]. These substances frequently pose substantial analytical challenges due to their structural diversity, rapid evolution, lack of reference material standards, and analytical data for characterization [1]. The metabolism of these substances often involves cytochrome P450 isoenzymes, resulting in the formation of a large number of phase I metabolites, which can be subject to phase II conjugation. This metabolic complexity further complicates detection and characterization in biological samples. Consequently, both in vivo and in vitro studies are essential for identifying these metabolic pathways. The application of experimental models, such as pooled human liver microsomes (pHLMs), has proven particularly valuable, as this system circumvents ethical issues while offering an accessible, cost‐effective, and reliable tool for investigating NPS metabolism [19].

Consequently, recent studies have used human liver microsomes as a tool for metabolism elucidation [20, 21, 22, 23, 24, 25]. PCP undergoes bioactivation via piperidine ring oxidation to a reactive iminium ion (equilibrium with enamine and carbinolamine intermediates), which might contribute to its hepatotoxicity and P450 inactivation [5]. Further CYP‐mediated oxidation yields a dihydropyridinium ion that could form irreversible adducts with proteins. Additional pathways include epoxidation via an enamine intermediate, aliphatic hydroxylation, and aromatic hydroxylation, leading to quinone methide formation. Investigations into the metabolic pathways of PCP derivatives, such as the study by Michely et al. [26] on 3‐methoxy phencyclidine (3‐MeO‐PCP) and 3‐methoxy rolicyclidine (3‐MeO‐PCPy), have demonstrated the involvement of cytochrome P450 enzymes, including CYP2B6, CYP2C19, and CYP2D6. The biotransformations involve *O*‐demethylation, aliphatic hydroxylation at the cyclohexyl and heterocyclic rings, carboxylation after ring opening, and glucuronidation [26, 27]. Studies by Davidsen et al. on 3‐HO‐PCP have identified several key metabolites using pooled human hepatocytes and in vivo analyses of human samples [28]. Phase I metabolism involves the formation of a piperidine mono‐hydroxylated metabolite and an *N*‐dealkyl carboxylic acid metabolite. Phase II metabolism includes *O*‐glucuronidated and *O*‐sulfate‐conjugated metabolites. The parent compound, 3‐HO‐PCP, along with the *O*‐glucuronidated metabolite, was identified as a key analytical target in blood and brain samples, with the parent 3‐HO‐PCP showing the highest signal intensity.

Such detailed metabolic insights are essential for forensic toxicology to develop targeted screening methods for NPS and their active metabolites. The dynamic NPS market requires ongoing research, as emerging compounds challenge detection in biological samples. This study aims to investigate the metabolism of the novel PCP derivative 3‐chloro‐phencyclidine (3‐Cl‐PCP) in a fatal intoxication case. We compare the phase I and II metabolites detected in postmortem biological matrices with phase I metabolites generated with pHLMs and predicted by in silico models. The goal is to identify stable and unambiguous metabolites suitable for detecting 3‐Cl‐PCP consumption over extended time periods when the parent compound might no longer be detectable.

## 2 Materials and Methods

### 2.1 Samples

A fatal intoxication with the substance 3‐Cl‐PCP was examined. The available specimens included bile, urine, and cardiac and femoral blood, as well as gastric contents and three syringes.

### 2.2 Chemicals and Reagents

#### 2.2.1 Reagents for the pHLM Assay

Phosphate buffer (0.5 M, pH 7.4) was obtained from Corning (Manassas, VA, USA). 3‐Chloro‐phencyclidine hydrochloride (3‐Cl‐PCP HCl) was purchased from Cayman Chemical (Ann Arbor, MI, USA). NADPH System solution A (glucose‐6‐phosphate, NADH) and solution B (glucose‐6‐phosphate‐dehydrogenase) were acquired from Discovery Labware (Woburn, MA, USA). Xtreme 200 Human Liver Microsomes were sourced from Xenotech (Kansas City, KS, USA). Acetonitrile was supplied by VWR International (Rosny‐sous‐Bois‐cedex, France). A 10‐M solution of ammonium formate was obtained from Sigma‐Aldrich Chemie GmbH (Steinheim, Germany). β‐Glucuronidase (*Escherichia coli* K12) was purchased from Roche Diagnostics GmbH (Mannheim, Germany).

#### 2.2.2 Reagents for Solutions and Eluents

Concentrated formic acid was obtained from Carl Roth GmbH + Co. KG (Karlsruhe, Germany), and concentrated acetic acid was obtained from Sigma‐Aldrich Chemie GmbH (Steinheim, Germany). Potassium dihydrogen phosphate, potassium hydroxide, dichloromethane, and isopropanol were purchased from Carl Roth GmbH + Co. KG (Karlsruhe, Germany). Ammonia was sourced from Merck KGaA (Darmstadt, Germany). Methanol (LC–MS Grade) was acquired from Honeywell (Seelze, Germany). Hydrochloric acid 37% was supplied by VWR International (Rosny‐sous‐Bois‐cedex, France). The internal standard phencyclidine‐d_5_ (PCP‐d_5_) was purchased from Cayman Chemical (Ann Arbor, MI, USA) and diluted to 0.5 μg/mL.

The LC‐QTOF/MS analysis employed two mobile phases. Mobile phase Bruker Ce consisted of aqueous 0.1% formic acid and 25 mM of ammonium formate. Mobile phase Bruker De was methanolic 0.1% formic acid and 25 mM of ammonium formate. For LC–MS/MS analysis, mobile phase Bruker A consisted of water/acetonitrile (98:2, v/v) containing 2 mM of ammonium formate and 0.1% formic acid. Mobile phase Bruker B was composed of acetonitrile containing 2 mM of ammonium formate and 0.1% formic acid. All eluents were freshly prepared prior to analysis. The sodium formate/acetate clusters solution used for external and internal mass calibration of the QTOF/MS instrument was prepared by mixing 250 mL of deionized water, 250 mL of isopropanol, 750 μL of acetic acid, 250 μL of formic acid, and 500 μL of sodium hydroxide 1 M.

### 2.3 pHLM Preparation

The in vitro metabolism study using pHLM was conducted, adapting a previously described method [29].

The 100‐μL reaction mixture consisted of 5 μL of pHLM solution, 1 μL of 3‐Cl‐PCP solution (1 mg/mL in methanol), NADPH regenerating components (5 μL of solution A and 1 μL of solution B), 20 μL of phosphate buffer, and 68 μL of deionized water. No UDPG‐generating system was added, so the extent of glucuronidation or other forms of conjugation could only be studied in vivo. Incubation occurred for 60 min at 37°C with constant agitation at 500 rpm. The reaction was terminated by adding 100 μL of ice‐cold acetonitrile. To enhance phase separation, 50 μL of concentrated ammonium formate solution (10 M) was added. After centrifugation, the upper organic phase was carefully extracted and stored at −20°C. All experiments were performed in triplicate. Two negative controls were prepared: one replacing pHLM with water to identify nonmetabolically generated compounds and the other substituting water for the substrate to exclude interfering substances. For LC‐QTOF/MS analysis, 30 μL of supernatant was evaporated under a gentle nitrogen stream and reconstituted in 50 μL of a 1:1 mixture of mobile phases Bruker A and B. The same procedure was applied for LC–MS/MS analysis, using 5 μL of supernatant. Finally, the resulting metabolites were ranked in descending order based on their chromatographic peak areas.

### 2.4 In Silico Metabolite Generation

To identify potential metabolites, two distinct computational models were employed: GLORYx [30] and EAWAG‐BBD [31]. GLORYx integrates machine learning–based site of metabolism prediction with reaction rule sets to predict and rank the structures of metabolites formed by both human phase I and phase II metabolism. It calculates a probability score ranging from 0 to 1, reflecting the likelihood of metabolite occurrence. In this study, only phase I metabolites were considered, as they align with the pHLM assay capabilities used for experimental validation and represent the primary metabolic transformations. The EAWAG‐BBD model was developed to predict the oxidative degradation of substances by microorganisms under aerobic conditions. It was utilized to identify additional metabolites not predicted by GLORYx, complementing the metabolite identification process. This approach of using multiple computational tools provides a more comprehensive assessment of potential metabolic pathways and transformation products.

### 2.5 Quantification of 3‐Cl‐PCP in Biological Matrices

#### 2.5.1 Sample Preparation

Quantification in biological material was performed on various matrices, including bile, urine (both with and without β‐glucuronidase hydrolysis), cardiac blood, femoral blood, and gastric contents, following a sample preparation protocol established previously [32, 33]. The preparation method varied depending on whether the specimens were subjected to β‐glucuronidase hydrolysis.

For specimens not treated with β‐glucuronidase, a standardized protocol was followed. Each sample was prepared by combining 60 μL of liquid specimen with 2940 μL of phosphate buffer (pH 6) and 60 μL of the internal standard. This mixture was thoroughly homogenized and subsequently divided into six equal aliquots of 510 μL each. For bile fluid and urine samples processed with β‐glucuronidase cleavage, a modified preparation method was employed. To prepare the bile sample solution, 100 μL of bile fluid was first diluted with 900 μL of phosphate buffer, and 100 μL of this diluted solution was used for β‐glucuronidase cleavage. In contrast, urine was used undiluted. The reaction mixture for both bile and urine consisted of 100 μL of sample solution, 100 μL of phosphate buffer, 30 μL of internal standard, and 15 μL of β‐glucuronidase. This mixture was incubated for 120 min at 45°C.

To prevent inhibition of the β‐glucuronidase cleavage by the addition of a large proportion of methanolic internal standard, a lower ratio of internal standard to sample solution was chosen (10:3 for glucuronide cleavage, compared to 1:1 for samples without β‐glucuronidase cleavage). This adjustment ensured that the enzymatic activity was not compromised by the presence of methanol from the internal standard. During processing, the samples were diluted by a factor of approximately 0.41 (100 μL of matrix yields about 245 μL of sample volume). This dilution factor was taken into account in the subsequent comparison of metabolite abundances across various matrices.

For the preparation of syringe contents, 100 μL of sample and 900 μL of deionized water were added to an Eppendorf tube. From this initial dilution, 10 μL were taken for syringes No. 1 and No. 3, whereas 5 μL were taken for syringe No. 2. Each was then filled to 1 mL with deionized water. Subsequently, this sample solution was mixed with 10 μL of internal standard and 2.5 mL of phosphate buffer, followed by automated solid‐phase extraction. Calibration was performed using a Hamilton syringe and a reference solution with a concentration of 1 μg/mL. The calibration scheme involved preparing solutions with final concentrations of 1, 2, 5, 10, 20, and 50 ng/mL by adding appropriate volumes of the reference solution to deionized water. For each sample, 500 μL of sample volume, 10 μL of internal standard, and 2.5 mL of phosphate buffer were mixed and subsequently extracted using the ASPEC solid‐phase extraction automat. All further steps were performed analogously to the processing of samples using standard addition.

#### 2.5.2 Standard Addition Preparation

The standard addition method, a technique that involves adding known and varying amounts of the target analyte to the sample, was employed to determine the original analyte concentration by extrapolation. This method was implemented using a series of six aliquots, designated as StAdd‐0 through StAdd‐5, with increasing amounts of the 3‐Cl‐PCP reference standard solution added. A Hamilton syringe was used for precise volume control during these additions.

For samples without β‐glucuronidase hydrolysis, each aliquot contained the equivalent of 10 μL of the original sample and 10 μL of the internal standard. Preliminary analyses estimated the analyte concentration in the original sample to be approximately 500 ng/mL, corresponding to 5 ng per aliquot. StAdd‐0 served as the unspiked sample, whereas the subsequent aliquots had addition factors of 0.25, 0.5, 1, 2, and 4 times the estimated sample concentration, respectively. Consequently, the masses of 3‐Cl‐PCP added to each aliquot were 0, 1.25, 2.5, 5, 10, and 20 ng, corresponding to volumes of 0, 2.5, 5, 10, 1, and 2 μL. Two different concentrations of reference solutions were used: 0.5 μg/mL for StAdd‐1 through StAdd‐3 and 10 μg/mL for StAdd‐4 and StAdd‐5.

For β‐glucuronidase–treated samples, the preparation process differed slightly. Initially, 60 μL of the prepared sample was added to 2940 μL of phosphate buffer, mixed, and divided into six aliquots of 500 μL each. The standard addition was then performed using these aliquots, with StAdd‐0 serving as the unspiked sample representing the baseline matrix. Subsequent aliquots received increasing volumes of a 1 μg/mL 3‐Cl‐PCP solution: StAdd‐1 was spiked with 2 μL of the reference solution (representing 2 ng of substance), and StAdd‐2, StAdd‐3, StAdd‐4, and StAdd‐5 were spiked with 4 μL (4 ng), 6 μL (6 ng), 8 μL (8 ng), and 10 μL (10 ng), respectively. These additions corresponded to factors of 1 + 0.4, 1 + 0.8, 1 + 1.2, 1 + 1.6, and 1 + 2, respectively, relative to the estimated concentration in the original sample. This comprehensive standard addition approach enables the construction of standard addition curves for accurate quantification of 3‐Cl‐PCP in various biological matrices while accounting for potential matrix effects and glucuronide conjugates.

#### 2.5.3 Solid‐Phase Extraction

For solid‐phase extraction, all aliquots from the above‐mentioned preparation received an additional 2.5 mL of phosphate buffer (pH 6). The extraction was performed using Chromabond Drug cartridges (volume of 3 mL, filling capacity of 200 mg of modified silica gel, pore size of 60 Å, particle size of 45 μm, and specific surface of 500 m^2^/g) supplied by Macherey‐Nagel GmbH & Co KG (Düren, Germany). These cartridges feature a bifunctional modification combining an octyl phase and a strong cationic exchanger (benzol sulfonic acid). The extraction process was automated using the ASPEC GX‐274 by Gilson Inc. (Middleton, WI, USA), following a slightly modified method described by Grapp et al. [32].

The extraction procedure started with the cartridge conditioning using 2 mL of methanol followed by 2 mL of phosphate buffer. Subsequently, 3 mL of the sample was loaded onto each cartridge. To ensure rinsing of all needles and tubes prior to extraction, 4 mL of a 50:50 methanol/water mixture was used. The cartridges were then washed in three sequential steps, using 1 mL each of deionized water, 0.1 M of acetic acid, and methanol. After drying the cartridges with nitrogen, the analytes were eluted using 1.5 mL of a mixture containing dichloromethane, isopropanol, and 25% ammonia (79:19:2, v/v/v). The eluate was then dried under a gentle stream of nitrogen at 40°C. To prevent vaporization of the volatile amines, 100 μL of a mixture containing 75 μL of isopropanol and 25 μL of hydrochloric acid was added before the extract reached complete dryness. Finally, the sample was reconstituted with 100 μL of mobile phase Bruker A/B (99:1) for LC–MS/MS analysis.

### 2.6 Analytical Methods

#### 2.6.1 Instrumentation

The LC‐QTOF/MS system consisted of an Impact II mass spectrometer manufactured by Bruker Daltonik GmbH (Bremen, Germany). The instrument was equipped with two ion sources: an Apollo ESI ion source and a VIP HESI ion source. The vacuum system employed a rotary vane pump (HS602) with a pumping speed of 29.3 m^3^/h, manufactured by Varian (Palo Alto, CA, USA). The system was operated using Compass HyStar Version 6.0 (Version 6.0.30.0) software. Additional software included Compass 2022b for otofSeries, otofControl Version 6.3 (Build 0.5; 64‐bit), and Bruker Compass DataAnalysis Version 5.3 SR1 (Build 556.396.6383; 64‐bit). The HPLC system was an Elute OLE HPG 1300 from Bruker Daltonik GmbH, with a pump, autosampler, and column oven. A separate HPLC pump (LC‐10ADVP) from Shimadzu Corporation (Duisburg, Germany) was used for calibrant delivery.

Chromatographic separation was performed using a Kinetex biphenyl column (100 × 2.1 mm, 2.6 μm of particle size, Phenomenex, Aschaffenburg, Germany) with a corresponding SecurityGuard ULTRA Cartridge UHPLC Biphenyl guard column (for 2.1 mm of internal diameter columns, Phenomenex). Additionally, orthogonal separation was achieved using the same gradient with an Intensity Solo 1.8 C_18_–2 column (100 × 2.1 mm, 1.8 μm of particle size, Bruker Daltonik GmbH, Bremen, Germany), coupled with a VanGuard BEH C_18_ 1.7 μm pre‐column (Waters, Eschborn, Germany). The mobile phases Bruker Ce and Bruker De were varied in a linear gradient program, with the column oven temperature maintained at 40°C. The autosampler was cooled to 5°C, and the injection volume was 2 μL. The gradient program was initiated with 96% mobile phase Bruker Ce at a flow rate of 0.2 mL/min, which was maintained for the first 0.1 min. From 0.1 to 1 min, the composition was changed to 81.7% Ce, still at 0.2 mL/min. At 2.5 min, the ratio was shifted to 50:50 Ce:De with a slight increase in flow rate to 0.223 mL/min. By 14 min, the composition was changed to 0.1% Ce, with the flow rate increased to 0.4 mL/min. This composition was maintained until 16 min, at which point the flow rate was increased to 0.48 mL/min. At 16.1 min, the mobile phase was rapidly returned to the initial 96:4 Ce:De ratio for column re‐equilibration. This composition and flow rate were held until 19 min, after which the flow rate was decreased back to 0.2 mL/min for the final minute of the run.

Data acquisition and processing were performed using HyStar version 3.2 and DataAnalysis version 4.2, respectively (both from Bruker Daltonik). The QTOF‐MS was operated in positive electrospray ionization (ESI+) mode, acquiring spectra in the range of *m/z* 30–650 Da at an acquisition rate of 4.0 Hz. Two acquisition modes were employed: full scan/bbCID and full scan/auto‐MS/MS with a mass inclusion list (Table S1). For both modes, the collision energy (CE) was set at 30 ± 9 eV. The following parameters were used for the MS analysis: dry gas temperature of 200°C, dry gas flow of 8 L/min, and nebulizer gas pressure of 200 kPa. Nitrogen served as the collision gas. The capillary and end plate offset voltages were set to 2500 and 500 V, respectively. External and internal mass calibrations were performed using sodium formate/acetate clusters and high‐precision calibration mode. The mass calibration was performed regularly before every batch run. Metabolites generated in vitro (Section 2.3) and in vivo (Section 2.5) were tentatively identified and characterized by LC‐QTOF/MS analysis through manual data processing. The identification criteria included a mass error of the precursor ion < 5 ppm, a signal‐to‐noise ratio > 3:1, and a mass tolerance for fragment ions of ±10 ppm.

The LC–MS/MS system consisted of a Triple Quad 6500+ tandem mass spectrometer equipped with a TurboIonSpray interface, operated in positive ESI mode, manufactured by Sciex (Darmstadt, Germany). The HPLC system was composed of various Shimadzu components: two binary HPLC pumps, a CTO‐10ASVP column oven (set to 30°C), and a SIL‐20ACXR autosampler used for sample injection. Chromatographic separation was mainly achieved using a Kinetex Biphenyl column (100 × 2.1 mm, 2.6 μm of particle size, Phenomenex, Aschaffenburg, Germany) with a corresponding SecurityGuard ULTRA Cartridge UHPLC Biphenyl guard column (for 2.1 mm of internal diameter columns, Phenomenex). The following LC gradient program was employed: Initial conditions of 5% mobile phase B were maintained for 3 min, followed by a linear increase to 15% B over 5 min, which was then held for 3 min. Subsequently, mobile phase B was increased to 80% over 7 min. For column cleaning, mobile phase B was rapidly increased to 95% within 0.5 min and maintained for 2.5 min. Finally, re‐equilibration was achieved by returning to initial conditions in 0.5 min and holding for 4.5 min. The total runtime was 26 min with a flow rate of 0.3 mL/min. Isopropanol was added by postcolumn infusion at a flow rate of 0.1 mL/min to enhance signal intensity.

Data acquisition was performed in multiple reaction monitoring (MRM) mode using Analyst software (version 1.6.3). The MRM transitions were acquired with a cycle time of 1.5 s and a dwell time between 3 and 250 ms. Mass spectrometer parameters (declustering potential [DP], entrance potential [EP], CE, and collision cell exit potential [CXP]) were optimized for 3‐Cl‐PCP to obtain the best possible signal intensities. The most abundant ion transition for the PCP‐d_5_, as well as the two most abundant ion transitions for 3‐Cl‐PCP, were included in the MRM method (3‐Cl‐PCP with RT 15.1 min, quantifier: Q1 *m/z* 278.2, Q3 *m/z* 86.2, DP 40 V, EP 10 V, CE 18 V, CXP 18 V; qualifier: Q1 *m/z* 278.2, Q3 *m/z* 193.2, DP 40 V, EP 10 V, CE 21 V, CXP 21 V; PCP‐d_5_ with RT 14.81 min: Q1 *m/z* 249.1, Q3 *m/z* 164.1, DP 50 V, EP 6 V, CE 20 V, CXP 20 V). Mass spectrometer source conditions were as follows: curtain gas pressure, 30 psi (207 kPa); collision gas pressure, 6 psi (41 kPa); ionspray voltage, 4000 V; and temperature, 350°C.

## 3 Results and Discussion

### 3.1 In Silico Metabolite Prediction

The GLORYx model predicted 18 phase I metabolites. Of these, 15 metabolites had a probability score exceeding 0.05 and are presented in Table 1. The probability score, ranging from 0 to 1, indicates the likelihood of metabolite formation, with higher scores suggesting a greater probability of occurrence. The majority of the metabolites can be classified into two main groups: those resulting from monooxygenation of the parent compound (G2–G10) and those arising from formal dehydrogenation (G12–G15). Notably, a dehydrogenated metabolite can also be formed through hydroxylation followed by dehydration. Metabolite G11 can be viewed as an open‐chain form of metabolite G2, as hemiaminals exist in tautomeric equilibrium with their corresponding carbonyl and amine forms [34, 35]. Metabolite G1 is formed through monooxygenation followed by oxidation to a lactam.

**TABLE 1:** In silico predicted metabolites by GLORYx with a probability score (PS) > 0.05. The PS, with a range of 0 to 1, indicates the likelihood of metabolite formation.

#	Metabolite structure	PS	#	Metabolite structure	PS
G1	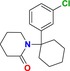	0.72	G2	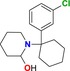	0.72
G3	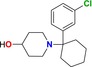	0.72	G4	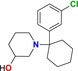	0.72
G5	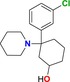	0.64	G6	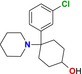	0.64
G7	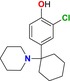	0.62	G8	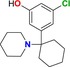	0.62
G9	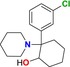	0.59	G10	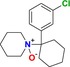	0.59
G11	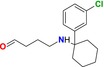	0.59	G12	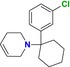	0.14
G13	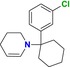	0.14	G14	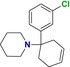	0.13
G15	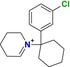	0.12			

**FIGURE 1:**
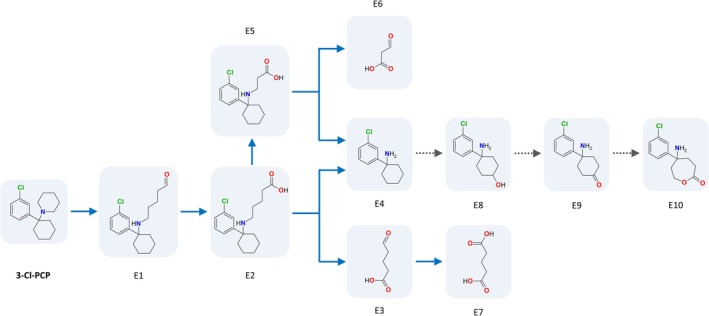
In silico metabolism predicted by EAWAG‐BBD. According to the software results, blue arrows indicate likely biotransformations, whereas gray dotted arrows represent less likely metabolite formations.

To identify additional metabolites not predicted by GLORYx, a second in silico model, EAWAG‐BBD, was employed. The structures of these metabolites and the proposed metabolic pathway are depicted in Figure 1. This model initially suggested an open‐chain aldehyde (E1, identical to G11), which can subsequently be oxidized to the corresponding ω‐carboxylic acid (E2). From this open‐chain carboxylic acid, a process analogous to β‐fatty acid oxidation can occur, resulting in a metabolite with a carbon chain shortened by two units (E5). This biotransformation has also been observed in certain synthetic cannabinoids [36, 37]. Alternatively, an *N*‐dealkylation can take place, yielding a primary amine (E4) and 5‐oxopentanoic acid (E3, glutaric acid semialdehyde), the latter of which can be further oxidized to glutaric acid (E7). E4 can also be formed from E5 through the elimination of the 3‐carbon ketone body 3‐oxopropanoic acid (E6, also 3‐oxopropionic acid). The resulting primary amine E4 can then undergo several subsequent, less likely transformations. A hydroxylation at the 4‐position of the cyclohexane ring could occur to form E8, which could then be oxidized to the corresponding cyclohexanone (E9), and this could finally be metabolized to a lactone (E10) via a Baeyer‐Villiger‐type oxidation.

### 3.2 Tentative In Vitro Metabolite Identification

#### 3.2.1 Interpretation of the High‐Resolution Mass Spectrum (HR‐MS/MS) of 3‐Cl‐PCP

**FIGURE 2:**
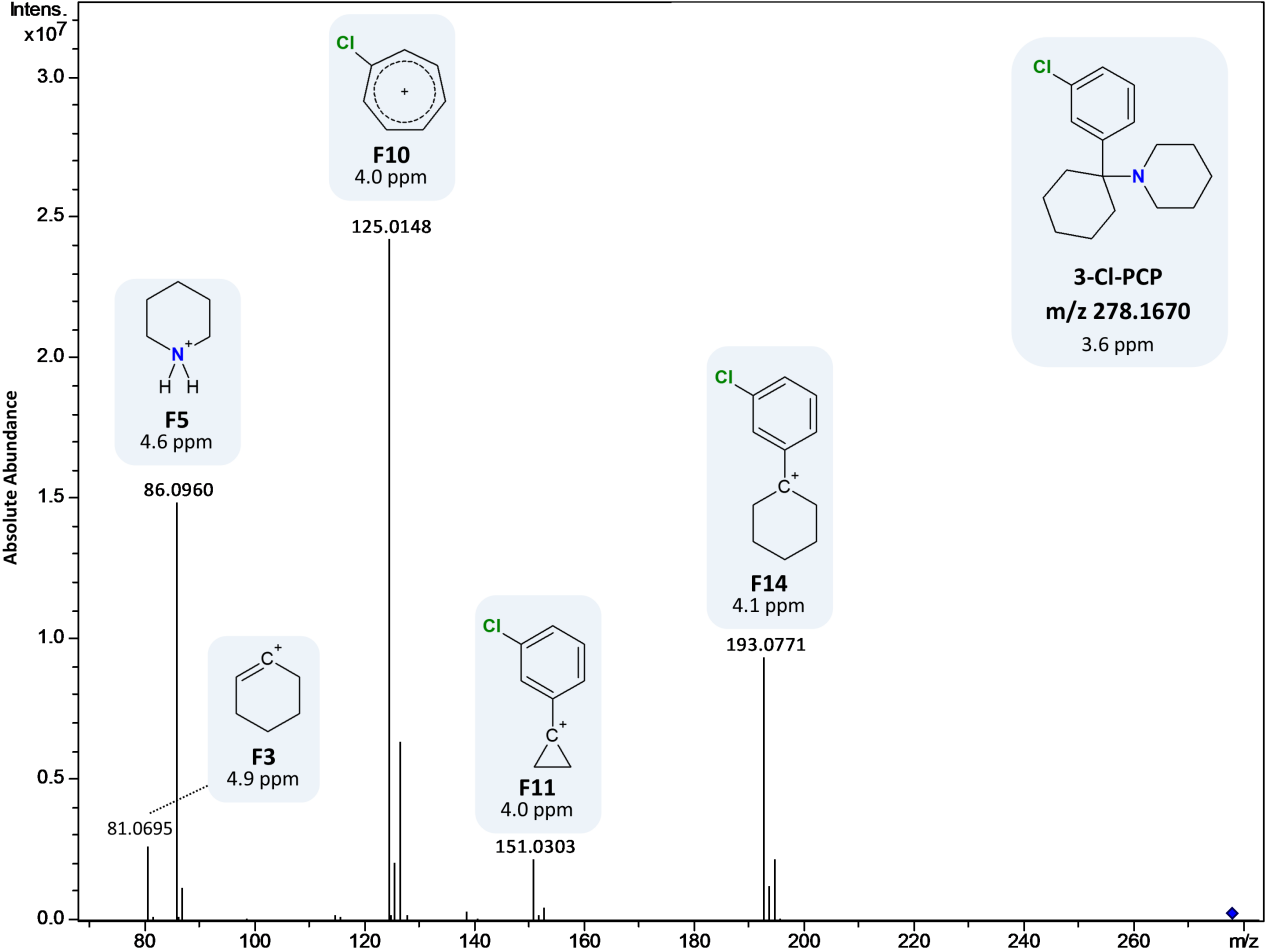
MS/MS spectrum of 3‐Cl‐PCP. Additionally, possible fragment structures are shown, including the relative mass deviation (in parts per million) from the theoretical monoisotopic mass of the proposed fragments.

To identify phase I metabolites, the pHLM approach was analyzed using LC‐QTOF/MS. It is important to note that these analyses provide tentative rather than definitive structural elucidations. The proposed metabolites are based on the fragmentation pattern of the reference standard and common metabolic reactions. A particularly characteristic feature is the isotopic pattern of fragments containing a chlorine atom. To understand the fragmentation of the underlying molecular scaffold and draw conclusions about the structure of the metabolites, the LC‐QTOF/MS spectrum of 3‐Cl‐PCP was initially examined (Figure 2).

During fragmentation reactions, the molecule can be divided into three distinct parts: the aromatic ring, the cyclohexane ring, and the piperidine ring. These fragment ions will change depending on how the identified metabolite structurally differs from its parent (a comprehensive list of fragment ions and their postulated structures is available in Table S2). This assumption is based on the premise that the fragmentation reactions of 3‐Cl‐PCP do not fundamentally change after metabolization. The primary focus is on the following fragments and their metabolically altered derivatives: **F14** (chlorophenyl cyclohexyl cation, calculated mass‐to‐charge ratio *m/z* 193.0779, difference measured to calculated mass −3.9 ppm), **F10** (chlorotropylium cation, *m/z* 125.0153, −3.6 ppm), and **F5** (piperidinium cation, *m/z* 86.0964, −4.9 ppm). Changes on these three fragments provide crucial information about the structure of the respective metabolite. The fragments containing a chlorine atom (e.g., **F14**, **F11**, and **F10**) are primarily associated with the lighter isotope ^35^Cl. Their corresponding heavier counterparts, generated by ^37^Cl, are less abundant and appear at an *m/z* value approximately 2 units higher due to the mass difference of +1.997. These peaks exhibit an intensity ratio reflecting the natural ^37^Cl/^35^Cl abundance of 32%.

#### 3.2.2 Monooxygenated Metabolites M1–M5

**FIGURE 3:**
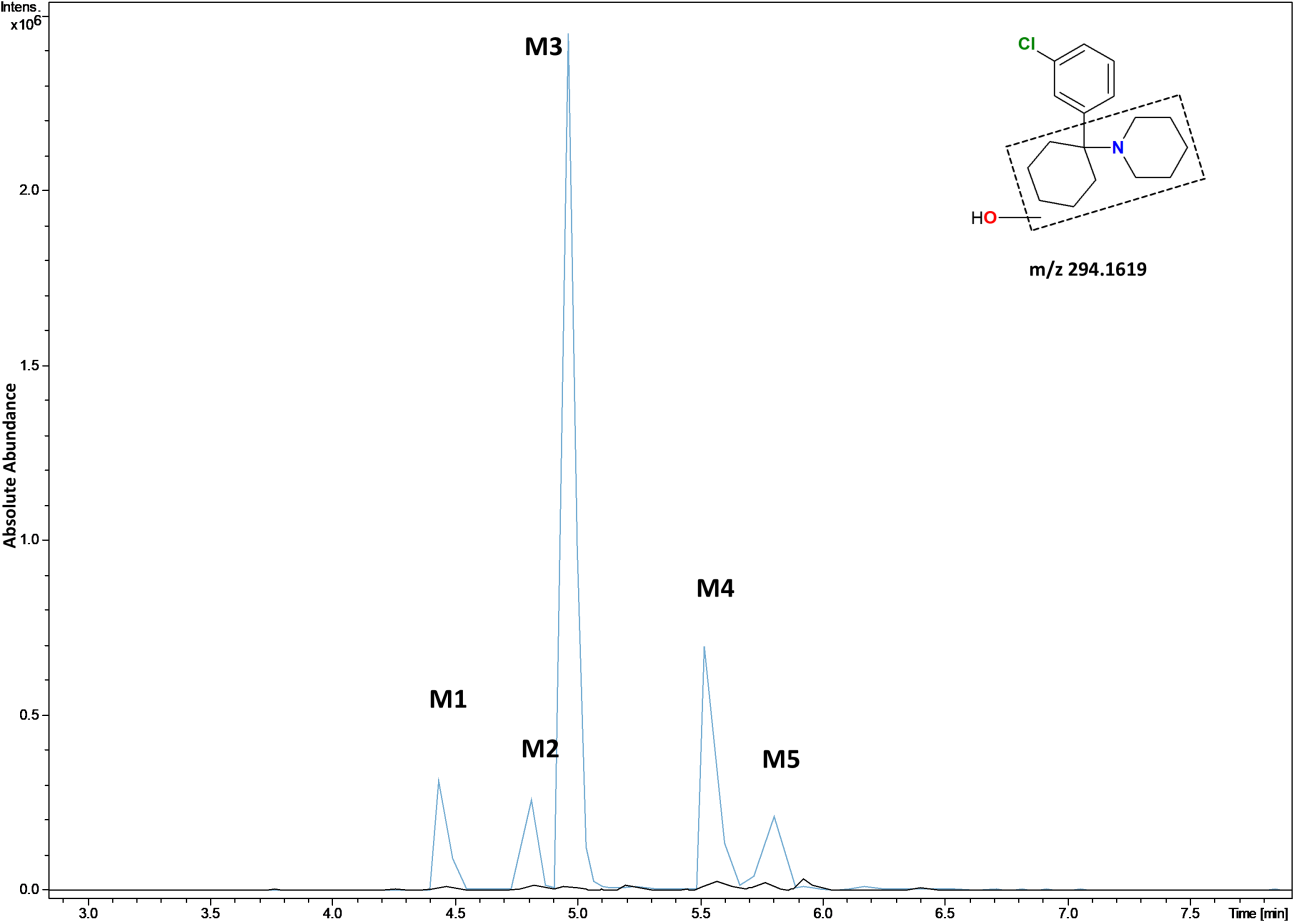
Extracted ion chromatogram (EIC) corresponding to the exact mass of a monooxygenated metabolite *m/z* 294.1618. The black baseline represents the EIC for *m/z* 294.1618 without microsome incubation.

The monooxygenation reaction is examined by analyzing the extracted ion chromatogram (EIC) corresponding to the exact mass of a monooxygenated compound (*m/z* 294.1618 ± 0.005). The EIC reveals the presence of five distinct peaks (Figure 3). A closer inspection of the fragmentation spectra indicates that only two characteristic fragmentation patterns are observed among these five peaks. Based on the fragment ions, it can be inferred that three different hydroxy cyclohexyl metabolites (M1–M3, arranged in order of increasing retention times) and two different hydroxy piperidine metabolites (M4 and M5) are formed. These metabolites are discussed in detail in this section.

**FIGURE 4:**
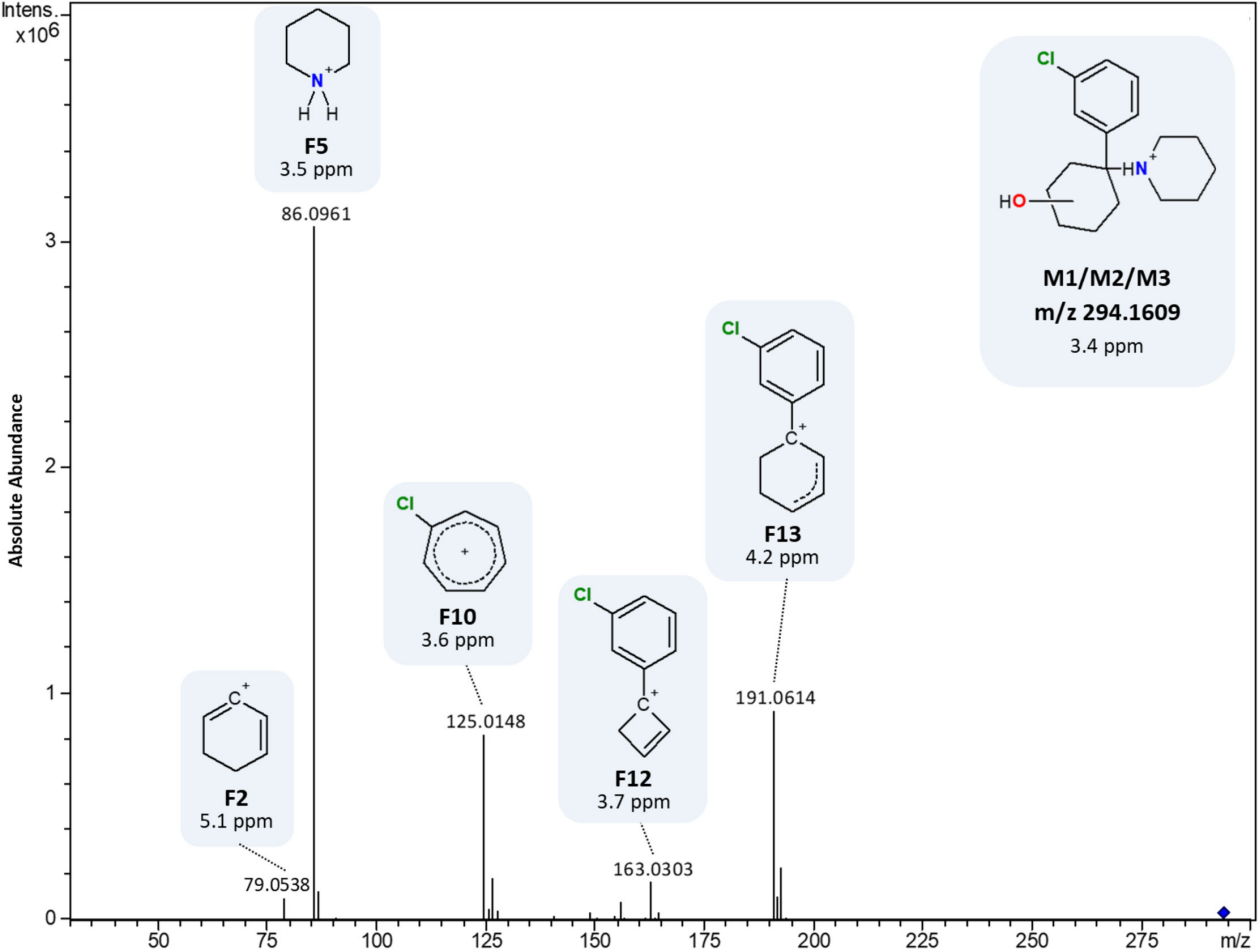
Representative MS/MS fragment spectrum of the hydroxy cyclohexyl metabolite M3 at a retention time of 5.0 min. The suggested fragment structures are annotated with their mass differences, calculated in parts per million relative to the measured masses. The dotted line in fragment **F13** indicates that the exact position of the double bond remains unresolved. Importantly, the positive charge displayed in the fragments represents only one possible location, as charge redistribution (e.g., due to rearrangements) could result in alternative patterns. Metabolites M1 and M2 exhibit a structurally analogous fragment spectrum (not shown).

From the proposed hydroxy cyclohexyl metabolites (M1 at 4.4 min, M2 at 4.8 min, and M3 at 5.0 min), several characteristic fragment ions were tentatively identified (Figure 4). These include a piperidinium cation (**F5**), a chlorotropylium cation (**F10**), a chlorophenyl cyclohexene cation (**F13**), and a chlorophenyl cyclobutene cation (**F12**). The structurally unchanged presence of the piperidinium cation and the chlorotropylium cation suggests that neither the piperidine moiety nor the aromatic ring were targets of hydroxylation. Consequently, hydroxylation likely occurred on the cyclohexane ring. This conclusion is further supported by the formation of fragments **F12** and **F13**, which may result from dehydration (elimination of water) during fragmentation. Similar to the parent compound, fragments **F13**, **F12**, and **F10** likely originate from the lighter chlorine isotope ^35^Cl. Their corresponding heavier counterparts, arising from ^37^Cl, appear at *m/z* 193.0586 (3.6 ppm), *m/z* 165.0274 (3.6 ppm), and *m/z* 127.0119 (3.1 ppm), respectively.

**FIGURE 5:**
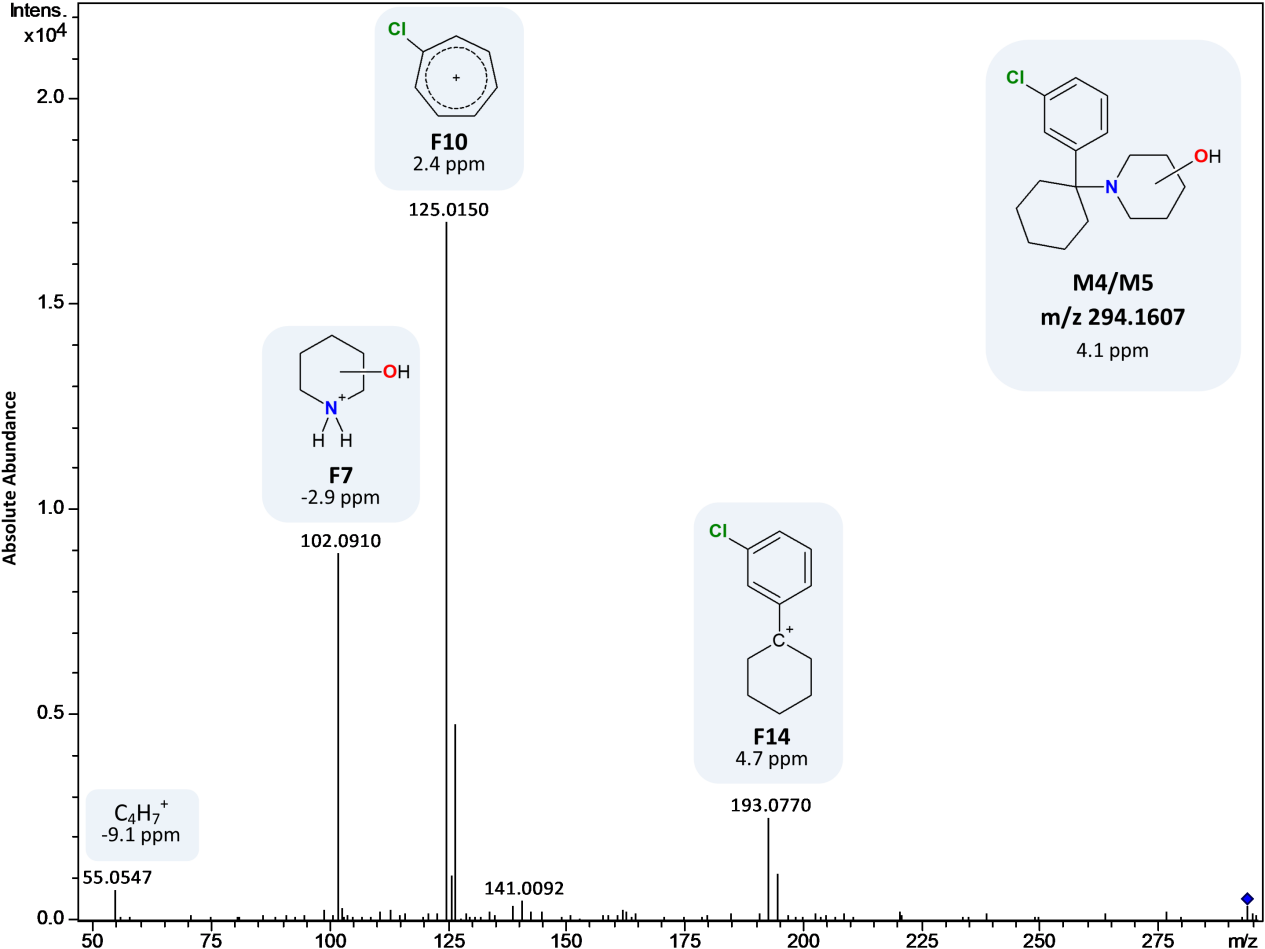
Representative MS/MS fragment spectrum of the hydroxy piperidine metabolite M4 at a retention time of 5.6 min. The suggested fragment structures are annotated with their mass differences, calculated in parts per million relative to the measured masses. Importantly, the positive charge displayed in the fragments represents only one possible location, as charge redistribution (e.g., due to rearrangements) could result in alternative patterns. Metabolite M5 exhibits a structurally analogous fragment spectrum (not shown).

Of the two proposed hydroxy piperidine metabolites M4 and M5 (with retention times of 5.6 and 5.8 min, respectively, Figure 5), a hydroxy piperidinium fragment (**F7**) could be formed. This suggests that the piperidine ring may have undergone hydroxylation. Furthermore, the detection of the chlorotropylium cation (**F10**) and the chlorophenyl cyclohexyl cation (**F14**) indicates that both the cyclohexane portion and the aromatic portion of the metabolite remain unchanged.

#### 3.2.3 Piperidine Ring‐Opened Metabolites M6 and M7

**FIGURE 6:**
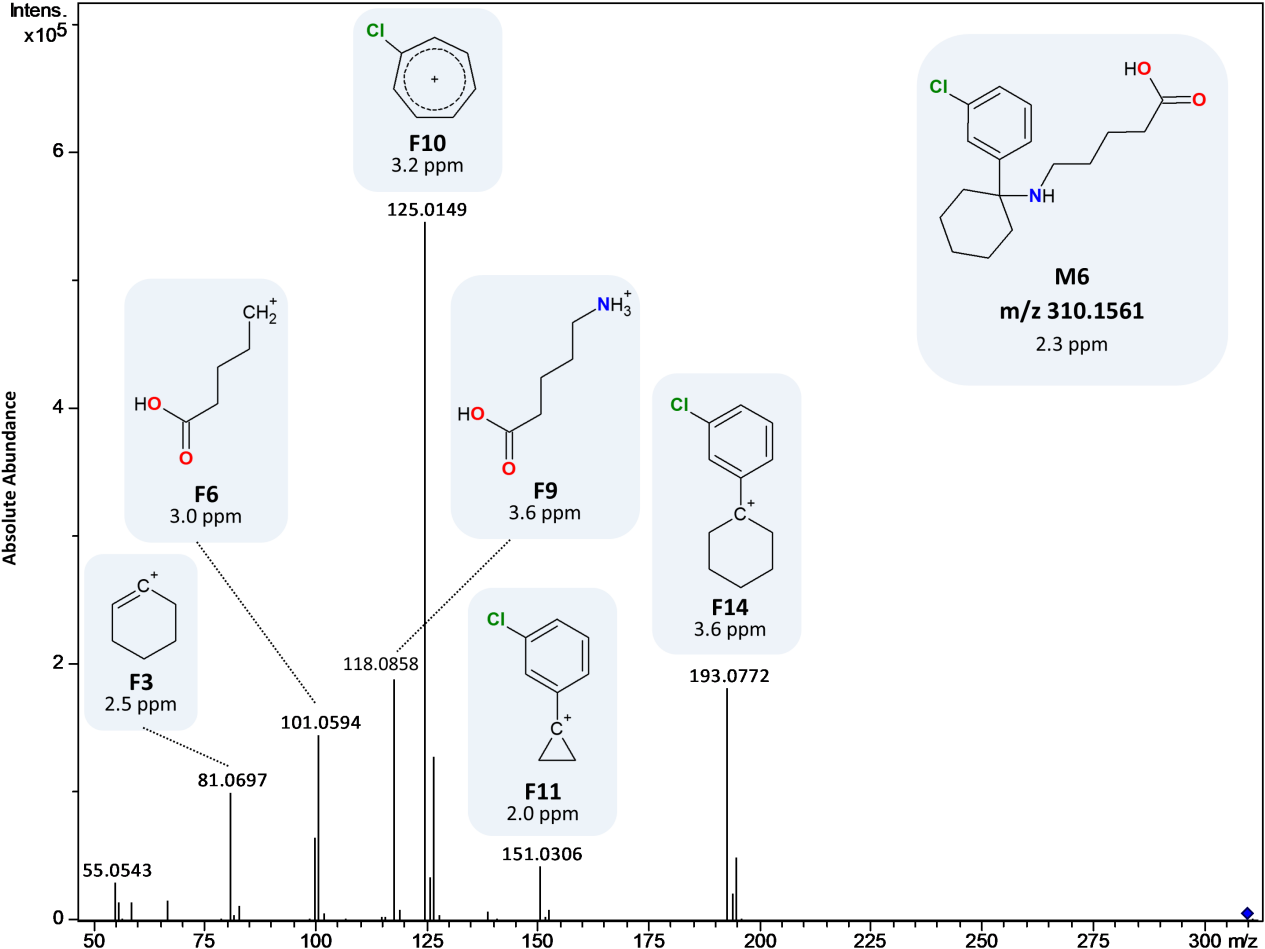
MS/MS fragment spectrum of the metabolite M6 at a retention time of 6.5 min. The suggested fragment structures are annotated with their mass differences, calculated in parts per million relative to the measured masses. Importantly, the positive charge displayed in the fragments represents only one possible location, as charge redistribution (e.g., due to rearrangements) could result in alternative patterns.

The proposed molecular formula for metabolite M6 is C_17_H_24_ClNO_2_ (*m/z* 310.1561, retention time 6.5 min), which formally suggests carboxylation of the parent compound (Figure 6). Notably, both the chlorotropylium cation (**F10**) and the chlorophenyl cyclohexyl cation (**F14**) remain structurally unchanged. Furthermore, the detection of the pentanoate cation (**F6**) and the 5‐aminopentanoate fragment (**F9**) suggests a ring opening of the piperidine ring and terminal carboxylation. This metabolic pathway was also predicted in silico by EAWAG‐BBD (Section 3.1).

**FIGURE 7:**
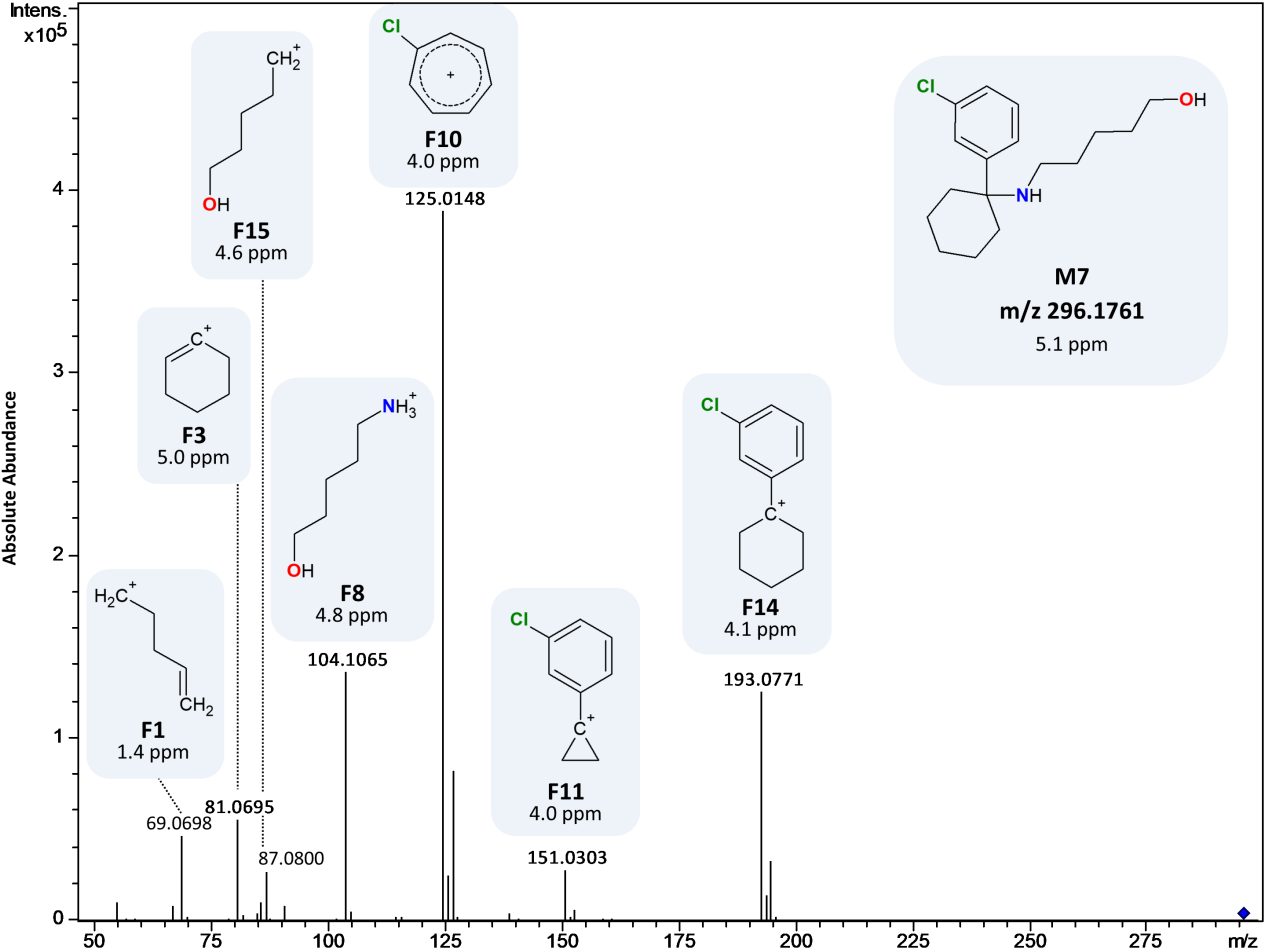
MS/MS fragment spectrum of the metabolite M7 at a retention time of 6.2 min. The suggested fragment structures are annotated with their mass differences, calculated in parts per million relative to the measured masses. Importantly, the positive charge displayed in the fragments represents only one possible location, as charge redistribution (e.g., due to rearrangements) could result in alternative patterns.

Metabolite M7 (*m/z* 296.1761, retention time 6.2 min) shares several mass fragments in its MS/MS spectrum with M6 (Figure 7). Specifically, the presence of fragments **F10**, **F11**, and **F14** in both spectra suggests that M6 and M7 share the same structural scaffold. Similar to fragments **F6** and **F9** for the metabolite M6, the 5‐aminopentanol cation (**F8**) and the pentanol fragment (**F15**) observed for M7 indicate a ring opening of the piperidine ring. In contrast to M6, these fragments suggest that hydroxylation occurs at the resulting carbon chain. The pentanol fragment **F15** may result from the loss of ammonia from the 5‐aminopentanol cation **F8**. Furthermore, a subsequent loss of water could produce the corresponding pentene cation **F1**. Interestingly, an additional measurement using the same LC gradient on the C_18_ column revealed that, among all metabolites, only M6 and M7 eluted later than the parent compound (6.5 and 6.2 min, compared to 6.1 min for the parent). This elution order is unusual and will be discussed in Section 3.3.3.

### 3.3 Metabolite Comparison in Different Postmortem Samples

To investigate human metabolism, the LC–MS/MS method was employed. After an initial qualitative screening analysis had been conducted, an MRM method was developed to evaluate the relative abundance of the metabolites (Tables S3 and S4). For most metabolites, only the quantifier and one qualifier transition were used. However, when a transition was shared among several metabolites, an additional transition (resulting in two qualifiers) was measured to ensure specificity. Several matrices were examined, including urine and bile (both before and after β‐glucuronidase hydrolysis), as well as cardiac blood, femoral blood, and gastric contents. For metabolite identification, the guidelines of the German Society of Toxicological and Forensic Chemistry (GTFCh) were followed [38]. Most metabolites were fully separated chromatographically. However, the two hydroxy piperidine metabolites, M4 and M5, could not be baseline separated.

All metabolites that had been preliminarily identified in vitro were also detected in human biosamples. The corresponding chromatograms can be found in Figures S1–S32. Notably, metabolites M4–M7 were qualitatively detected in all matrices analyzed. However, dechlorination to PCP, a dehydro piperidine metabolite, a hydroxylated carboxyl metabolite, and other *N‐*dealkylated metabolites were not observed [5]. In the following sections, each metabolite and its occurrence in the respective matrices will be presented.

#### 3.3.1 Tissue Distribution of Hydroxy Cyclohexyl Metabolites M1–M3

**FIGURE 8:**
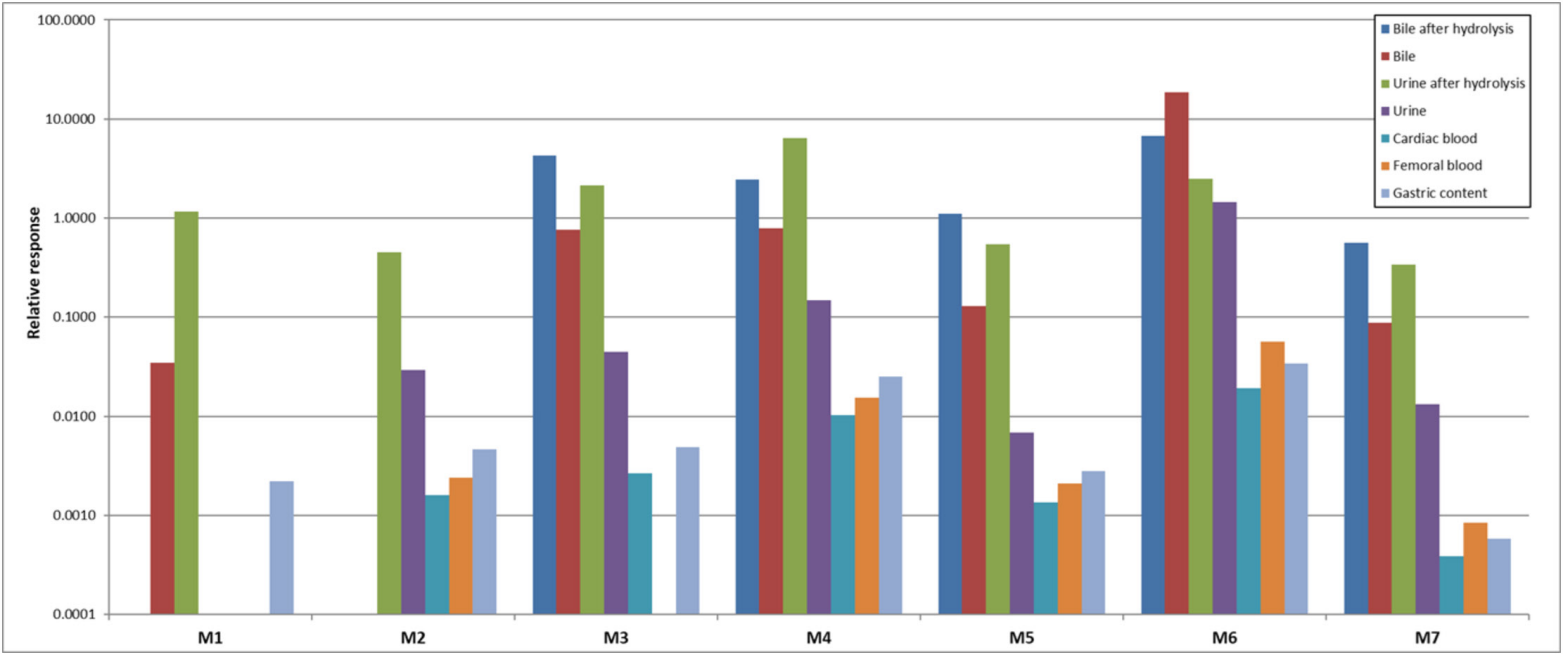
Logarithmic representation of the relative response for all metabolites in each matrix where the metabolite was qualitatively detected. The relative response is defined as the ratio of the peak area of the respective MRM transition (as described in the text) to the peak area of the internal standard, PCP‐d_5_.

The proposed hydroxy cyclohexyl metabolite M1 could only be qualitatively detected in the gastric contents, urine (after β‐glucuronidase hydrolysis), and bile. The relative response for the MRM transition 294.2 → 86.1 (normalized to the peak area of the internal standard PCP‐d_5_, Figure 8 and Table S5) was 3.45 × 10^−2^ in bile, 1.17 in urine, and 2.19 × 10^−3^ in gastric content. Particularly, M1 could not be detected in cardiac blood or in bile after β‐glucuronidase hydrolysis.

The postulated hydroxy cyclohexyl metabolite M2 could be detected qualitatively in urine—both before and after glucuronide cleavage—as well as in cardiac blood, femoral blood, and gastric contents. However, in bile, M2 was not detectable, regardless of whether glucuronide cleavage has been performed. The relative response for the same normalized MRM transition was 0.45 in urine after glucuronide cleavage, 2.92 × 10^−2^ in urine without hydrolysis, 1.60 × 10^−3^ in cardiac blood, 2.41 × 10^−3^ in femoral blood, and 4.68 × 10^−3^ in gastric contents (Figure 8).

The third proposed hydroxy cyclohexyl metabolite M3 was qualitatively detected in all examined matrices except for femoral blood. After β‐glucuronidase hydrolysis, the relative response for the same normalized MRM transition was 4.26 in bile and 2.13 in urine. In contrast, without hydrolysis, the relative response was 0.76 in bile and only 4.49 × 10^−2^ in urine. Additionally, the relative responses were 2.65 × 10^−3^ in cardiac blood and 4.91 × 10^−3^ in gastric contents (Figure 8).

Interestingly, M1 was present in bile without β‐glucuronidase hydrolysis, albeit at low abundance. After β‐glucuronidase incubation, M1 was not detected. This finding suggests that no substantial amounts of M1 glucuronide are present in bile. Furthermore, it is likely that the β‐glucuronidase hydrolysis step caused a dilution of M1 in bile. Combined with potential matrix effects, this might have led to a reduction of the abundance below the limit of detection.

In contrast, M1 was not detected in urine without β‐glucuronidase hydrolysis. However, after hydrolysis, M1 was observed in urine at the highest intensity among all matrices. This result strongly suggests extensive in vivo glucuronidation and kidney filtration. Generally, glucuronides can be excreted either via bile or urine; in this case, M1 glucuronide seems to be primarily excreted in urine. Although glucuronidation has been identified as one metabolic pathway, other phase II metabolic pathways were not investigated in this study.

In addition to the monooxygenated metabolites described, other metabolites with the same exact mass are plausible. For instance, a potential *N‐*oxide metabolite would share the same molecular formula and exact mass. Although the formation of hydroxy piperidine metabolites appears more likely due to steric considerations, the fragment spectra do not allow for a definitive distinction between *N‐*oxide metabolites and hydroxy piperidine metabolites.

Similar to M1, M2 is not primarily excreted unchanged. Instead, it appears to be excreted after conjugation with glucuronic acid or via other metabolic pathways that have not yet been investigated. Based on the data collected, M2 glucuronide is mainly excreted renally.

Notably, M3 is the only metabolite that, after β‐glucuronidase cleavage, is present in bile at abundances similar to those found in urine following the same treatment. This finding suggests that the M3 glucuronide may have an affinity for a biliary transporter and is at least partially excreted via the bile.

#### 3.3.2 Tissue Distribution of Hydroxy Piperidine Metabolites M4 and M5

The postulated hydroxy piperidine metabolite M4 was qualitatively detected in all tested matrices. After β‐glucuronidase hydrolysis, the normalized relative response for the MRM transition 294.2 → 102.1 increased to 2.46 in bile and 6.44 in urine. In contrast, without hydrolysis, the relative response was much lower: 0.79 in bile and 0.15 in urine. Additionally, the relative responses in blood and gastric contents were minimal, measured at 1.03 × 10^−2^ in cardiac blood, 1.53 × 10^−2^ in femoral blood, and 2.53 × 10^−2^ in gastric contents (Figure 8 and Table S5).

The second proposed hydroxy piperidine metabolite M5 exhibits a distribution and metabolic profile similar to that of M4. Notably, after glucuronide cleavage, the normalized relative response of M5 for the same MRM transition was 1.1 in bile and 0.54 in urine. However, without glucuronide cleavage, the relative response decreased substantially, reaching 0.12 in bile and 6.79 × 10^−3^ in urine. Furthermore, the relative responses of M5 in cardiac blood, femoral blood, and gastric contents were 1.35 × 10^−3^, 2.09 × 10^−3^, and 2.81 × 10^−3^, respectively (Figure 8).

Notably, similar to metabolite M3, the relative response of M4 increased by nearly 1 order of magnitude in bile and by 1 order of magnitude in urine after β‐glucuronidase treatment. Furthermore, when compared to the second postulated hydroxy piperidine metabolite, M5, M4 exhibited larger peak areas (Figure 8). These findings suggest that M4 is likely excreted partly unchanged via the bile and, following glucuronidation, is also present in both bile and urine. It is important to note that the highest abundances of M4 glucuronide were detected in urine.

Focusing on M5, the abundance patterns suggest that a portion of M5 is excreted unchanged via the bile. After glucuronidation, M5 is eliminated through both bile and urine. Notably, the highest abundance of M5 glucuronide was observed in bile, whereas the levels in urine were only slightly lower—by less than 1 order of magnitude.

#### 3.3.3 Tissue Distribution of Piperidine Ring‐Opened Metabolites M6 and M7

The piperidine ring‐opened carboxy metabolite M6 was detected in all analyzed matrices. After β‐glucuronidase hydrolysis, the normalized relative response for M6 for the MRM transition 310.2 → 118.1 was 6.79 in bile and 2.50 in urine. In contrast, without hydrolysis, the relative response was much higher in bile (18.54) and lower in urine (1.46). Additionally, the relative responses in blood and gastric content were consistently low, measured at 1.91 × 10^−2^ in cardiac blood, 5.64 × 10^−2^ in femoral blood, and 3.41 × 10^−2^ in gastric content (Figure 8 and Table S5).

Similar to metabolites M4, M5, and M6, the piperidine ring‐opened alcohol metabolite M7 was detected in all tested matrices. In particular, M7 exhibited its highest intensity in bile—both before and after β‐glucuronidase cleavage—as well as in urine after β‐glucuronidase treatment. After hydrolysis with β‐glucuronidase, the normalized relative response for M7 for the MRM transition 296.2 → 104.1 was 0.56 in bile and 0.33 in urine. In contrast, following glucuronide cleavage, the relative response dropped substantially, reaching only 8.74 × 10^−2^ in bile and 1.32 × 10^−4^ in urine. Among all metabolites, M7 showed the lowest relative responses in cardiac blood, femoral blood, and gastric contents, with values of just 3.88 × 10^−4^, 8.42 × 10^−4^, and 5.79 × 10^−4^, respectively (Figure 8).

The highest abundances of M6 were observed in bile fluid without β‐glucuronidase treatment. This was followed by bile fluid and urine after β‐glucuronidase treatment. Notably, the abundance of M6 in bile fluid decreased after β‐glucuronidase treatment, suggesting that significant biliary secretion of the glucuronide is unlikely. Similarly, the abundance concentration in urine did not increase substantially following β‐glucuronidase treatment, indicating that M6 does not undergo significant glucuronidation in vivo.

In contrast, metabolites M1–M5 showed a substantial increase in urine abundance by approximately 1–2 orders of magnitude after β‐glucuronidase treatment. This finding indicates that, unlike M1–M5, M6 does not form glucuronides to a substantial extent. The chemoselectivity of UDP‐glucuronosyltransferases (UGTs) may contribute to this observation. UGTs typically bind alcohols as substrates [39, 40], as seen with metabolites M1–M5, whereas M6 contains a carboxy group. Furthermore, acyl glucuronides are relatively reactive due to their ester and acetal functionalities; therefore, hydrolysis during sample processing or instability of these compounds is also conceivable [41, 42, 43].

In Section 3.2.3, two hydroxy piperidine metabolites were identified. However, three positional isomers are theoretically possible, corresponding to hydroxylation at the 2‐, 3‐, or 4‐position of the piperidine ring. The 2‐hydroxy piperidine represents a cyclic hemiaminal, which exists in a tautomeric equilibrium with the corresponding secondary amine and the open‐chain aldehyde [34]. It is therefore plausible that all three positional isomers are formed, but the hemiaminal may undergo ring opening through tautomerization. The literature also reports α‐hydroxylation of piperidines, followed by ring opening and subsequent metabolic conversion to the corresponding alcohol or carboxylic acid [34]. Because aldehydes are electrophilic and exhibit high metabolic reactivity, they are often difficult to detect due to their rapid conversion into other metabolites [44, 45]. Consequently, direct observation of aldehyde intermediates cannot be easily achieved. Alternatively, dehydration could lead to the formation of a dehydro piperidine metabolite [5]. However, such a reaction was not observed in this study.

Human metabolism typically promotes the hydrophilic modification of xenobiotics, thereby facilitating their excretion [46]. Consequently, metabolites are generally expected to exhibit lower retention than the parent compound when analyzed using reversed‐phase liquid chromatography (LC) columns, as commonly employed in forensic toxicology [47, 48, 49]. This expectation was confirmed for all metabolites analyzed on the biphenyl column in the present study. In contrast, metabolites M6 and M7 demonstrated greater retention than the parent compound, determined through additional measurements on a C_18_ column. This observation suggests that these metabolites may possess open‐chain structures, which enable stronger van‐der‐Waals interactions between their unbranched carbon chains and the octadecyl groups of the stationary phase [50, 51, 52]. Despite their hydrophilic functionalization, such interactions could ultimately result in increased retention. The retention time thus serves as a valuable orthogonal parameter to the fragment spectra, providing complementary information that can support metabolite structure proposals [53].

**FIGURE 9:**
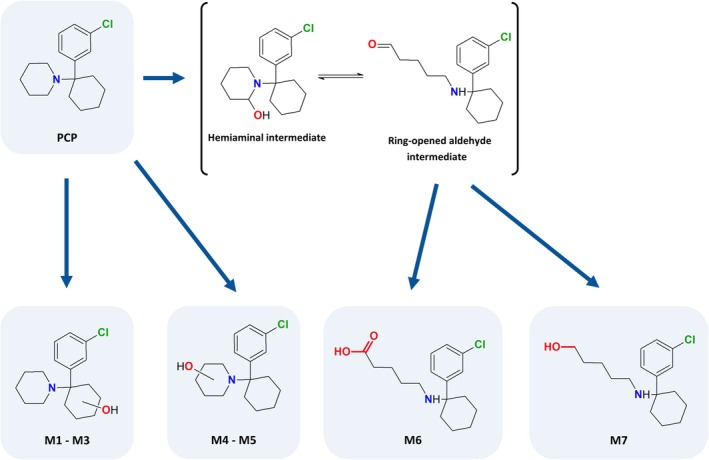
Proposed metabolic fate of 3‐Cl‐PCP.

Based on the results, the proposed biotransformation of 3‐Cl‐PCP is depicted in Figure 9. Potential hydroxylation occurs either at the cyclohexyl ring (metabolites M1–M3) or at the piperidine ring (M4–M5). In addition, a potential alpha‐hydroxylation at the piperidine ring (forming a hemiaminal) may lead to a ring opening and formation of the corresponding aldehyde intermediate. Subsequent oxidation could yield the open‐chain carboxylic acid (M6), whereas reduction could produce the respective alcohol (M7).

### 3.4 3‐Cl‐PCP Concentration in Postmortem Samples Determined by Standard Addition

The concentration of 3‐Cl‐PCP in the postmortem samples was determined using the standard addition method. For measurements conducted without β‐glucuronidase hydrolysis, both urine and bile matrices were diluted 1:10 with deionized water, based on results of preliminary concentration determinations. In contrast, during the experimental procedures involving β‐glucuronidase hydrolysis, only the bile samples were diluted 1:10, using a phosphate buffer at pH 6 instead of deionized water. To calculate the final concentration, the absolute value of the *x*‐intercept from the calibration curve was multiplied by the dilution factor. The resulting concentrations are summarized in Table 2. To achieve an acceptable correlation coefficient (*R* ^2^ ≥ 0.99 according to Hasegawa et al. [54]), one value was eliminated in each case, except for the femoral blood samples.

**TABLE 2:** Dilution factors and measured concentrations (ng/mL) of 3‐Cl‐PCP in various matrices.

Matrix	Concentration of 3‐Cl‐PCP (ng/mL)	Dilution factor	Linear regression equation	Correlation coefficient *R* ^2^
Cardiac blood	610	50	*y* = 0.1773*x* + 2.1643	0.9921
Femoral blood	830	50	*y* = 0.1451*x* + 2.4243	0.9993
Gastric content	840	50	*y* = 0.1733*x* + 2.9246	0.9917
Urine	1200	500	*y* = 0.2020*x* + 0.4010	0.9984
Urine after β‐glucuronidase cleavage	730	122.5	*y* = 2.7028*x* + 16.18	0.9915
Bile	4300	500	*y* = 0.2532*x* + 2.1921	0.9999
Bile after β‐glucuronidase cleavage	2200	1225	*y* = 3.0659*x* + 5.5994	0.9994

### 3.5 Detection in Syringe Remnants

In the present case, three disposable syringes were analyzed to determine whether the substance 3‐Cl‐PCP had been administered intravenously. To address this question, the remnants of the syringes were examined. For the analysis, external calibration was carried out using a reference standard, following the guidelines of the GTFCh [38]. The concentrations of 3‐Cl‐PCP detected in the remnants of the three syringes were found to be 34.4, 55.8, and 16.5 μg/mL, respectively.

### 3.6 Limitations

A key limitation of this study lies in the fact that the measured signal abundances are not directly equal to absolute metabolite concentrations due to matrix effects. These effects, resulting from the complex composition of the biological sample, can influence ionization efficiency and lead to signal suppression or enhancement. Although the internal standard PCP‐d_5_ was used to correct for these matrix effects, its slightly different chemical structure compared to the metabolites prevents it from fully compensating for compound‐specific matrix effects. Therefore, the obtained metabolite signal intensities should be considered relative rather than absolute measures of concentration.

## 4 Conclusion

This study portrayed the human metabolism of the novel designer dissociative 3‐Cl‐PCP, aided by a pHLM assay, in silico models, and the investigation of postmortem biosamples from a 3‐Cl‐PCP fatality. The pHLM model predicted seven phase I metabolites, whereas phase II metabolites could not be assessed due to a lack of UGT in the assay. In vitro analyses and investigations on postmortem biosamples confirmed the key metabolic pathways, including monooxygenation and piperidine ring opening. For urine analysis, priority should be given to the hydroxy cyclohexyl (M3), hydroxy piperidine (M4), and piperidine ring‐opened carboxyl (M6) metabolites, with a preceding β‐glucuronidase cleavage step. Together with the parent compound, these metabolites could serve as the most valuable biomarkers for toxicological assessment. Glucuronidation seems to play a substantial role in excretion, primarily via urine. Understanding tissue distribution and excretion pathways can be crucial for clinical and forensic applications and should be further investigated.

## Conflicts of Interest

The authors declare no conflicts of interest.

## Supporting information


**Table S1:** Ion inclusion list for analysis of the pHLM assay by QTOF.
**Table S2:** List of fragment ions with ID and postulated structure in high‐resolution MS/MS spectra.
**Table S3:** Broad‐spectrum screening MRM method for detecting in vivo metabolites of 3‑Cl‑PCP (DP, declustering potential; EP, entrance potential; CE, collision energy; CXP, cell exit potential).
**Table S4:** Optimized MRM method for the determination of peak areas of detected in vivo metabolites (DP, declustering potential; EP, entrance potential; CE, collision energy; CXP, cell exit potential).
**Table S5:** Peak areas obtained from MS/MS analysis of the respective matrix (cardiac blood, bile, and urine). The acceptance criteria were established in accordance with GTFCh (German Society of Toxicology & Forensic Chemistry) guidelines, using pooled human liver microsomes (pHLM) metabolites as reference. Peak areas highlighted in yellow fall outside the predefined acceptance range.
**Figure S1:** Chromatogram of the three transitions of the hydroxycyclohexyl metabolites M1–3 from the pHLM incubation. (enlarged; blue: 294.2 → 125.0, red: 294.2 → 163.0, green: 294.2 → 86.1).
**Figure S2:** Chromatogram of the three transitions of the hydroxycyclohexyl metabolites M1–3 in bile after β‐glucuronidase hydrolysis (enlarged; blue: 294.2 → 125.0, red: 294.2 → 163.0, green: 294.2 → 86.1).
**Figure S3:** Chromatogram of the three transitions of the hydroxycyclohexyl metabolites M1–3 in bile without β‐glucuronidase hydrolysis (enlarged; blue: 294.2 → 125.0, red: 294.2 → 163.0, green: 294.2 → 86.1).
**Figure S4:** Chromatogram of the three transitions of the hydroxycyclohexyl metabolites M1–3 in urine after β‐glucuronidase hydrolysis (enlarged; blue: 294.2 → 125.0, red: 294.2 → 163.0, green: 294.2 → 86.1).
**Figure S5:** Chromatogram of the three transitions of the hydroxycyclohexyl metabolites M1–3 in urine without β‐glucuronidase hydrolysis (enlarged; blue: 294.2 → 125.0, red: 294.2 → 163.0, green: 294.2 → 86.1).
**Figure S6:** Chromatogram of the three transitions of the hydroxycyclohexyl metabolites M1–3 in cardiac blood (enlarged; blue: 294.2 → 125.0, red: 294.2 → 163.0, green: 294.2 → 86.1).
**Figure S7:** Chromatogram of the three transitions of the hydroxycyclohexyl metabolites M1–3 in femoral blood (enlarged; blue: 294.2 → 125.0, red: 294.2 → 163.0, green: 294.2 → 86.1).
**Figure S8:** Chromatogram of the three transitions of the hydroxycyclohexyl metabolites M1–3 in gastric content (enlarged; blue: 294.2 → 125.0, red: 294.2 → 163.0, green: 294.2 → 86.1).
**Figure S9:** Chromatogram of the three transitions of the hydroxypiperidine metabolites M4–5 from the pHLM incubation (enlarged; blue: 294.2 → 125.0, red: 294.2 → 193.1, green: 294.2 → 102.1).
**Figure S10:** Chromatogram of the three transitions of the hydroxypiperidine metabolites M4–5 in bile after β‐glucuronidase hydrolysis (enlarged; blue: 294.2 → 125.0, red: 294.2 → 193.1, green: 294.2 → 102.1).
**Figure S11:** Chromatogram of the three transitions of the hydroxypiperidine metabolites M4–5 in bile without β‐glucuronidase hydrolysis (enlarged; blue: 294.2 → 125.0, red: 294.2 → 193.1, green: 294.2 → 102.1).
**Figure S12:** Chromatogram of the three transitions of the hydroxypiperidine metabolites M4–5 in urine after β‐glucuronidase hydrolysis (enlarged; blue: 294.2 → 125.0, red: 294.2 → 193.1, green: 294.2 → 102.1).
**Figure S13:** Chromatogram of the three transitions of the hydroxypiperidine metabolites M4–5 in urine without β‐glucuronidase hydrolysis (enlarged; blue: 294.2 → 125.0, red: 294.2 → 193.1, green: 294.2 → 102.1).
**Figure S14:** Chromatogram of the three transitions of the hydroxypiperidine metabolites M4–5 in cardiac blood (enlarged; blue: 294.2 → 125.0, red: 294.2 → 193.1, green: 294.2 → 102.1).
**Figure S15:** Chromatogram of the three transitions of the hydroxypiperidine metabolites M4–5 in femoral blood (enlarged; blue: 294.2 → 125.0, red: 294.2 → 193.1, green: 294.2 → 102.1).
**Figure S16:** Chromatogram of the three transitions of the hydroxypiperidine metabolites M4–5 in gastric content (enlarged; blue: 294.2 → 125.0, red: 294.2 → 193.1, green: 294.2 → 102.1).
**Figure S17:** Chromatogram of the two transitions of the carboxyl metabolite M6 from the pHLM incubation (enlarged; blue: 310.2 → 193.0, red: 310.2 → 118.1).
**Figure S18:** Chromatogram of the two transitions of the carboxyl metabolite M6 in bile after β‐glucuronidase hydrolysis (enlarged; blue: 310.2 → 193.0, red: 310.2 → 118.1).
**Figure S19:** Chromatogram of the two transitions of the carboxyl metabolite M6 in bile without β‐glucuronidase hydrolysis (enlarged; blue: 310.2 → 193.0, red: 310.2 → 118.1).
**Figure S20:** Chromatogram of the two transitions of the carboxyl metabolite M6 in urine after β‐glucuronidase hydrolysis (enlarged; blue: 310.2 → 193.0, red: 310.2 → 118.1).
**Figure S21:** Chromatogram of the two transitions of the carboxyl metabolite M6 in urine without β‐glucuronidase hydrolysis (enlarged; blue: 310.2 → 193.0, red: 310.2 → 118.1).
**Figure S22:** Chromatogram of the two transitions of the carboxyl metabolite M6 in cardiac blood (enlarged; blue: 310.2 → 193.0, red: 310.2 → 118.1).
**Figure S23:** Chromatogram of the two transitions of the carboxyl metabolite M6 in femoral blood (enlarged; blue: 310.2 → 193.0, red: 310.2 → 118.1).
**Figure S24:** Chromatogram of the two transitions of the carboxyl metabolite M6 in gastric content (enlarged; blue: 310.2 → 193.0, red: 310.2 → 118.1).
**Figure S25:** Chromatogram of the two transitions of the alcohol metabolite M7 from the pHLM incubation (enlarged; blue: 296.2 → 125.0, red: 296.2 → 104.1).
**Figure S26:** Chromatogram of the two transitions of the alcohol metabolite M7 in bile with β‐glucuronidase hydrolysis (enlarged; blue: 296.2 → 125.0, red: 296.2 → 104.1).
**Figure S27:** Chromatogram of the two transitions of the alcohol metabolite M7 in bile without β‐glucuronidase hydrolysis (enlarged; blue: 296.2 → 125.0, red: 296.2 → 104.1).
**Figure S28:** Chromatogram of the two transitions of the alcohol metabolite M7 in urine with β‐glucuronidase hydrolysis (enlarged; blue: 296.2 → 125.0, red: 296.2 → 104.1).
**Figure S29:** Chromatogram of the two transitions of the alcohol metabolite M7 in urine without β‐glucuronidase hydrolysis (enlarged; blue: 296.2 → 125.0, red: 296.2 → 104.1).
**Figure S30:** Chromatogram of the two transitions of the alcohol metabolite M7 in cardiac blood (enlarged; blue: 296.2 → 125.0, red: 296.2 → 104.1).
**Figure S31:** Chromatogram of the two transitions of the alcohol metabolite M7 in femoral blood (enlarged; blue: 296.2 → 125.0, red: 296.2 → 104.1).
**Figure S32:** Chromatogram of the two transitions of the alcohol metabolite M7 in gastric content (enlarged; blue: 296.2 → 125.0, red: 296.2 → 104.1).

## Data Availability

The data that support the findings of this study are available in the Supporting Information.
