# Supplementary material for: Phase I Metabolism of Novel Phencyclidine Derivative 3‐Cl‐PCP: In Vitro Studies With Pooled Human Liver Microsomes and Investigation of a Post‐Mortem Case
Source: Drug Test Anal. 2026 May 24;18(8):1036–53. doi: 10.1002/dta.70097 (PMC13432771; doi:10.1002/dta.70097)
Supplement: Supplementary file 1 — Table S1: Ion inclusion list for analysis of the pHLM assay by QTOF. Table S2: List of fragment ions with ID and postulated structure in high‐resolution MS/MS spectra. Table S3: Broad‐spectrum screening MRM method for detecting in vivo metabolites of 3‑Cl‑PCP (DP, declustering potential; EP, entrance potential; CE, collision energy; CXP, cell exit potential). Table S4: Optimized MRM method for the determination of peak areas of detected in vivo metabolites (DP, declustering potential; EP, entrance potential; CE, collision energy; CXP, cell exit potential). Table S5: Peak areas obtained from MS/MS analysis of the respective matrix (cardiac blood, bile, and urine). The acceptance criteria were established in accordance with GTFCh (German Society of Toxicology & Forensic Chemistry) guidelines, using pooled human liver microsomes (pHLM) metabolites as reference. Peak areas highlighted in yellow fall outside the predefined acceptance range. Figure S1: Chromatogram of the three transitions of the hydroxycyclohexyl metabolites M1–3 from the pHLM incubation. (enlarged; blue: 294.2 → 125.0, red: 294.2 → 163.0, green: 294.2 → 86.1). Figure S2: Chromatogram of the three transitions of the hydroxycyclohexyl metabolites M1–3 in bile after β‐glucuronidase hydrolysis (enlarged; blue: 294.2 → 125.0, red: 294.2 → 163.0, green: 294.2 → 86.1). Figure S3: Chromatogram of the three transitions of the hydroxycyclohexyl metabolites M1–3 in bile without β‐glucuronidase hydrolysis (enlarged; blue: 294.2 → 125.0, red: 294.2 → 163.0, green: 294.2 → 86.1). Figure S4: Chromatogram of the three transitions of the hydroxycyclohexyl metabolites M1–3 in urine after β‐glucuronidase hydrolysis (enlarged; blue: 294.2 → 125.0, red: 294.2 → 163.0, green: 294.2 → 86.1). Figure S5: Chromatogram of the three transitions of the hydroxycyclohexyl metabolites M1–3 in urine without β‐glucuronidase hydrolysis (enlarged; blue: 294.2 → 125.0, red: 294.2 → 163.0, green: 294.2 → 86.1). Figure S6: Chromatogram of th [file DTA-18-1036-s001.docx]

**Phase I Metabolism of Novel Phencyclidine Derivative 3-Cl-PCP: *In Vitro* Studies with Pooled Human Liver Microsomes and Investigation of a Post-Mortem Case**

Johannes Kutzler^1,2,3^, Tobias Veit^1,2^, Thomas Keller^4^, Volker Auwärter^1,2*^

^1^ Institute of Forensic Medicine, Forensic Toxicology, Medical Center – University of Freiburg, Germany

^2^ Medical Faculty, University of Freiburg, Germany

^3^ Hermann Staudinger Graduate School, University of Freiburg, Germany

^4^ Institute of Forensic Medicine, Forensic Toxicology, University of Salzburg, Austria

**Supplementary material**

1. **QTOF MS/MS Mass Inclusion List**

The following masses were part of the MS/MS inclusion list to ensure acquisition in Auto MS/MS mode:

**Table S1 Ion inclusion list for analysis of the pHLM assay by QTOF.**

| ***m/z*** | **Description** |
| --- | --- |
| 193.0779 | Artefact |
| 210.1044 | Primary Amine (E4) |
| 224.0836 | Primary Amine - cyclohexanone (E9) |
| 226.0993 | Primary Amine - hydroxylated (E8) |
| 240.0786 | Baeyer-Villiger lacton (E10) |
| 244.2059 | Phencyclidine (PCP) |
| 260.2009 | 3-OH-PCP |
| 268.1460 | Artefact |
| 276.1514 | Dehydro-3-Cl-PCP |
| 278.1670 | 3-Cl-PCP |
| 282.1255 | Carboxy metabolite β‑oxidation (E5) |
| 292.1463 | Monooxygenated and dehydrated metabolite (G1) |
| 294.1619 | Monooxygenated metabolite |
| 296.1776 | Primary alcohol metabolite |
| 308.1411 | Dioxygenated and dehydrated metabolite |
| 310.1568 | Dioxygenated metabolite |
| 324.1361 | Trioxygenated and dehydrated metabolite |
| 326.1517 | Trioxygenated metabolite |

1. **Fragment ions in high-resolution MS/MS spectra and corresponding postulated structure**

**Table S2 List of fragment ions with ID and postulated structure in high-resolution MS/MS spectra**

| **ID** | ***m/z*** | **Postulated structure** |
| --- | --- | --- |
| F1 | 69.06988 |  |
| F2 | 79.05423 |  |
| F3 | 81.06988 |  |
| F4 | 84.08078 |  |
| F5 | 86.09643 |  |
| F6 | 101.05971 |  |
| F7 | 102.09134 |  |
| F8 | 104.10699 |  |
| F9 | 118.08626 |  |
| F10 | 125.01525 |  |
| F11 | 151.03090 |  |
| F12 | 163.03090 |  |
| **ID** | ***m/z*** | **Postulated structure** |
| F13 | 191.06220 |  |
| F14 | 193.07786 |  |

1. **LC-MS/MS MRM method parameter**
   1. ***in vivo* metabolite screening analysis**

Initially, a screening analysis was conducted employing multiple collision energies and encompassing a wide range of potential *in vivo* metabolites. The MRM transitions monitored are summarized in the table below. The cycle time was set to 1.5 s, with dwell times varying between 3 ms and 250 ms.

**Table S3 Broad-spectrum screening MRM method for detecting in vivo metabolites of 3‑Cl‑PCP (DP, declustering potential; EP, entrance potential; CE, collision energy; CXP, cell exit potential)**

| **Q_1_** | **Q_3_** | **RT** | **Analyte ID** | **DP** | **EP** | **CE** | **CXP** |
| --- | --- | --- | --- | --- | --- | --- | --- |
| 278.201 | 86.2 | 15.47 | 3-Cl-PCP 1 | 40 | 10 | 18 | 18 |
| 278.201 | 193.2 | 15.47 | 3-Cl-PCP 2 | 40 | 10 | 21 | 21 |
| 310.201 | 193.1 | 0 | 3-Cl-PCP-Carboxyl 1 | 130 | 10 | 15 | 15 |
| 310.201 | 125 | 0 | 3-Cl-PCP-Carboxyl 2 | 130 | 10 | 15 | 15 |
| 310.201 | 118.1 | 0 | 3-Cl-PCP-Carboxyl 3 | 130 | 10 | 15 | 15 |
| 310.201 | 101.1 | 0 | 3-Cl-PCP-Carboxyl 4 | 130 | 10 | 15 | 15 |
| 310.201 | 81.1 | 0 | 3-Cl-PCP-Carboxyl 5 | 130 | 10 | 15 | 15 |
| 310.201 | 193.101 | 0 | 3-Cl-PCP-Carboxyl_25eV 1 | 130 | 10 | 25 | 25 |
| 310.201 | 125.001 | 0 | 3-Cl-PCP-Carboxyl_25eV 2 | 130 | 10 | 25 | 25 |
| 310.201 | 118.101 | 0 | 3-Cl-PCP-Carboxyl_25eV 3 | 130 | 10 | 25 | 25 |
| 310.201 | 101.101 | 0 | 3-Cl-PCP-Carboxyl_25eV 4 | 130 | 10 | 25 | 25 |
| 310.201 | 81.101 | 0 | 3-Cl-PCP-Carboxyl_25eV 5 | 130 | 10 | 25 | 25 |
| 310.201 | 193.102 | 0 | 3-Cl-PCP-Carboxyl_40eV 1 | 130 | 10 | 40 | 40 |
| 310.201 | 125.002 | 0 | 3-Cl-PCP-Carboxyl_40eV 2 | 130 | 10 | 40 | 40 |
| **Q_1_** | **Q_3_** | **RT** | **Analyte ID** | **DP** | **EP** | **CE** | **CXP** |
| 310.201 | 118.102 | 0 | 3-Cl-PCP-Carboxyl_40eV 3 | 130 | 10 | 40 | 40 |
| 310.201 | 101.102 | 0 | 3-Cl-PCP-Carboxyl_40eV 4 | 130 | 10 | 40 | 40 |
| 310.201 | 81.102 | 0 | 3-Cl-PCP-Carboxyl_40eV 5 | 130 | 10 | 40 | 40 |
| 294.2 | 191.1 | 0 | 3-Cl-PCP-Cyclohexyl-OH 1 | 130 | 10 | 15 | 15 |
| 294.2 | 163 | 0 | 3-Cl-PCP-Cyclohexyl-OH 2 | 130 | 10 | 15 | 15 |
| 294.2 | 125 | 0 | 3-Cl-PCP-Cyclohexyl-OH 3 | 130 | 10 | 15 | 15 |
| 294.2 | 86.1 | 0 | 3-Cl-PCP-Cyclohexyl-OH 4 | 130 | 10 | 15 | 15 |
| 294.2 | 191.101 | 0 | 3-Cl-PCP-Cyclohexyl-OH_25eV 1 | 130 | 10 | 25 | 25 |
| 294.2 | 163.001 | 0 | 3-Cl-PCP-Cyclohexyl-OH_25eV 2 | 130 | 10 | 25 | 25 |
| 294.2 | 125.001 | 0 | 3-Cl-PCP-Cyclohexyl-OH_25eV 3 | 130 | 10 | 25 | 25 |
| 294.2 | 86.101 | 0 | 3-Cl-PCP-Cyclohexyl-OH_25eV 4 | 130 | 10 | 25 | 25 |
| 294.2 | 191.102 | 0 | 3-Cl-PCP-Cyclohexyl-OH_40eV 1 | 130 | 10 | 40 | 40 |
| 294.2 | 163.002 | 0 | 3-Cl-PCP-Cyclohexyl-OH_40eV 2 | 130 | 10 | 40 | 40 |
| 294.2 | 125.002 | 0 | 3-Cl-PCP-Cyclohexyl-OH_40eV 3 | 130 | 10 | 40 | 40 |
| 294.2 | 86.102 | 0 | 3-Cl-PCP-Cyclohexyl-OH_40eV 4 | 130 | 10 | 40 | 40 |
| 276.2 | 193.1 | 0 | 3-Cl-PCP-Dehydropiperidin 1 | 130 | 10 | 15 | 15 |
| 276.2 | 125 | 0 | 3-Cl-PCP-Dehydropiperidin 2 | 130 | 10 | 15 | 15 |
| 276.2 | 84.1 | 0 | 3-Cl-PCP-Dehydropiperidin 3 | 130 | 10 | 15 | 15 |
| 276.2 | 81.1 | 0 | 3-Cl-PCP-Dehydropiperidin 4 | 130 | 10 | 15 | 15 |
| 276.2 | 193.101 | 0 | 3-Cl-PCP-Dehydropiperidin_25eV 1 | 130 | 10 | 25 | 25 |
| 276.2 | 125.001 | 0 | 3-Cl-PCP-Dehydropiperidin_25eV 2 | 130 | 10 | 25 | 25 |
| 276.2 | 84.101 | 0 | 3-Cl-PCP-Dehydropiperidin_25eV 3 | 130 | 10 | 25 | 25 |
| 276.2 | 81.101 | 0 | 3-Cl-PCP-Dehydropiperidin_25eV 4 | 130 | 10 | 25 | 25 |
| 276.2 | 193.102 | 0 | 3-Cl-PCP-Dehydropiperidin_40eV 1 | 130 | 10 | 40 | 40 |
| 276.2 | 125.002 | 0 | 3-Cl-PCP-Dehydropiperidin_40eV 2 | 130 | 10 | 40 | 40 |
| 276.2 | 84.102 | 0 | 3-Cl-PCP-Dehydropiperidin_40eV 3 | 130 | 10 | 40 | 40 |
| 276.2 | 81.102 | 0 | 3-Cl-PCP-Dehydropiperidin_40eV 4 | 130 | 10 | 40 | 40 |
| 326.2 | 134.1 | 0 | 3-Cl-PCP-Hydroxycarboxyl 1 | 130 | 10 | 15 | 15 |
| 326.2 | 134.101 | 0 | 3-Cl-PCP-Hydroxycarboxyl_25eV 1 | 130 | 10 | 25 | 25 |
| 326.2 | 134.102 | 0 | 3-Cl-PCP-Hydroxycarboxyl_40eV 1 | 130 | 10 | 40 | 40 |
| **Q_1_** | **Q_3_** | **RT** | **Analyte ID** | **DP** | **EP** | **CE** | **CXP** |
| 326.2 | 125 | 0 | 3-Cl-PCP-Hydroxycarboxyl 2 | 130 | 10 | 15 | 15 |
| 326.2 | 116.1 | 0 | 3-Cl-PCP-Hydroxycarboxyl 3 | 130 | 10 | 15 | 15 |
| 326.2 | 81.1 | 0 | 3-Cl-PCP-Hydroxycarboxyl 4 | 130 | 10 | 15 | 15 |
| 326.2 | 125.001 | 0 | 3-Cl-PCP-Hydroxycarboxyl_25eV 2 | 130 | 10 | 25 | 25 |
| 326.2 | 116.101 | 0 | 3-Cl-PCP-Hydroxycarboxyl_25eV 3 | 130 | 10 | 25 | 25 |
| 326.2 | 81.101 | 0 | 3-Cl-PCP-Hydroxycarboxyl_25eV 4 | 130 | 10 | 25 | 25 |
| 326.2 | 125.002 | 0 | 3-Cl-PCP-Hydroxycarboxyl_40eV 2 | 130 | 10 | 40 | 40 |
| 326.2 | 116.102 | 0 | 3-Cl-PCP-Hydroxycarboxyl_40eV 3 | 130 | 10 | 40 | 40 |
| 326.2 | 81.102 | 0 | 3-Cl-PCP-Hydroxycarboxyl_40eV 4 | 130 | 10 | 40 | 40 |
| 210.1 | 125 | 0 | 3-Cl-PCP-N-Desalkylierung-mitN 1 | 130 | 10 | 15 | 15 |
| 210.1 | 193.1 | 0 | 3-Cl-PCP-N-Desalkylierung-mitN 2 | 130 | 10 | 15 | 15 |
| 210.1 | 125.001 | 0 | 3-Cl-PCP-N-Desalkylierung-mitN_25eV 1 | 130 | 10 | 25 | 25 |
| 210.1 | 193.101 | 0 | 3-Cl-PCP-N-Desalkylierung-mitN_25eV 2 | 130 | 10 | 25 | 25 |
| 210.1 | 125.002 | 0 | 3-Cl-PCP-N-Desalkylierung-mitN_40eV 1 | 130 | 10 | 40 | 40 |
| 210.1 | 193.102 | 0 | 3-Cl-PCP-N-Desalkylierung-mitN_40eV 2 | 130 | 10 | 40 | 40 |
| 193.1 | 125 | 0 | 3-Cl-PCP-N-Desalkylierung-ohneN 1 | 130 | 10 | 15 | 15 |
| 193.1 | 81.1 | 0 | 3-Cl-PCP-N-Desalkylierung-ohneN 2 | 130 | 10 | 15 | 15 |
| 193.1 | 125.001 | 0 | 3-Cl-PCP-N-Desalkylierung-ohneN_25eV 1 | 130 | 10 | 25 | 25 |
| 193.1 | 81.101 | 0 | 3-Cl-PCP-N-Desalkylierung-ohneN_25eV 2 | 130 | 10 | 25 | 25 |
| 193.1 | 125.002 | 0 | 3-Cl-PCP-N-Desalkylierung-ohneN_40eV 1 | 130 | 10 | 40 | 40 |
| 193.1 | 81.102 | 0 | 3-Cl-PCP-N-Desalkylierung-ohneN_40eV 2 | 130 | 10 | 40 | 40 |
| 310.2 | 193.1 | 0 | 3-Cl-PCP-Piperidin-DiOH 1 | 130 | 10 | 15 | 15 |
| 310.2 | 125 | 0 | 3-Cl-PCP-Piperidin-DiOH 2 | 130 | 10 | 15 | 15 |
| 310.2 | 80 | 0 | 3-Cl-PCP-Piperidin-DiOH 3 | 130 | 10 | 15 | 15 |
| 310.2 | 100.1 | 0 | 3-Cl-PCP-Piperidin-DiOH 4 | 130 | 10 | 15 | 15 |
| **Q_1_** | **Q_3_** | **RT** | **Analyte ID** | **DP** | **EP** | **CE** | **CXP** |
| 310.2 | 81.1 | 0 | 3-Cl-PCP-Piperidin-DiOH 5 | 130 | 10 | 15 | 15 |
| 310.2 | 193.101 | 0 | 3-Cl-PCP-Piperidin-DiOH_25eV 1 | 130 | 10 | 25 | 25 |
| 310.2 | 125.001 | 0 | 3-Cl-PCP-Piperidin-DiOH_25eV 2 | 130 | 10 | 25 | 25 |
| 310.2 | 80.001 | 0 | 3-Cl-PCP-Piperidin-DiOH_25eV 3 | 130 | 10 | 25 | 25 |
| 310.2 | 100.101 | 0 | 3-Cl-PCP-Piperidin-DiOH_25eV 4 | 130 | 10 | 25 | 25 |
| 310.2 | 81.101 | 0 | 3-Cl-PCP-Piperidin-DiOH_25eV 5 | 130 | 10 | 25 | 25 |
| 310.2 | 193.102 | 0 | 3-Cl-PCP-Piperidin-DiOH_40eV 1 | 130 | 10 | 40 | 40 |
| 310.2 | 125.002 | 0 | 3-Cl-PCP-Piperidin-DiOH_40eV 2 | 130 | 10 | 40 | 40 |
| 310.2 | 80.002 | 0 | 3-Cl-PCP-Piperidin-DiOH_40eV 3 | 130 | 10 | 40 | 40 |
| 310.2 | 100.102 | 0 | 3-Cl-PCP-Piperidin-DiOH_40eV 4 | 130 | 10 | 40 | 40 |
| 310.2 | 81.102 | 0 | 3-Cl-PCP-Piperidin-DiOH_40eV 5 | 130 | 10 | 40 | 40 |
| 294.201 | 193.1 | 0 | 3-Cl-PCP-Piperidin-OH 1 | 130 | 10 | 15 | 15 |
| 294.201 | 125 | 0 | 3-Cl-PCP-Piperidin-OH 2 | 130 | 10 | 15 | 15 |
| 294.201 | 102.1 | 0 | 3-Cl-PCP-Piperidin-OH 3 | 130 | 10 | 15 | 15 |
| 294.201 | 84.1 | 0 | 3-Cl-PCP-Piperidin-OH 4 | 130 | 10 | 15 | 15 |
| 294.201 | 193.101 | 0 | 3-Cl-PCP-Piperidin-OH_25eV 1 | 130 | 10 | 25 | 25 |
| 294.201 | 125.001 | 0 | 3-Cl-PCP-Piperidin-OH_25eV 2 | 130 | 10 | 25 | 25 |
| 294.201 | 102.101 | 0 | 3-Cl-PCP-Piperidin-OH_25eV 3 | 130 | 10 | 25 | 25 |
| 294.201 | 84.101 | 0 | 3-Cl-PCP-Piperidin-OH_25eV 4 | 130 | 10 | 25 | 25 |
| 294.201 | 193.102 | 0 | 3-Cl-PCP-Piperidin-OH_40eV 1 | 130 | 10 | 40 | 40 |
| 294.201 | 125.002 | 0 | 3-Cl-PCP-Piperidin-OH_40eV 2 | 130 | 10 | 40 | 40 |
| 294.201 | 102.102 | 0 | 3-Cl-PCP-Piperidin-OH_40eV 3 | 130 | 10 | 40 | 40 |
| 294.201 | 84.102 | 0 | 3-Cl-PCP-Piperidin-OH_40eV 4 | 130 | 10 | 40 | 40 |
| 296.2 | 125 | 0 | 3-Cl-PCP-primaerer-Alkohol 1 | 130 | 10 | 15 | 15 |
| 296.2 | 104.1 | 0 | 3-Cl-PCP-primaerer-Alkohol 2 | 130 | 10 | 15 | 15 |
| 296.2 | 69.1 | 0 | 3-Cl-PCP-primaerer-Alkohol 3 | 130 | 10 | 15 | 15 |
| 296.2 | 125.001 | 0 | 3-Cl-PCP-primaerer-Alkohol_25eV 1 | 130 | 10 | 25 | 25 |
| 296.2 | 104.101 | 0 | 3-Cl-PCP-primaerer-Alkohol_25eV 2 | 130 | 10 | 25 | 25 |
| 296.2 | 69.101 | 0 | 3-Cl-PCP-primaerer-Alkohol_25eV 3 | 130 | 10 | 25 | 25 |
| 296.2 | 125.002 | 0 | 3-Cl-PCP-primaerer-Alkohol_40eV 1 | 130 | 10 | 40 | 40 |
| **Q_1_** | **Q_3_** | **RT** | **Analyte ID** | **DP** | **EP** | **CE** | **CXP** |
| 296.2 | 104.102 | 0 | 3-Cl-PCP-primaerer-Alkohol_40eV 2 | 130 | 10 | 40 | 40 |
| 296.2 | 69.102 | 0 | 3-Cl-PCP-primaerer-Alkohol_40eV 3 | 130 | 10 | 25 | 25 |
| 244.1 | 86 | 14.88 | PCP 1 | 50 | 6 | 18 | 18 |
| 244.1 | 159.1 | 14.88 | PCP 2 | 50 | 6 | 21 | 21 |
| 249.1 | 164.1 | 14.81 | d5‑PCP | 50 | 6 | 20 | 20 |

- 1. ***in vivo* method for peak area determination**

Following data evaluation, an additional MRM method was developed, comprising only two to three transitions per detected metabolite. Furthermore, a carboxyl metabolite with an n–2 shorter carbon chain was included, as this compound is not expected to be formed in the pHLM assay but may arise under *in vivo* conditions. PCP was not detected.

**Table S4 Optimized MRM method for the determination of peak areas of detected in vivo metabolites (DP, declustering potential; EP, entrance potential; CE, collision energy; CXP, cell exit potential)**

| **Q1** | **Q3** | **RT** | **Analyte ID** | **DP** | **EP** | **CE** | **CXP** |
| --- | --- | --- | --- | --- | --- | --- | --- |
| 244.1 | 86 | 14.88 | PCP 1 | 50 | 6 | 18 | 14 |
| 244.1 | 159.1 | 14.88 | PCP 2 | 50 | 6 | 21 | 11 |
| 249.1 | 164.1 | 14.81 | d5-PCP | 50 | 6 | 20 | 14 |
| 278.2 | 86.2 | 15.47 | 3-Cl-PCP 1 | 40 | 10 | 18 | 10 |
| 278.2 | 193.2 | 15.47 | 3-Cl-PCP 2 | 40 | 10 | 21 | 10 |
| 310.2 | 193.1 | 0 | 3-Cl-PCP-Carboxyl-1 | 130 | 10 | 25 | 13 |
| 310.2 | 118.1 | 0 | 3-Cl-PCP-Carboxyl-2 | 130 | 10 | 15 | 13 |
| 294.2 | 125 | 0 | 3-Cl-PCP-OH | 130 | 10 | 40 | 13 |
| 294.2 | 163 | 0 | 3-Cl-PCP-Cyclohexyl-OH-1 | 130 | 10 | 40 | 13 |
| 294.2 | 86.1 | 0 | 3-Cl-PCP-Cyclohexyl-OH-2 | 130 | 10 | 15 | 13 |
| 294.2 | 193.1 | 0 | 3-Cl-PCP-Piperidin-OH-1 | 130 | 10 | 25 | 13 |
| 294.2 | 102.1 | 0 | 3-Cl-PCP-Piperidin-OH-2 | 130 | 10 | 15 | 13 |
| 296.2 | 125 | 0 | 3-Cl-PCP-primary alcohol-1 | 130 | 10 | 40 | 13 |
| 296.2 | 104.1 | 0 | 3-Cl-PCP-primary alcohol-2 | 130 | 10 | 15 | 13 |
| 282.1 | 193.1 | 0 | 3-Cl-PCP-BetaCarboxyl-1 | 130 | 10 | 25 | 13 |
| 282.1 | 90.1 | 0 | 3-Cl-PCP-BetaCarboxyl-2 | 130 | 10 | 15 | 13 |

1. **LC-MS/MS peak areas of metabolites in post mortem matrices**

**Table S5 Peak areas obtained from MS/MS analysis of the respective matrix (cardiac blood, bile and urine). The acceptance criteria were established in accordance with GTFCh (German Society of Toxicology & Forensic Chemistry) guidelines, using pooled human liver microsomes (pHLM) metabolites as reference. Peak areas highlighted in yellow fall outside the predefined acceptance range.**

|  |  | **pHLM** | **Qual/Quant** | **Cardiac blood** | **Qual/Quant** | **Acceptance criteria** | |
| --- | --- | --- | --- | --- | --- | --- | --- |
|  | PCP-D5 | - |  | 2.40E+07 |  | min | max |
| M1 - CyclohexylOH | 294.2-163.0 | 4.95E+03 | 0.06 | - |  | 0.03 | 0.09 |
|  | 294.2-125.0 | 2.85E+04 | 0.35 | - |  | 0.26 | 0.43 |
| Quantifier | 294.2-86.1 | 8.25E+04 | 1.00 | - |  |  |  |
| Response Quant/IS |  |  |  | - |  |  |  |
| M2- CyclohexylOH | 294.2-163.0 | 1.66E+04 | 0.27 | 8.20E+03 | 0.21 | 0.20 | 0.34 |
|  | 294.2-125.0 | 6.16E+04 | 1.00 | 3.98E+04 | 1.04 | 0.80 | 1.20 |
| Quantifier | 294.2-86.1 | 6.17E+04 | 1.00 | 3.84E+04 | 1.00 |  |  |
| Response Quant/IS |  |  |  | 1.60E-03 |  |  |  |
| M3 - CyclohexylOH | 294.2-163.0 | 8.66E+04 | 0.10 | 6.61E+03 | 0.10 | 0.05 | 0.16 |
|  | 294.2-125.0 | 3.79E+05 | 0.46 | 3.00E+04 | 0.47 | 0.34 | 0.57 |
| Quantifier | 294.2-86.1 | 8.30E+05 | 1.00 | 6.36E+04 | 1.00 |  |  |
| Response Quant/IS |  |  |  | 2.65E-03 |  |  |  |
| M4 - PiperidinOH | 294.2-193.2 | 2.24E+05 | 0.56 | 1.41E+05 | 0.57 | 0.45 | 0.67 |
|  | 294.2-125.0 | 2.75E+05 | 0.69 | 1.70E+05 | 0.69 | 0.55 | 0.83 |
| Quantifier | 294.2-102.1 | 3.98E+05 | 1.00 | 2.47E+05 | 1.00 |  |  |
| Response Quant/IS |  |  |  | 1.03E-02 |  |  |  |
| M5 - PiperidinOH | 294.2-193.2 | 8.92E+04 | 0.57 | 1.61E+04 | 0.50 | 0.46 | 0.69 |
|  | 294.2-125.0 | 1.11E+05 | 0.72 | 2.31E+04 | 0.71 | 0.57 | 0.86 |
| Quantifier | 294.2-102.1 | 1.55E+05 | 1.00 | 3.24E+04 | 1.00 |  |  |
| Response Quant/IS |  |  |  | 1.35E-03 |  |  |  |
| M6 - Carboxyl | 310.2-193.1 | 1.44E+05 | 0.77 | 3.49E+05 | 0.76 | 0.62 | 0.92 |
| Quantifier | 310.2-118.1 | 1.86E+05 | 1.00 | 4.58E+05 | 1.00 |  |  |
| Response Quant/IS |  |  |  | 1.91E-02 |  |  |  |
| M7 – primaryOH | 296.2-125.0 | 6.96E+04 | 0.88 | 8.01E+03 | 0.86 | 0.70 | 1.05 |
| Quantifier | 296.2-104.1 | 7.95E+04 | 1.00 | 9.31E+03 | 1.00 |  |  |
| Response Quant/IS |  |  |  | 3.88E-04 |  |  |  |

|  |  | **β-bile 1:10** | **Qual/Quant** | **Bile** | **Qual/Quant** | **Acceptance criteria** | |
| --- | --- | --- | --- | --- | --- | --- | --- |
|  | PCP-D5 | 1.43E+06 |  | 1.27E+07 |  | min | max |
| M1 - CyclohexylOH | 294.2-163.0 | - |  | 3.03E+03 | 0.07 | 0.03 | 0.09 |
|  | 294.2-125.0 | - |  | 1.79E+04 | 0.41 | 0.26 | 0.43 |
| Quantifier | 294.2-86.1 | - |  | 4.37E+04 | 1.00 |  |  |
| Response Quant/IS |  | - |  | 3.45E-03 |  |  |  |
| M2- CyclohexylOH | 294.2-163.0 | 4.78E+03 | 0.48 | 4.68E+04 | 0.64 | 0.20 | 0.34 |
|  | 294.2-125.0 | 1.62E+04 | 1.62 | 2.03E+05 | 2.77 | 0.80 | 1.20 |
| Quantifier | 294.2-86.1 | 1.00E+04 | 1.00 | 7.32E+04 | 1.00 |  |  |
| Response Quant/IS |  | 7.00E-03 |  | 5.78E-03 |  |  |  |
| M3 - CyclohexylOH | 294.2-163.0 | 7.97E+04 | 0.10 | 1.01E+05 | 0.10 | 0.05 | 0.16 |
|  | 294.2-125.0 | 3.71E+05 | 0.45 | 4.39E+05 | 0.45 | 0.34 | 0.57 |
| Quantifier | 294.2-86.1 | 8.30E+05 | 1.00 | 9.66E+05 | 1.00 |  |  |
| Response Quant/IS |  | 5.79E-01 |  | 7.63E-02 |  |  |  |
| M4 - PiperidinOH | 294.2-193.2 | 2.87E+05 | 0.60 | 5.96E+05 | 0.60 | 0.45 | 0.67 |
|  | 294.2-125.0 | 3.37E+05 | 0.70 | 7.10E+05 | 0.71 | 0.55 | 0.83 |
| Quantifier | 294.2-102.1 | 4.79E+05 | 1.00 | 9.96E+05 | 1.00 |  |  |
| Response Quant/IS |  | 3.34E-01 |  | 7.87E-02 |  |  |  |
| M5 - PiperidinOH | 294.2-193.2 | 1.24E+05 | 0.57 | 9.75E+04 | 0.59 | 0.46 | 0.69 |
|  | 294.2-125.0 | 1.49E+05 | 0.68 | 1.24E+05 | 0.76 | 0.57 | 0.86 |
| Quantifier | 294.2-102.1 | 2.17E+05 | 1.00 | 1.64E+05 | 1.00 |  |  |
| Response Quant/IS |  | 1.52E-01 |  | 1.30E-02 |  |  |  |
| M6 - Carboxyl | 310.2-193.1 | 1.03E+06 | 0.78 | 1.76E+07 | 0.75 | 0.62 | 0.92 |
| Quantifier | 310.2-118.1 | 1.32E+06 | 1.00 | 2.35E+07 | 1.00 |  |  |
| Response Quant/IS |  | 9.24E-01 |  | 1.85E+00 |  |  |  |
| M7 – primaryOH | 296.2-125.0 | 8.77E+04 | 0.79 | 1.57E+05 | 0.79 | 0.70 | 1.05 |
| Quantifier | 296.2-104.1 | 1.11E+05 | 1.00 | 1.98E+05 | 1.00 |  |  |
| Response Quant/IS |  | 7.72E-02 |  | 8.74E-03 |  |  |  |

|  |  | **β-Urine** | **Qual/Quant** | **Urine 1:10** | **Qual/Quant** | **Acceptance criteria** | |
| --- | --- | --- | --- | --- | --- | --- | --- |
|  | PCP-D5 | 1.45E+06 |  | 2.94E+07 |  | min | max |
| M1 - CyclohexylOH | 294.2-163.0 | 1.53E+05 | 0.07 | 4.80E+03 | 0.10 | 0.03 | 0.09 |
|  | 294.2-125.0 | 8.19E+05 | 0.35 | 1.98E+04 | 0.40 | 0.26 | 0.43 |
| Quantifier | 294.2-86.1 | 2.32E+06 | 1.00 | 4.89E+04 | 1.00 |  |  |
| Response Quant/IS |  | 1.60E+00 |  | 1.66E-03 |  |  |  |
| M2- CyclohexylOH | 294.2-163.0 | 2.05E+05 | 0.23 | 2.25E+04 | 0.26 | 0.20 | 0.34 |
|  | 294.2-125.0 | 8.98E+05 | 1.01 | 8.94E+04 | 1.04 | 0.80 | 1.20 |
| Quantifier | 294.2-86.1 | 8.91E+05 | 1.00 | 8.61E+04 | 1.00 |  |  |
| Response Quant/IS |  | 6.13E-01 |  | 2.92E-03 |  |  |  |
| M3 - CyclohexylOH | 294.2-163.0 | 4.10E+05 | 0.10 | 2.03E+04 | 0.15 | 0.05 | 0.16 |
|  | 294.2-125.0 | 1.86E+06 | 0.44 | 5.96E+04 | 0.45 | 0.34 | 0.57 |
| Quantifier | 294.2-86.1 | 4.21E+06 | 1.00 | 1.32E+05 | 1.00 |  |  |
| Response Quant/IS |  | 2.90E+00 |  | 4.49E-03 |  |  |  |
| M4 - PiperidinOH | 294.2-193.2 | 7.45E+06 | 0.59 | 2.53E+05 | 0.58 | 0.45 | 0.67 |
|  | 294.2-125.0 | 8.87E+06 | 0.70 | 2.99E+05 | 0.69 | 0.55 | 0.83 |
| Quantifier | 294.2-102.1 | 1.27E+07 | 1.00 | 4.33E+05 | 1.00 |  |  |
| Response Quant/IS |  | 8.76E+00 |  | 1.47E-02 |  |  |  |
| M5 - PiperidinOH | 294.2-193.2 | 6.18E+05 | 0.58 | 1.17E+04 | 0.59 | 0.46 | 0.69 |
|  | 294.2-125.0 | 7.58E+05 | 0.71 | 1.71E+04 | 0.86 | 0.57 | 0.86 |
| Quantifier | 294.2-102.1 | 1.07E+06 | 1.00 | 2.00E+04 | 1.00 |  |  |
| Response Quant/IS |  | 7.37E-01 |  | 6.79E-04 |  |  |  |
| M6 - Carboxyl | 310.2-193.1 | 3.86E+06 | 0.78 | 3.30E+06 | 0.77 | 0.62 | 0.92 |
| Quantifer | 310.2-118.1 | 4.93E+06 | 1.00 | 4.30E+06 | 1.00 |  |  |
| Response Quant/IS |  | 3.40E+00 |  | 1.46E-01 |  |  |  |
| M7 - primaryOH | 296.2-125.0 | 5.56E+05 | 0.83 | 3.39E+04 | 0.87 | 0.70 | 1.05 |
| Quantifier | 296.2-104.1 | 6.69E+05 | 1.00 | 3.88E+04 | 1.00 |  |  |
| Response Quant/IS |  | 4.61E-01 |  | 1.32E-03 |  |  |  |

| 1. **LC-MS/MS chromatograms of in vitro and in vivo metabolites**   **5.1 Hydroxycyclohexyl metabolites M1-3**  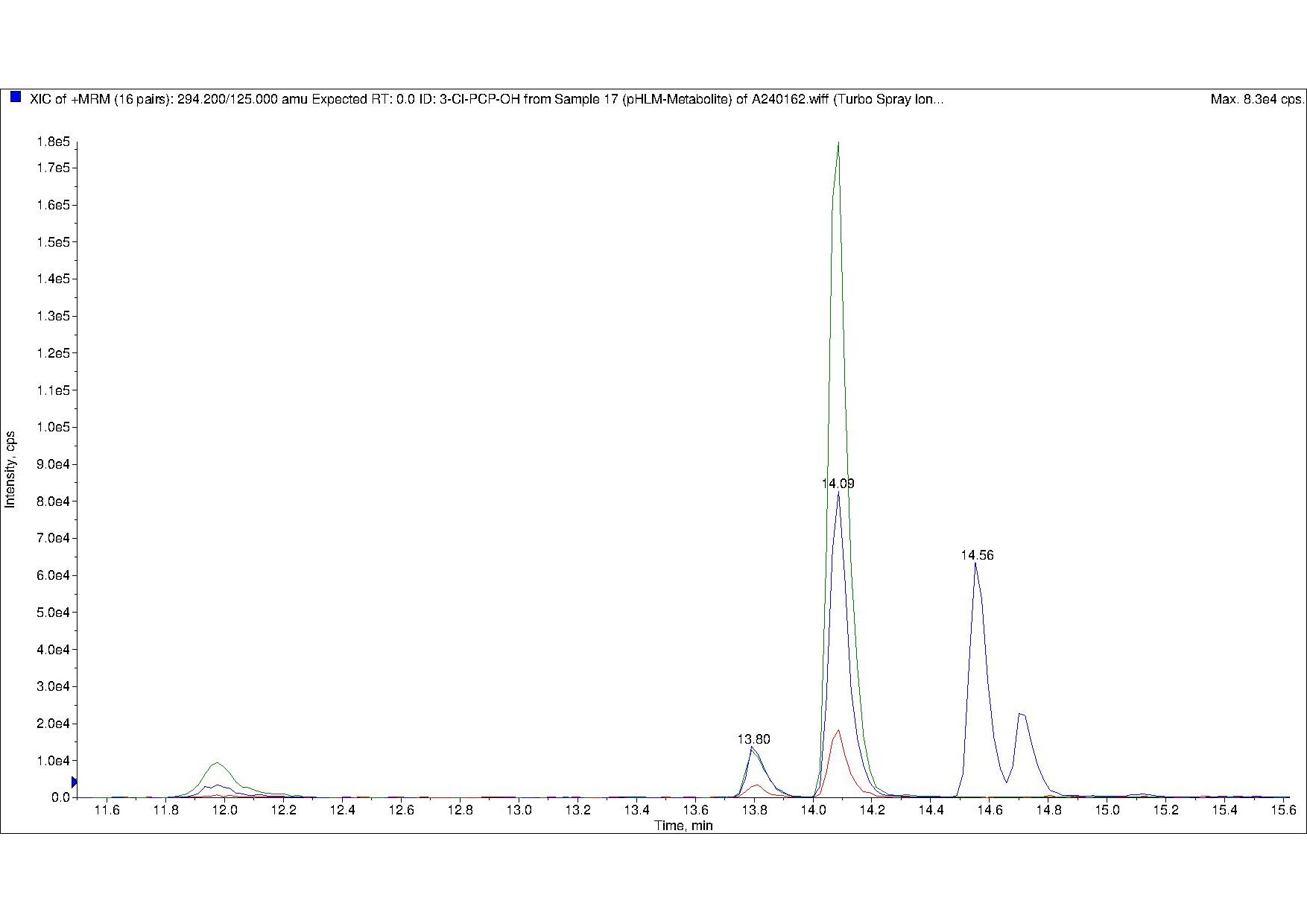  **Figure S1** Chromatogram of the three transitions of the hydroxycyclohexyl metabolites M1–3 from the pHLM incubation. (enlarged; blue: 294.2 → 125.0, red: 294.2 → 163.0, green: 294.2 → 86.1) |
| --- |
| 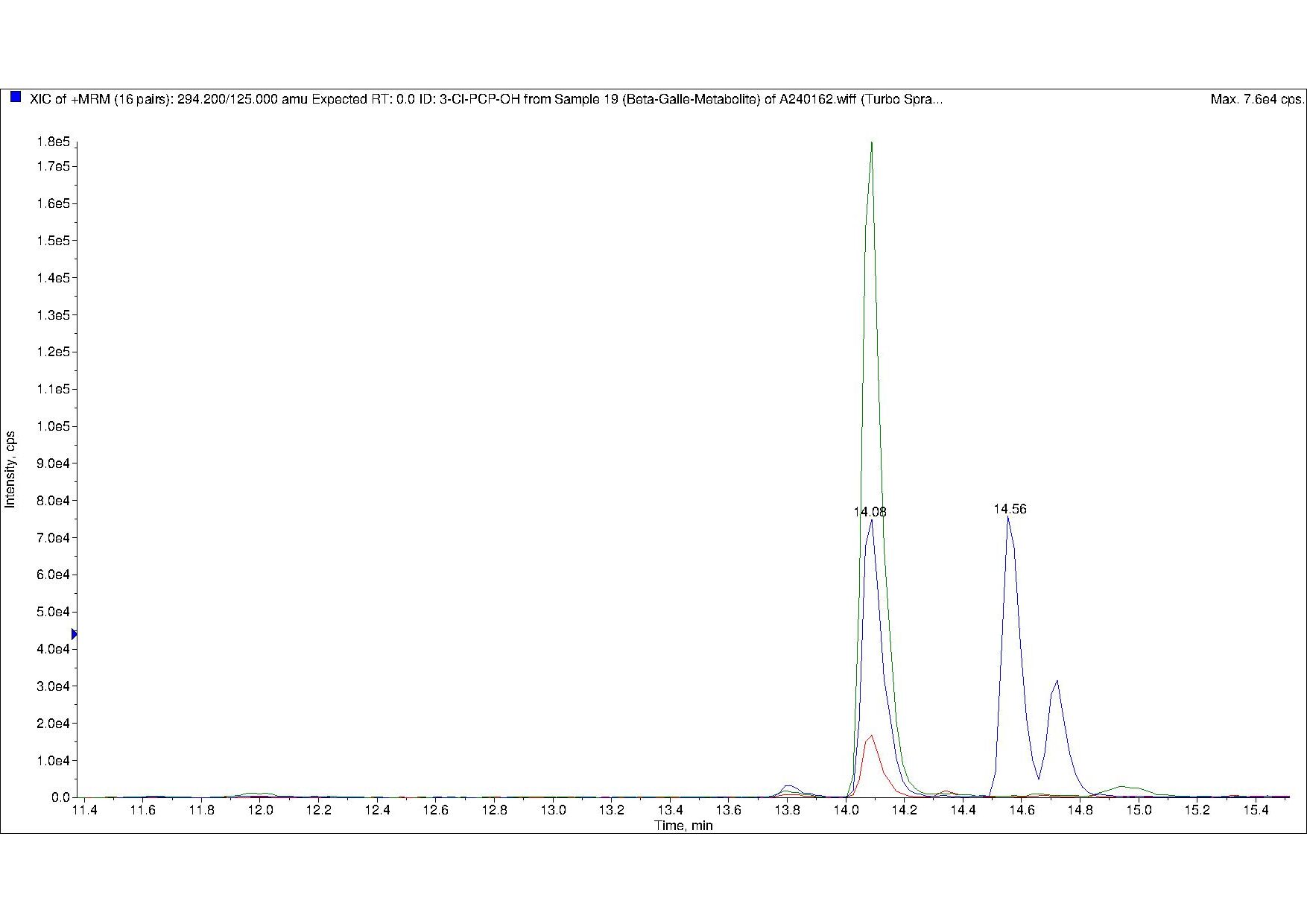  **Figure S2** Chromatogram of the three transitions of the hydroxycyclohexyl metabolites M1–3 in bile after β-glucuronidase hydrolysis (enlarged; blue: 294.2 → 125.0, red: 294.2 → 163.0, green: 294.2 → 86.1) |
| 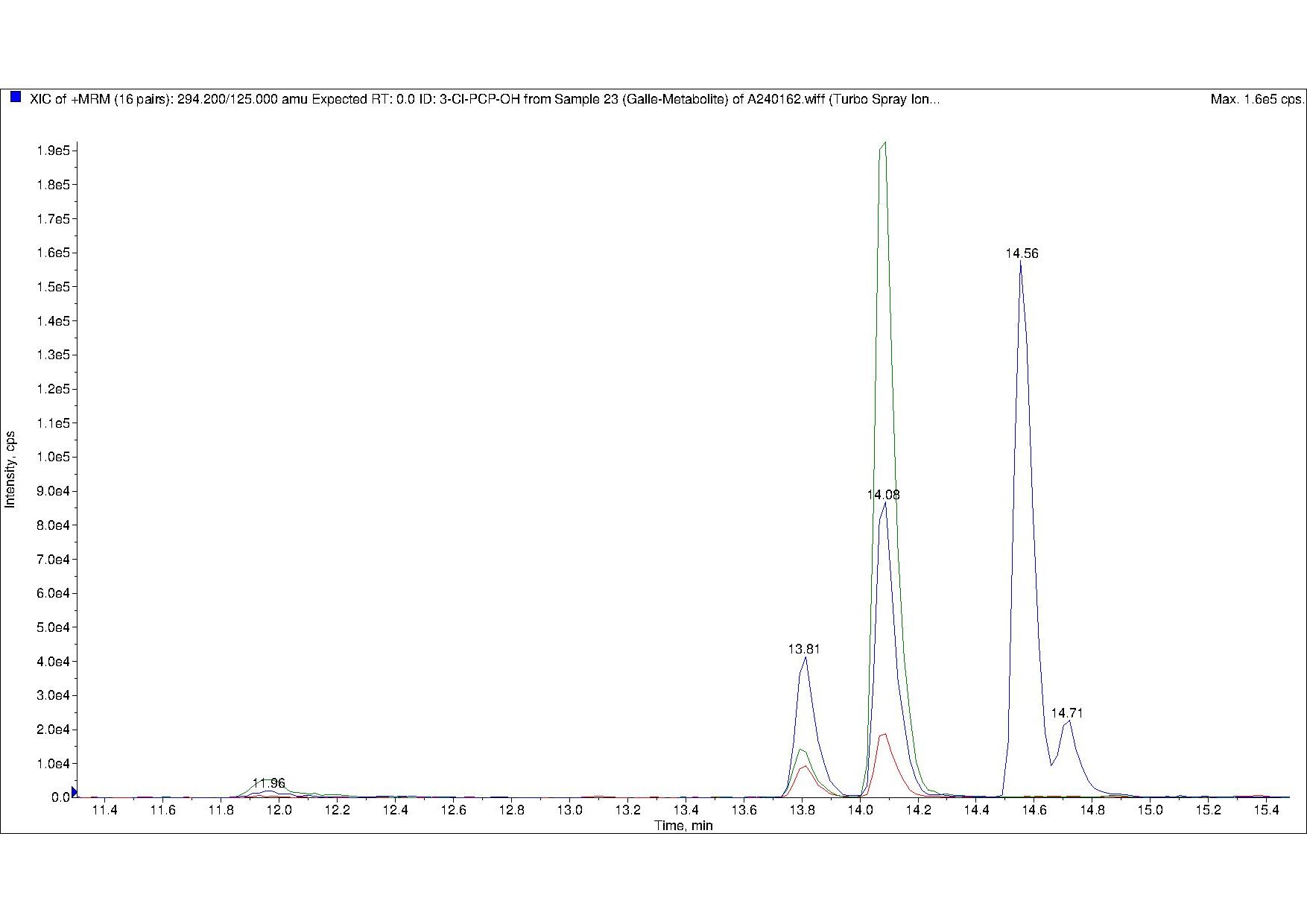  **Figure S3** Chromatogram of the three transitions of the hydroxycyclohexyl metabolites M1–3 in bile without β-glucuronidase hydrolysis (enlarged; blue: 294.2 → 125.0, red: 294.2 → 163.0, green: 294.2 → 86.1) |
| 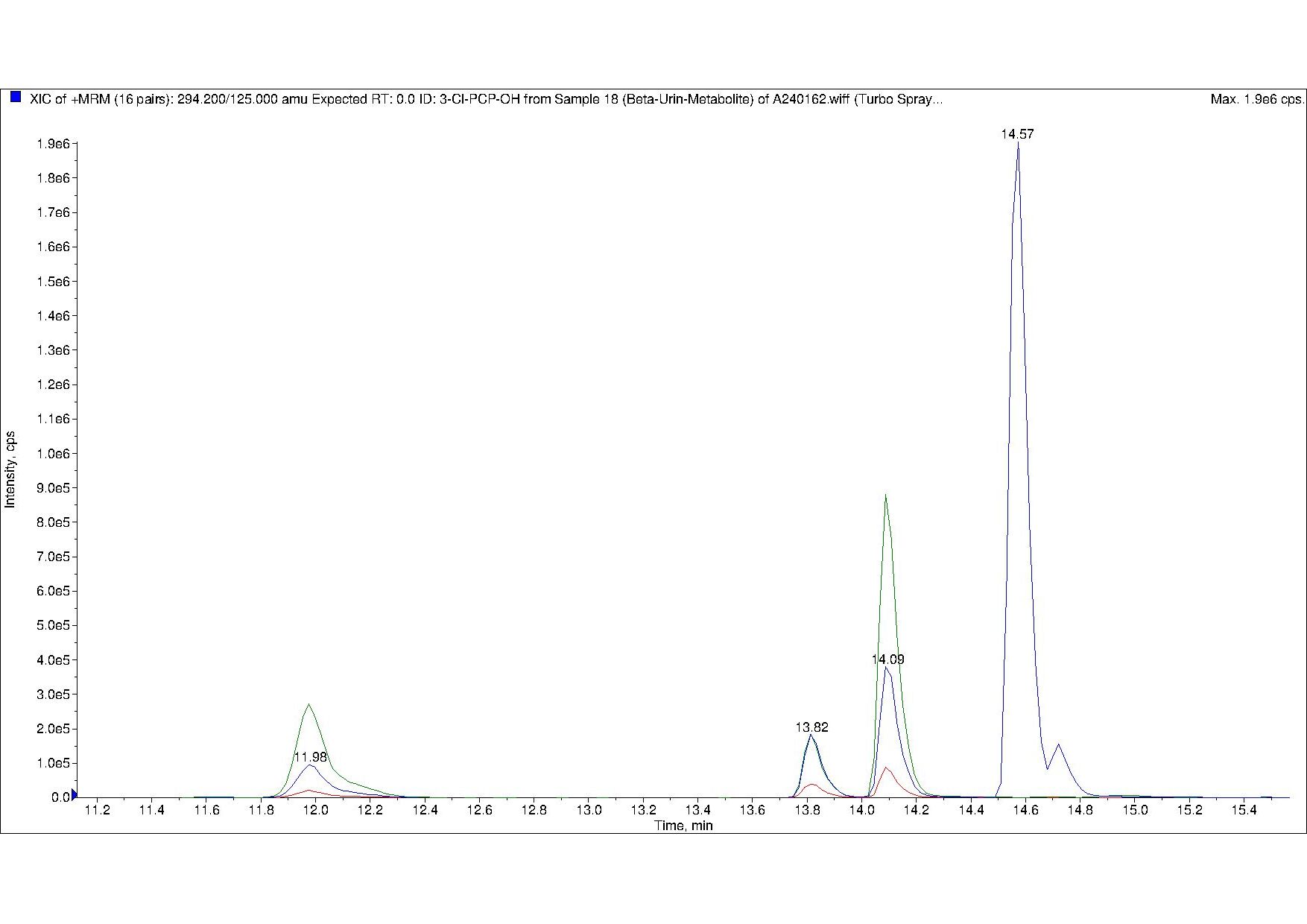  **Figure S4** Chromatogram of the three transitions of the hydroxycyclohexyl metabolites M1–3 in urine after β-glucuronidase hydrolysis (enlarged; blue: 294.2 → 125.0, red: 294.2 → 163.0, green: 294.2 → 86.1) |
| 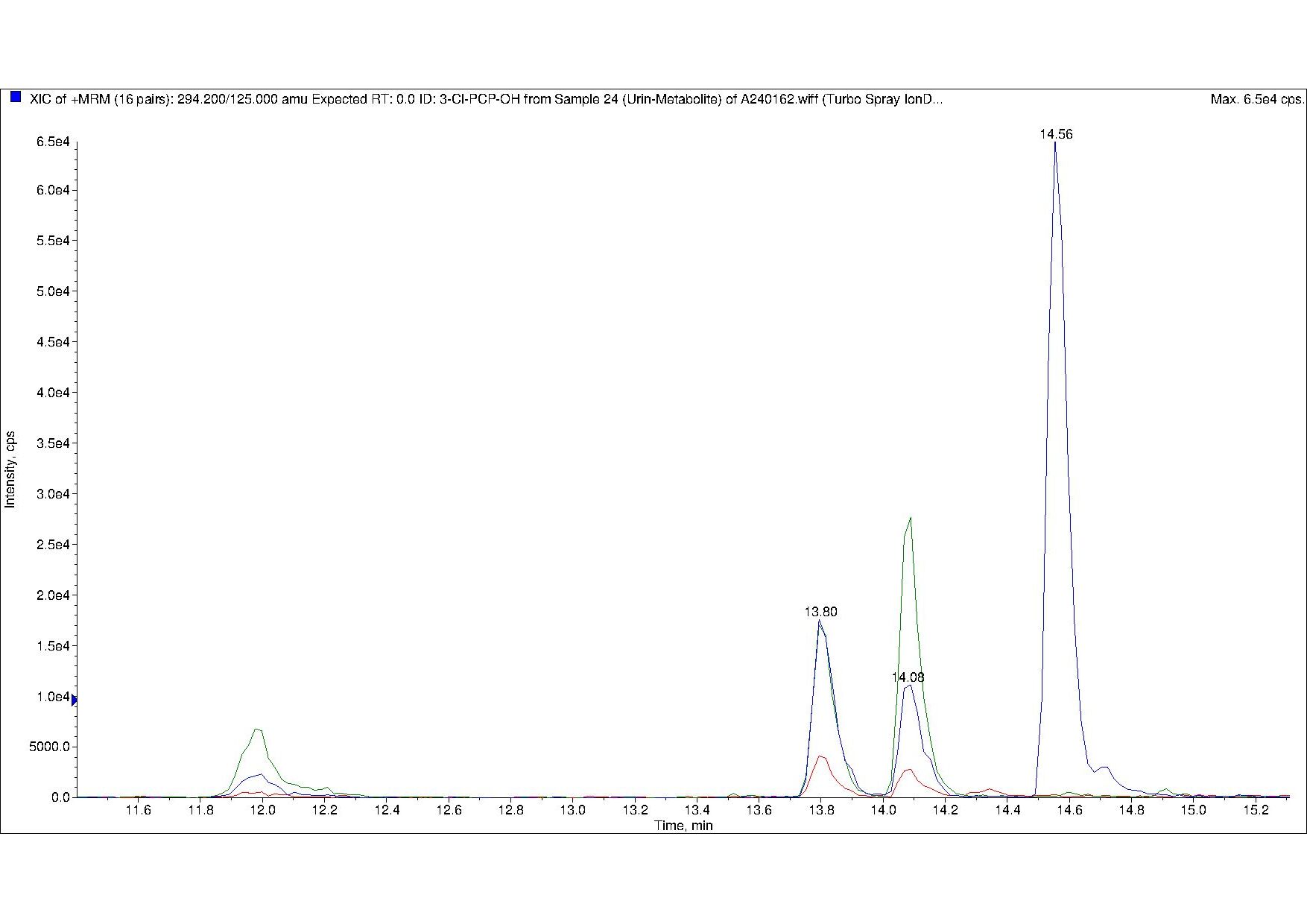  **Figure S5** Chromatogram of the three transitions of the hydroxycyclohexyl metabolites M1–3 in urine without β-glucuronidase hydrolysis (enlarged; blue: 294.2 → 125.0, red: 294.2 → 163.0, green: 294.2 → 86.1) |
| 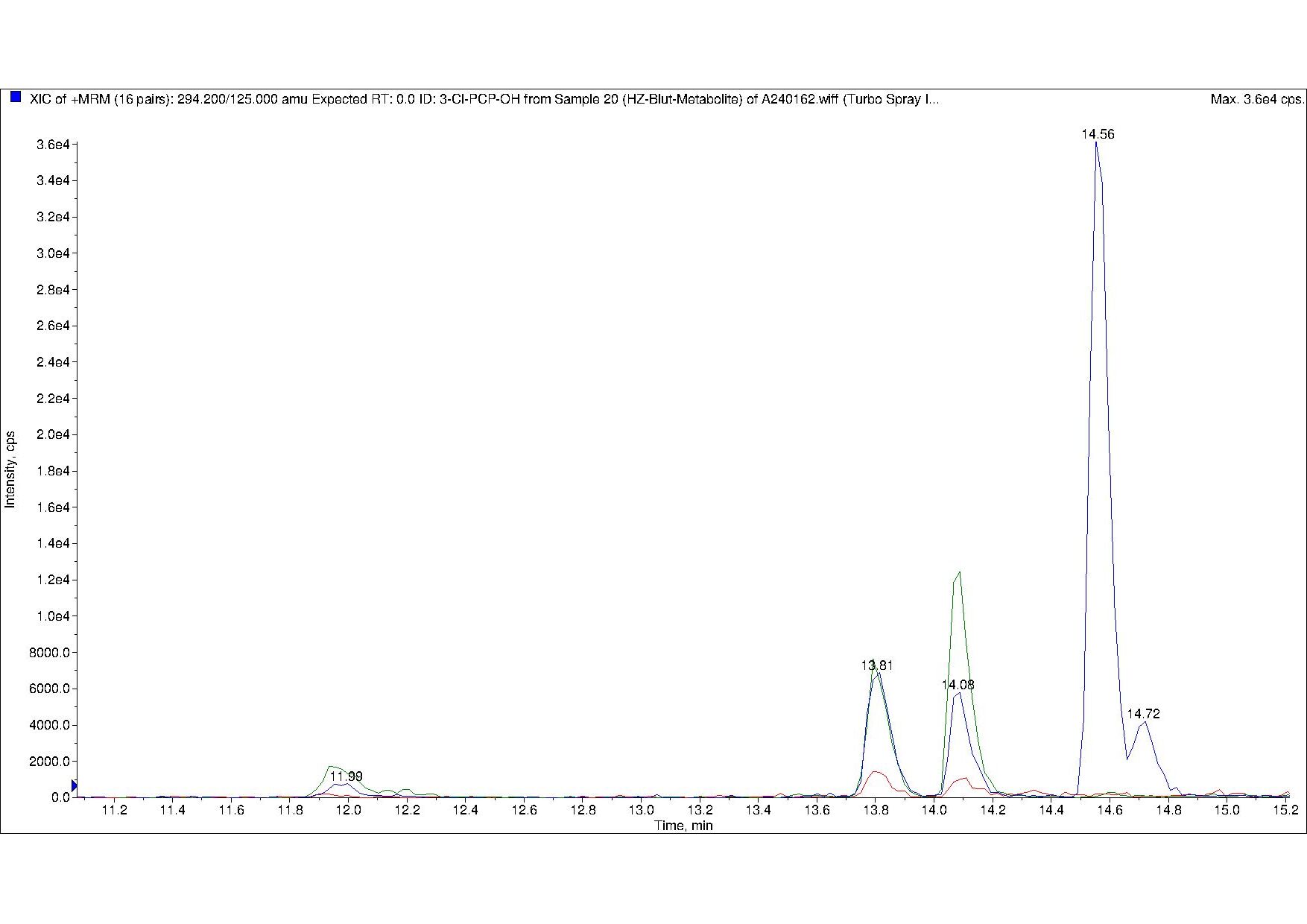  **Figure S6** Chromatogram of the three transitions of the hydroxycyclohexyl metabolites M1–3 in cardiac blood (enlarged; blue: 294.2 → 125.0, red: 294.2 → 163.0, green: 294.2 → 86.1) |
| 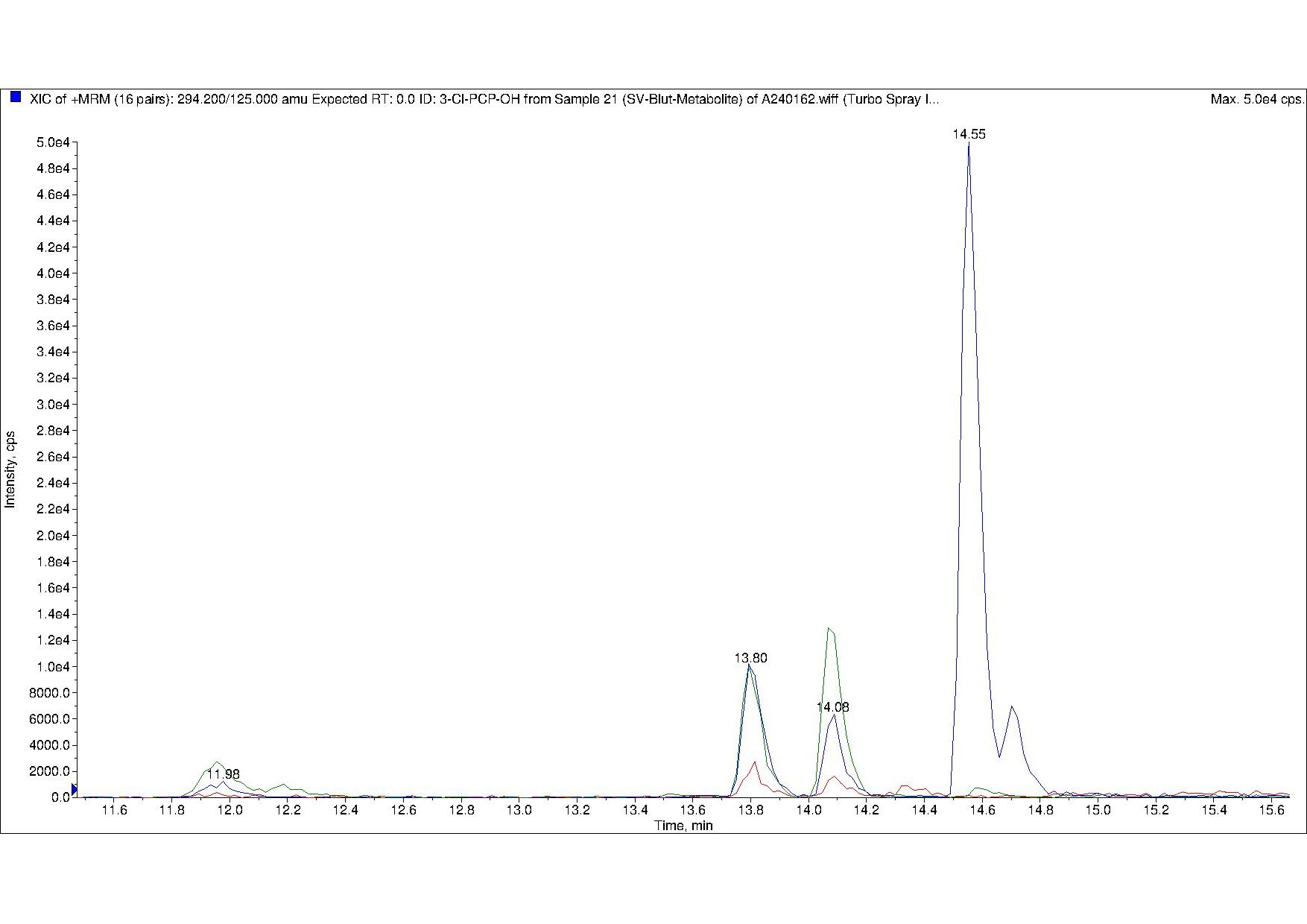  **Figure S7** Chromatogram of the three transitions of the hydroxycyclohexyl metabolites M1–3 in femoral blood (enlarged; blue: 294.2 → 125.0, red: 294.2 → 163.0, green: 294.2 → 86.1) |
| 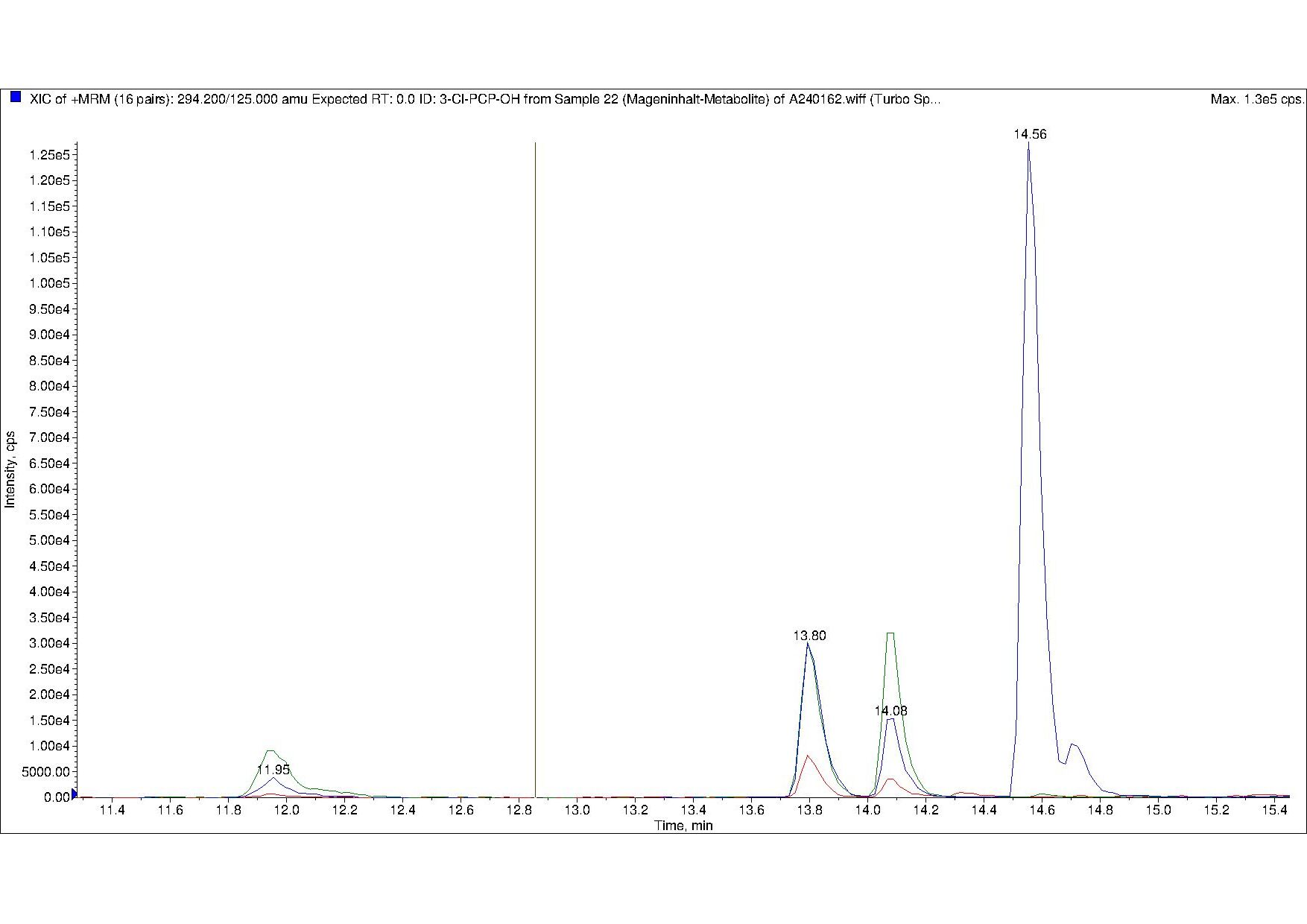  **Figure S8** Chromatogram of the three transitions of the hydroxycyclohexyl metabolites M1–3 in gastric content (enlarged; blue: 294.2 → 125.0, red: 294.2 → 163.0, green: 294.2 → 86.1) |

| **5.2 Hydroxypiperidine metabolites M4-5** |
| --- |
| 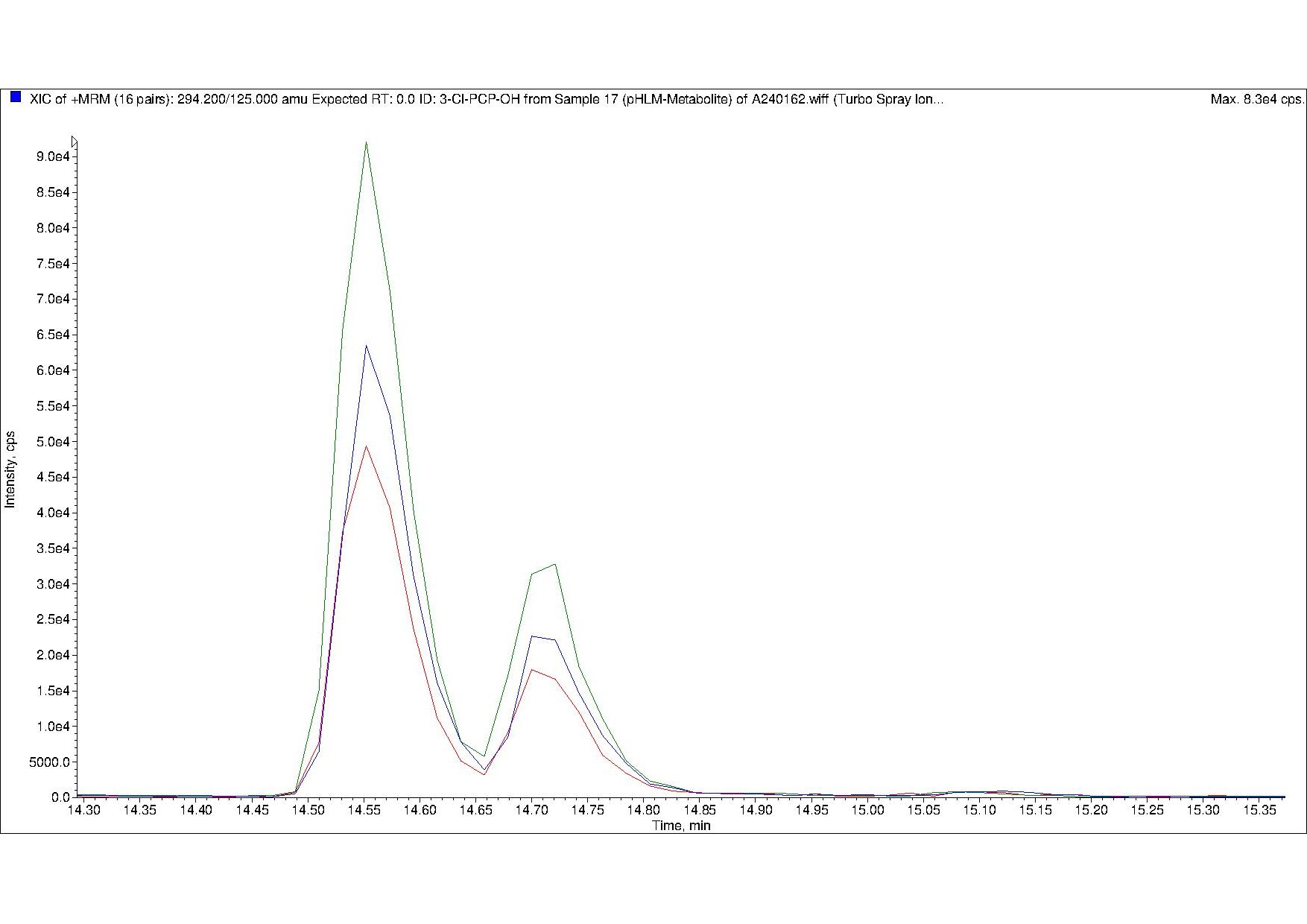  **Figure S9** Chromatogram of the three transitions of the hydroxypiperidine metabolites M4–5 from the pHLM incubation (enlarged; blue: 294.2 → 125.0, red: 294.2 → 193.1, green: 294.2 → 102.1) |

|  |
| --- |
| 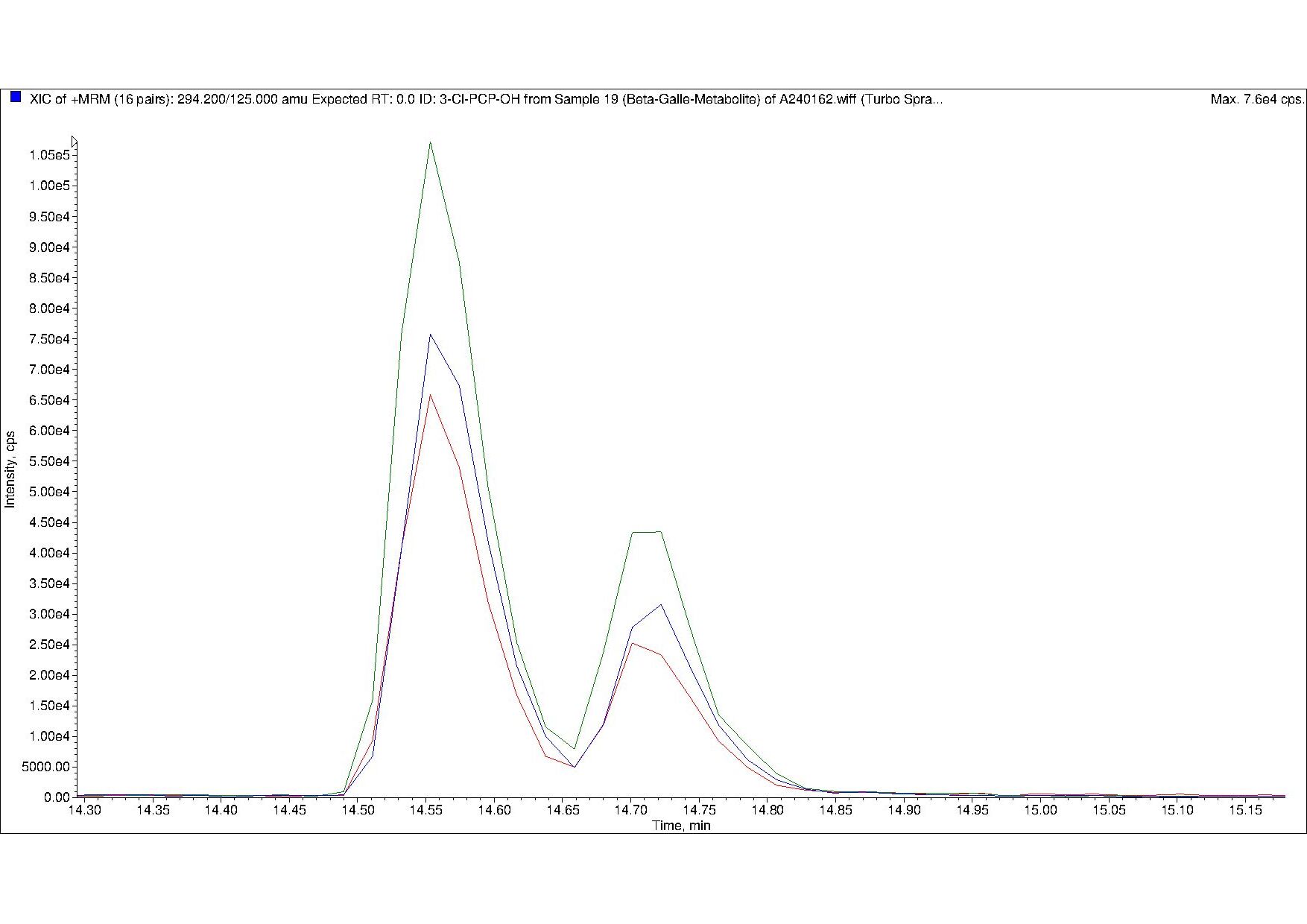  **Figure S10** Chromatogram of the three transitions of the hydroxypiperidine metabolites M4–5 in bile after β-glucuronidase hydrolysis (enlarged; blue: 294.2 → 125.0, red: 294.2 → 193.1, green: 294.2 → 102.1) |
| 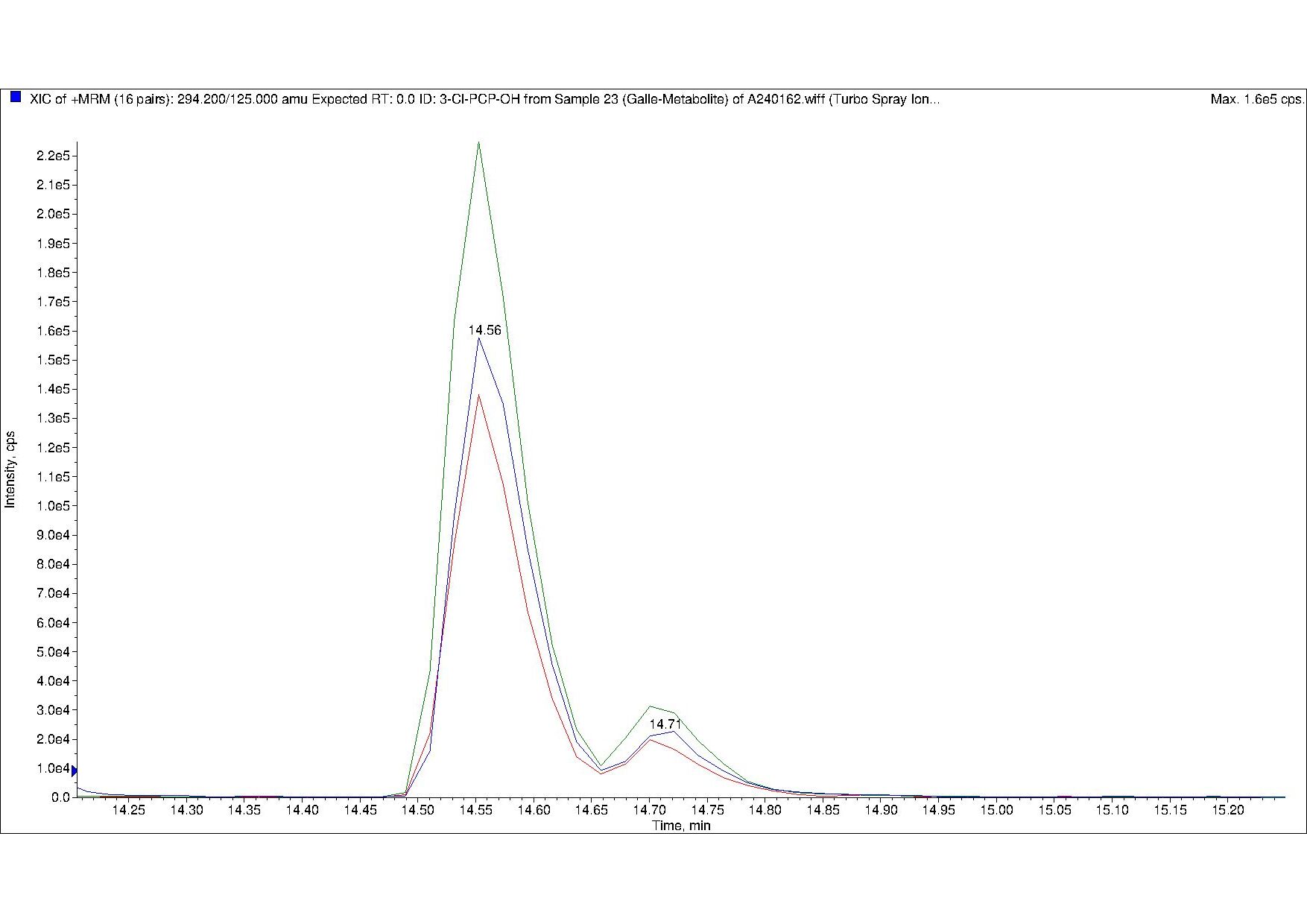  **Figure S11** Chromatogram of the three transitions of the hydroxypiperidine metabolites M4–5 in bile without β-glucuronidase hydrolysis (enlarged; blue: 294.2 → 125.0, red: 294.2 → 193.1, green: 294.2 → 102.1) |
| 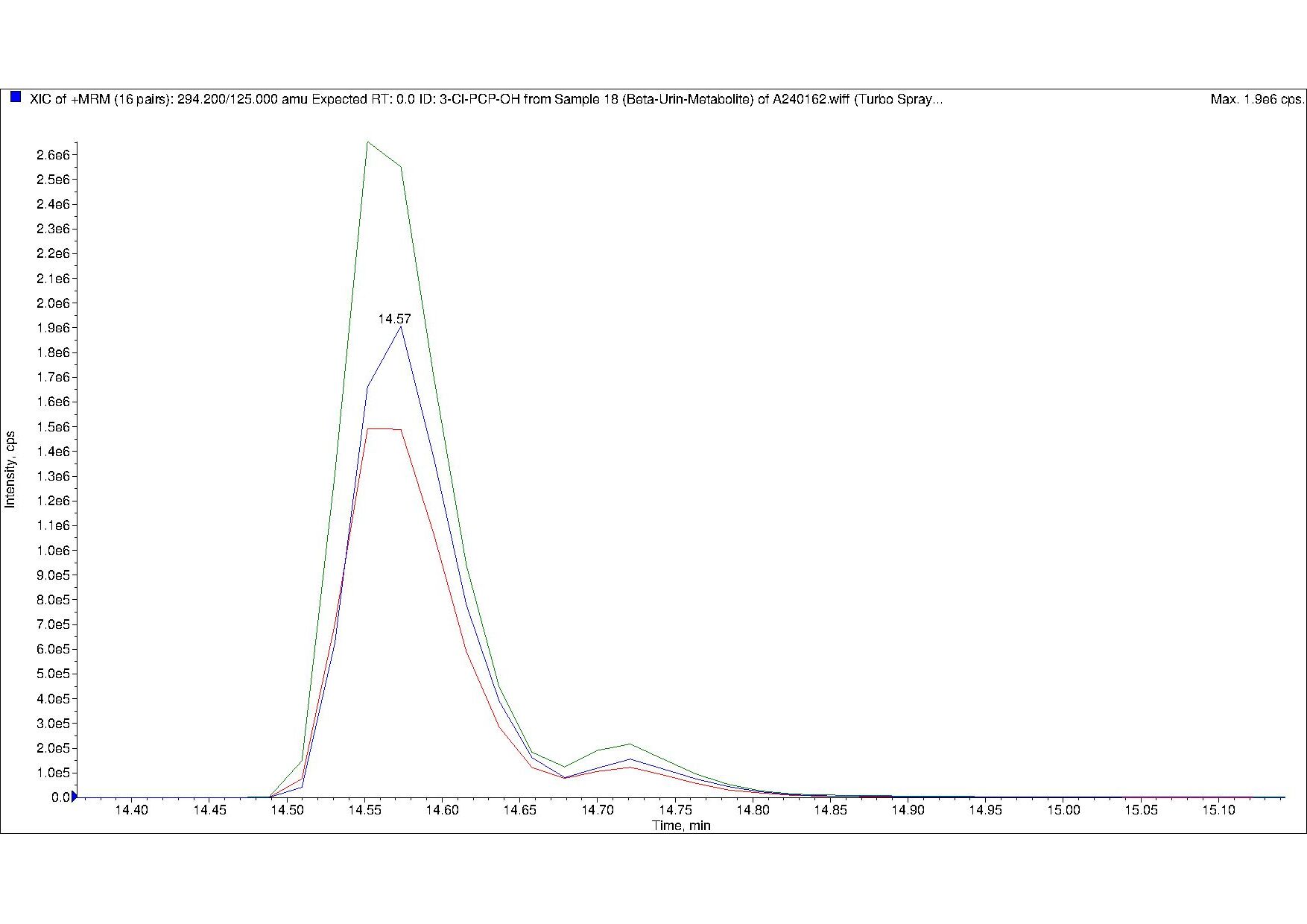  **Figure S12** Chromatogram of the three transitions of the hydroxypiperidine metabolites M4–5 in urine after β-glucuronidase hydrolysis (enlarged; blue: 294.2 → 125.0, red: 294.2 → 193.1, green: 294.2 → 102.1) |
| 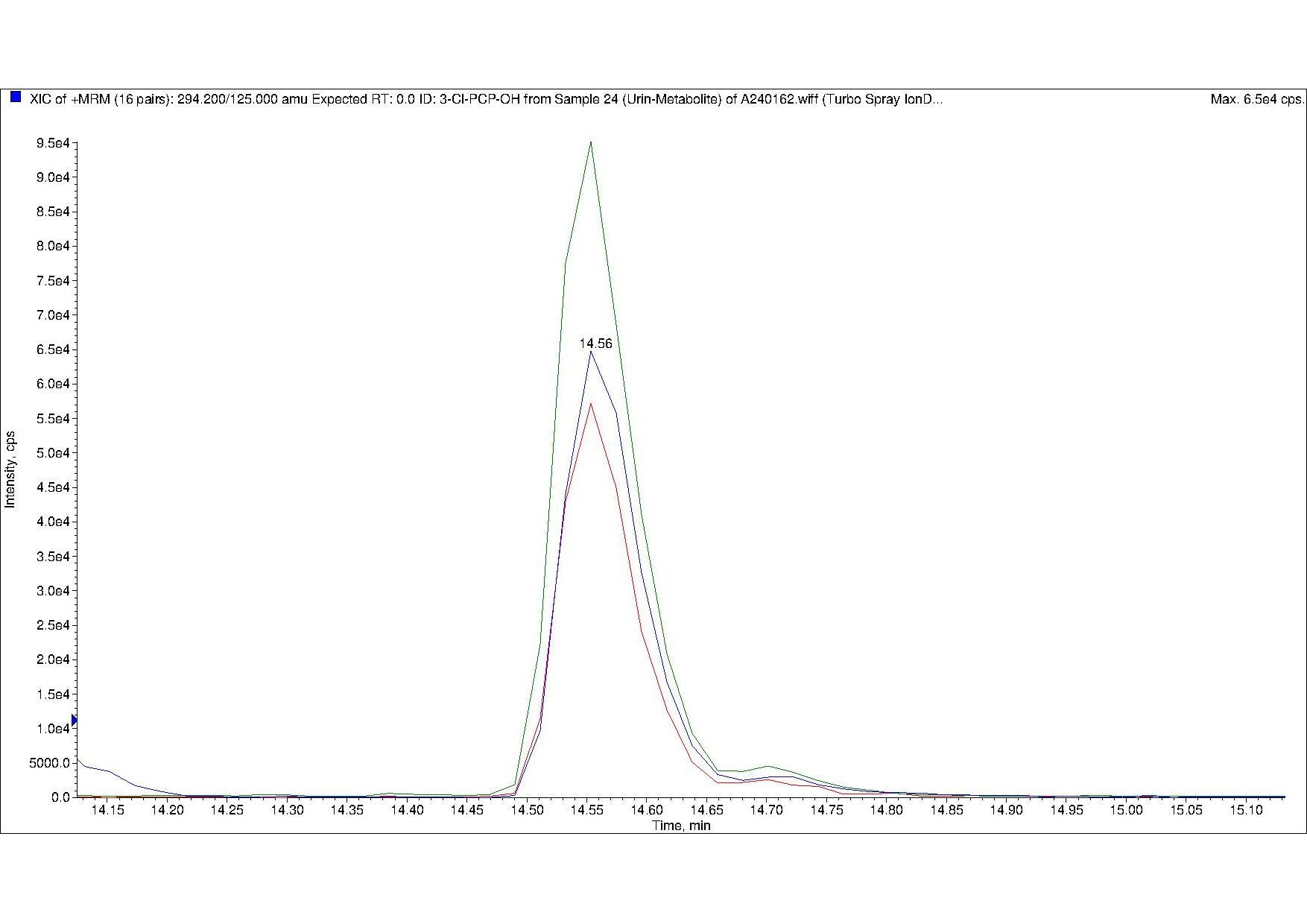  **Figure S13** Chromatogram of the three transitions of the hydroxypiperidine metabolites M4–5 in urine without β-glucuronidase hydrolysis (enlarged; blue: 294.2 → 125.0, red: 294.2 → 193.1, green: 294.2 → 102.1) |
| 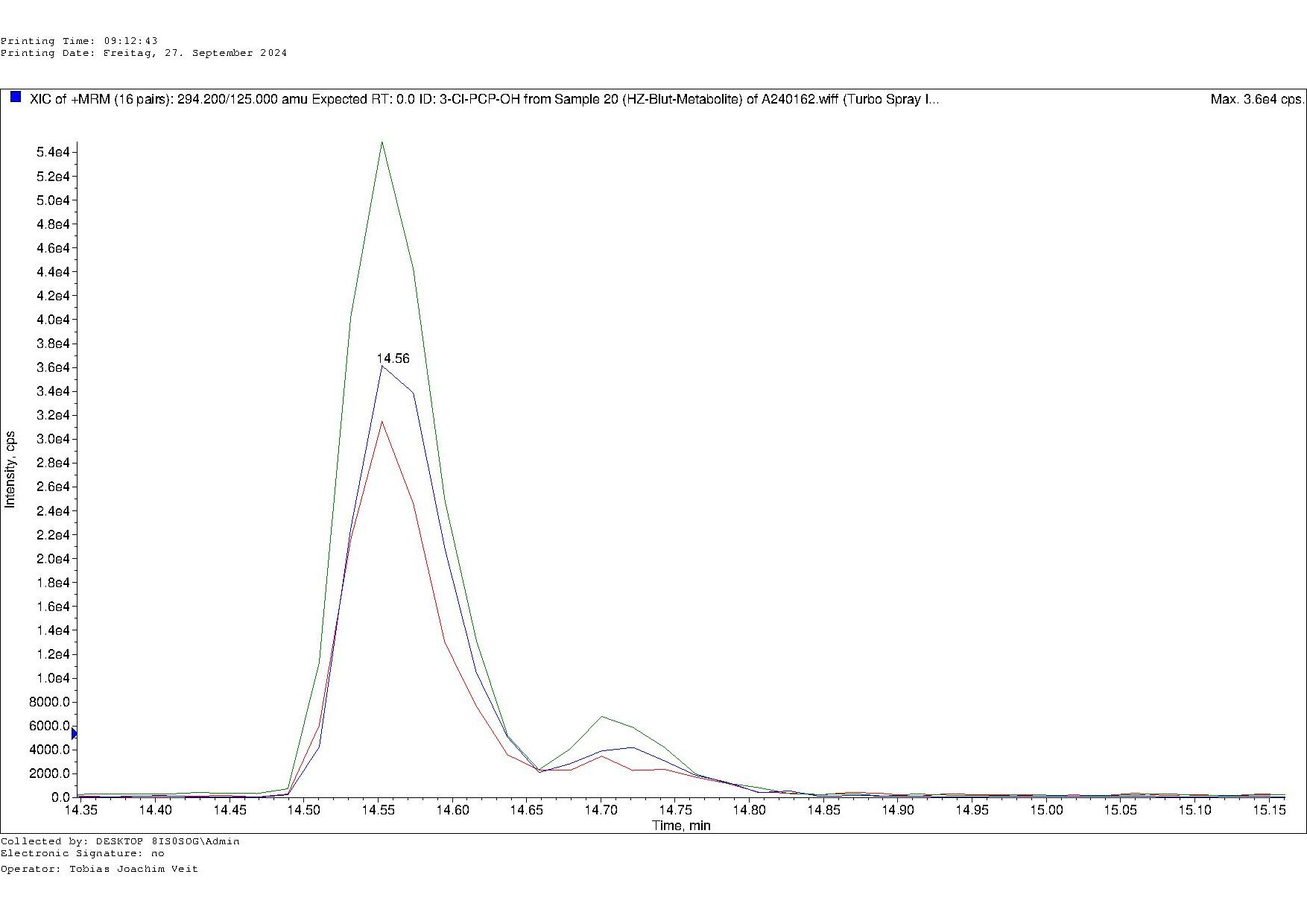  **Figure S14** Chromatogram of the three transitions of the hydroxypiperidine metabolites M4–5 in cardiac blood (enlarged; blue: 294.2 → 125.0, red: 294.2 → 193.1, green: 294.2 → 102.1) |
| 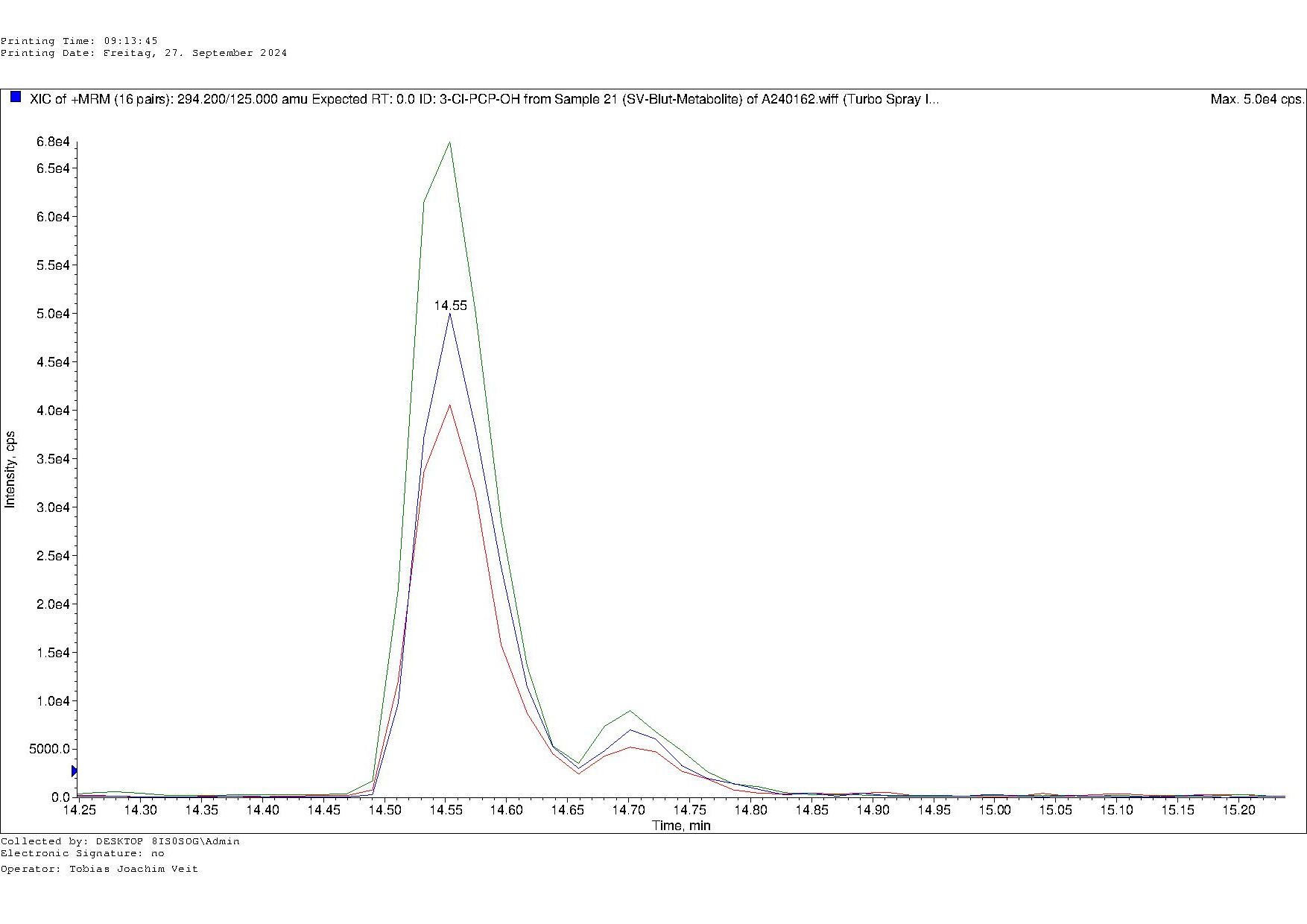  **Figure S15** Chromatogram of the three transitions of the hydroxypiperidine metabolites M4–5 in femoral blood (enlarged; blue: 294.2 → 125.0, red: 294.2 → 193.1, green: 294.2 → 102.1) |
| 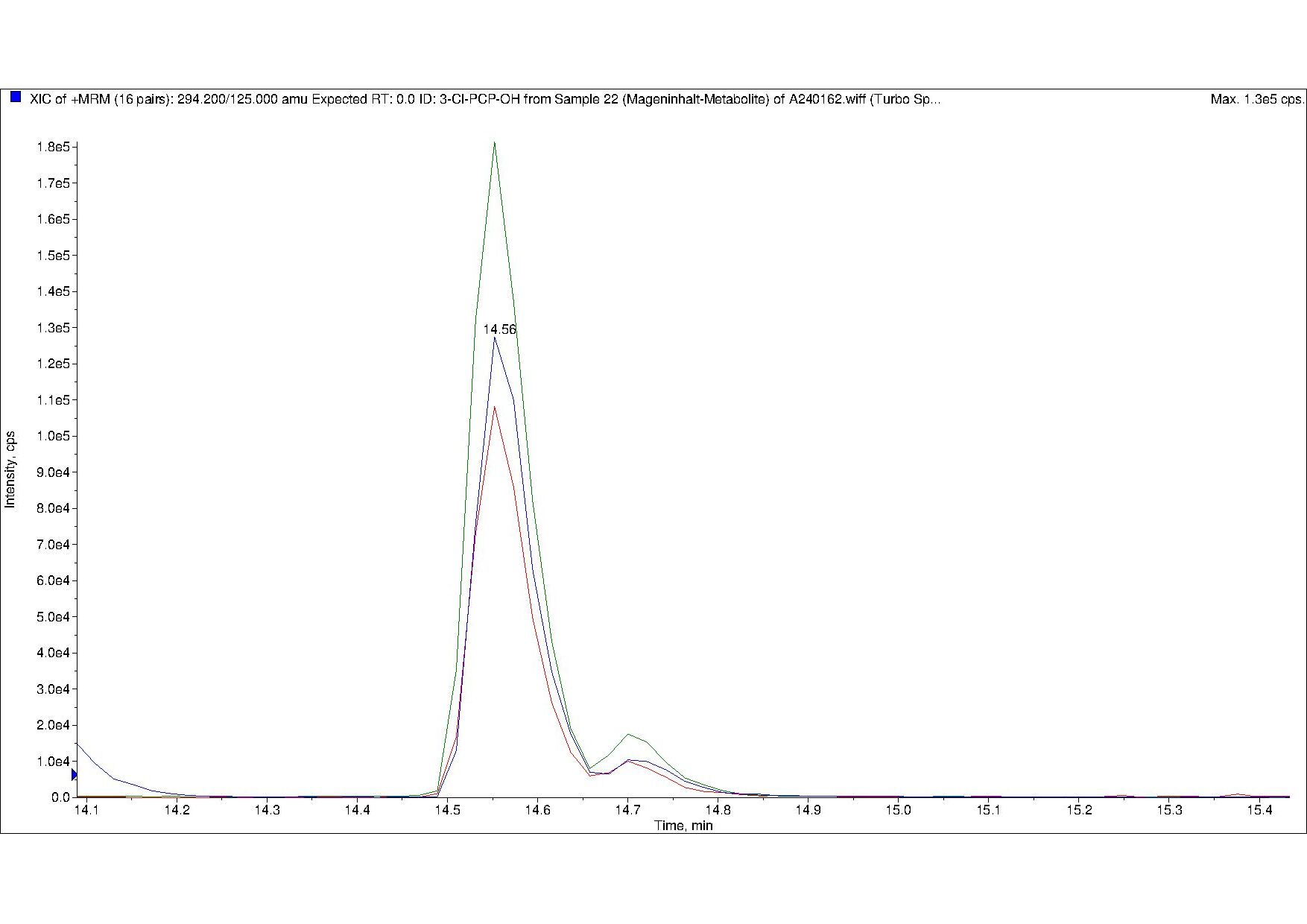  **Figure S16** Chromatogram of the three transitions of the hydroxypiperidine metabolites M4–5 in gastric content (enlarged; blue: 294.2 → 125.0, red: 294.2 → 193.1, green: 294.2 → 102.1) |

|  |
| --- |
|  |
| **5.3 Carboxyl metabolite M6**  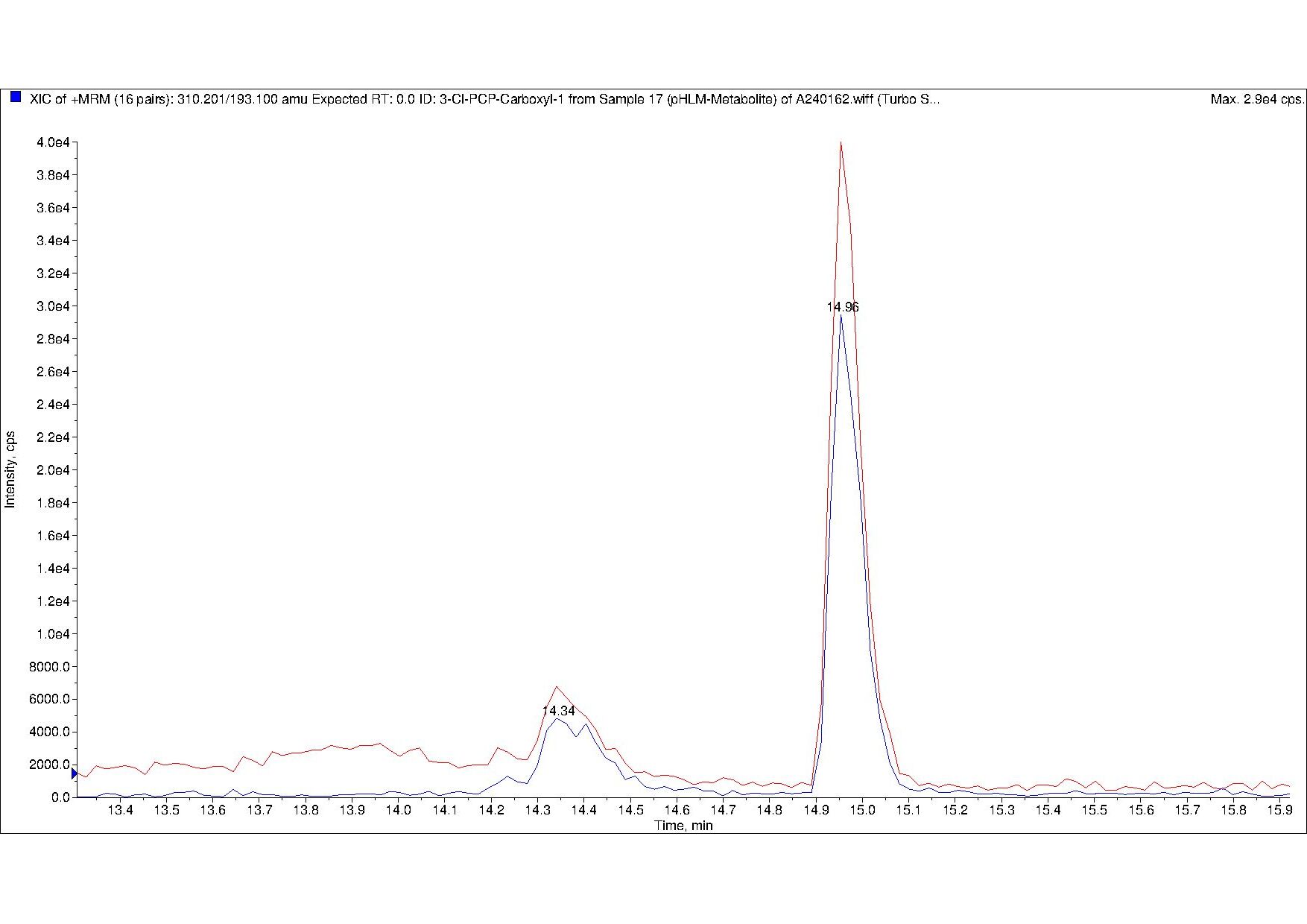  **Figure S17** Chromatogram of the two transitions of the carboxyl metabolite M6 from the pHLM incubation (enlarged; blue: 310.2 → 193.0, red: 310.2 → 118.1) |
| 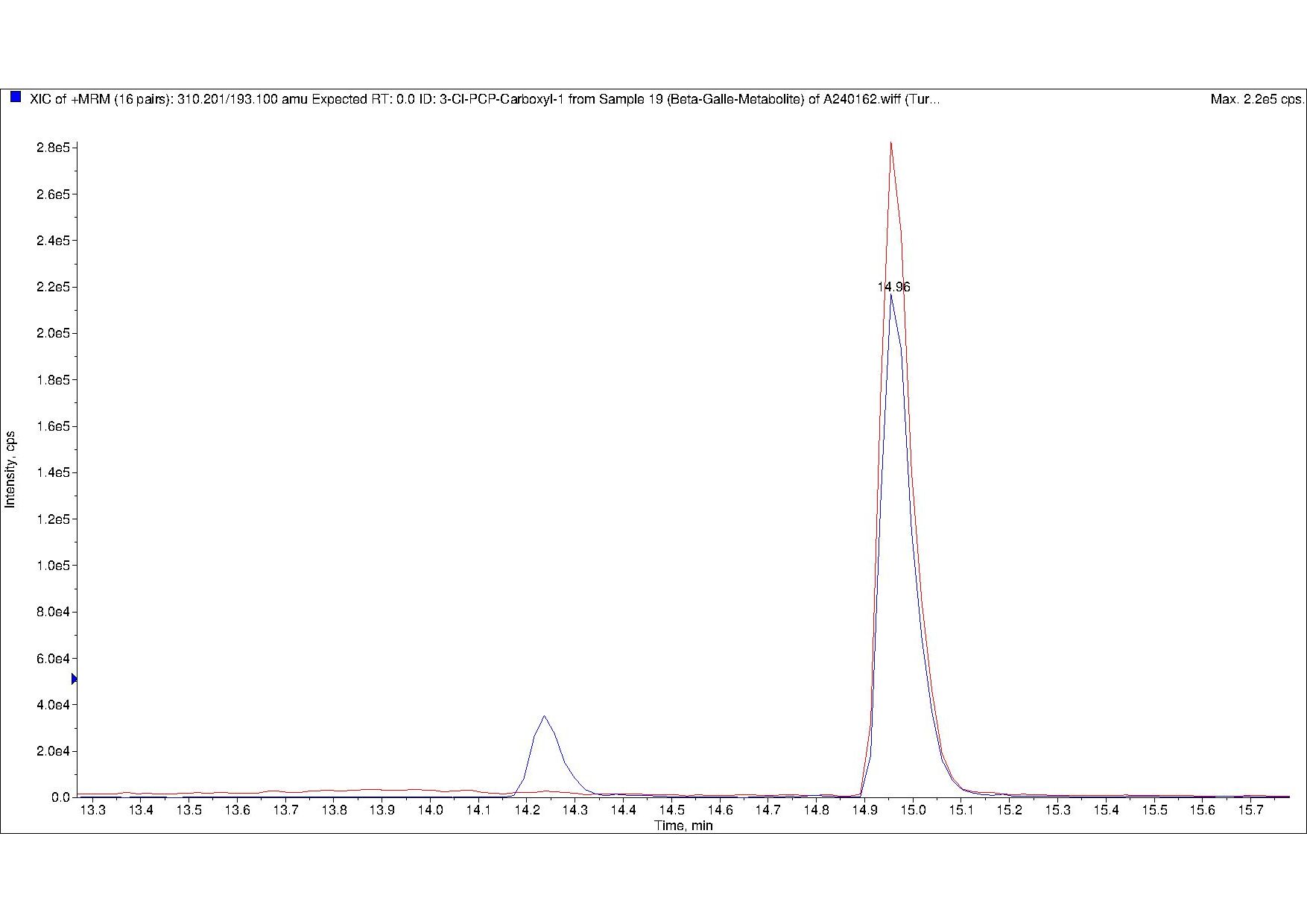  **Figure S18** Chromatogram of the two transitions of the carboxyl metabolite M6 in bile after β-glucuronidase hydrolysis (enlarged; blue: 310.2 → 193.0, red: 310.2 → 118.1) |
| 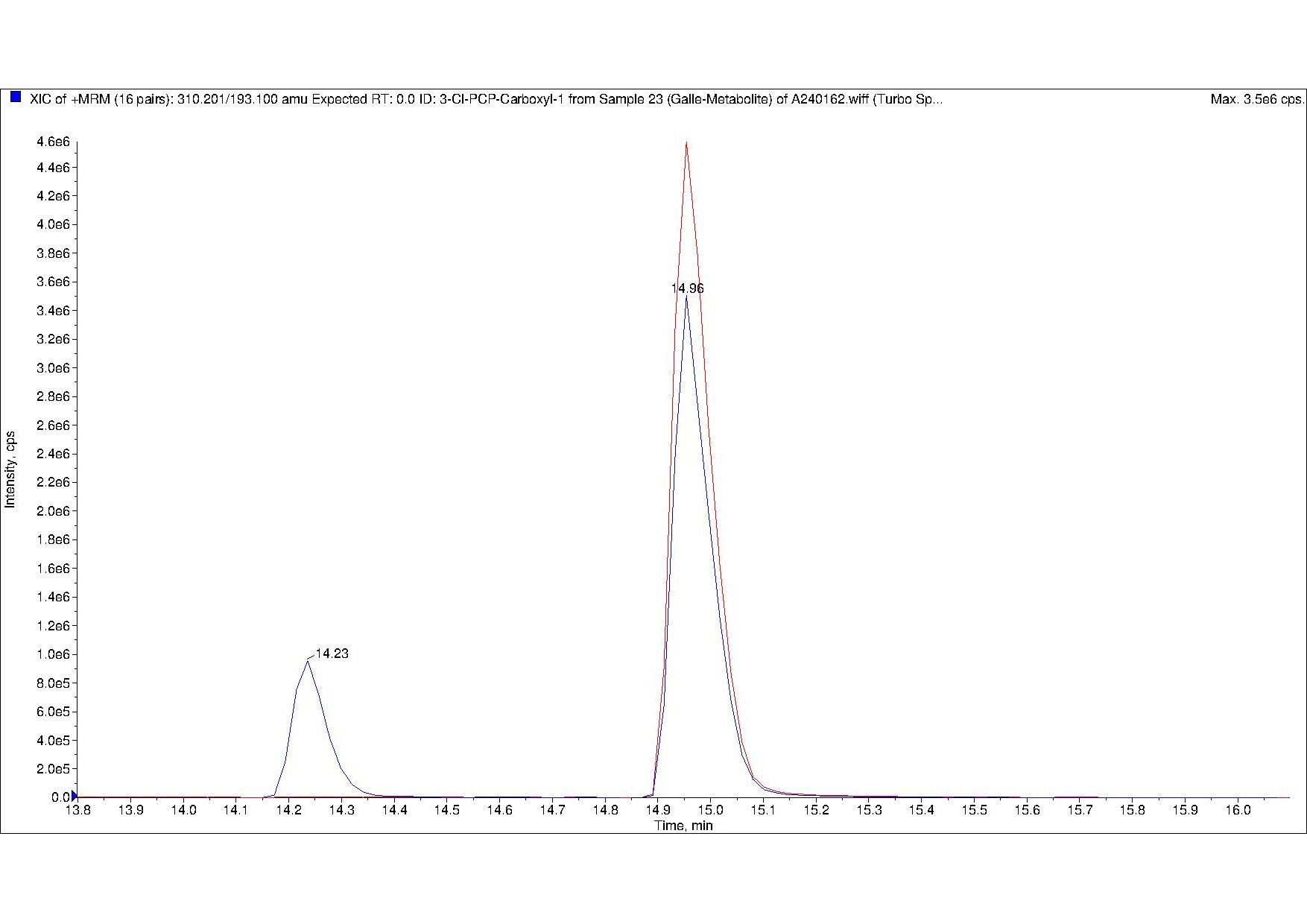  **Figure S19** Chromatogram of the two transitions of the carboxyl metabolite M6 in bile without β-glucuronidase hydrolysis (enlarged; blue: 310.2 → 193.0, red: 310.2 → 118.1) |
| 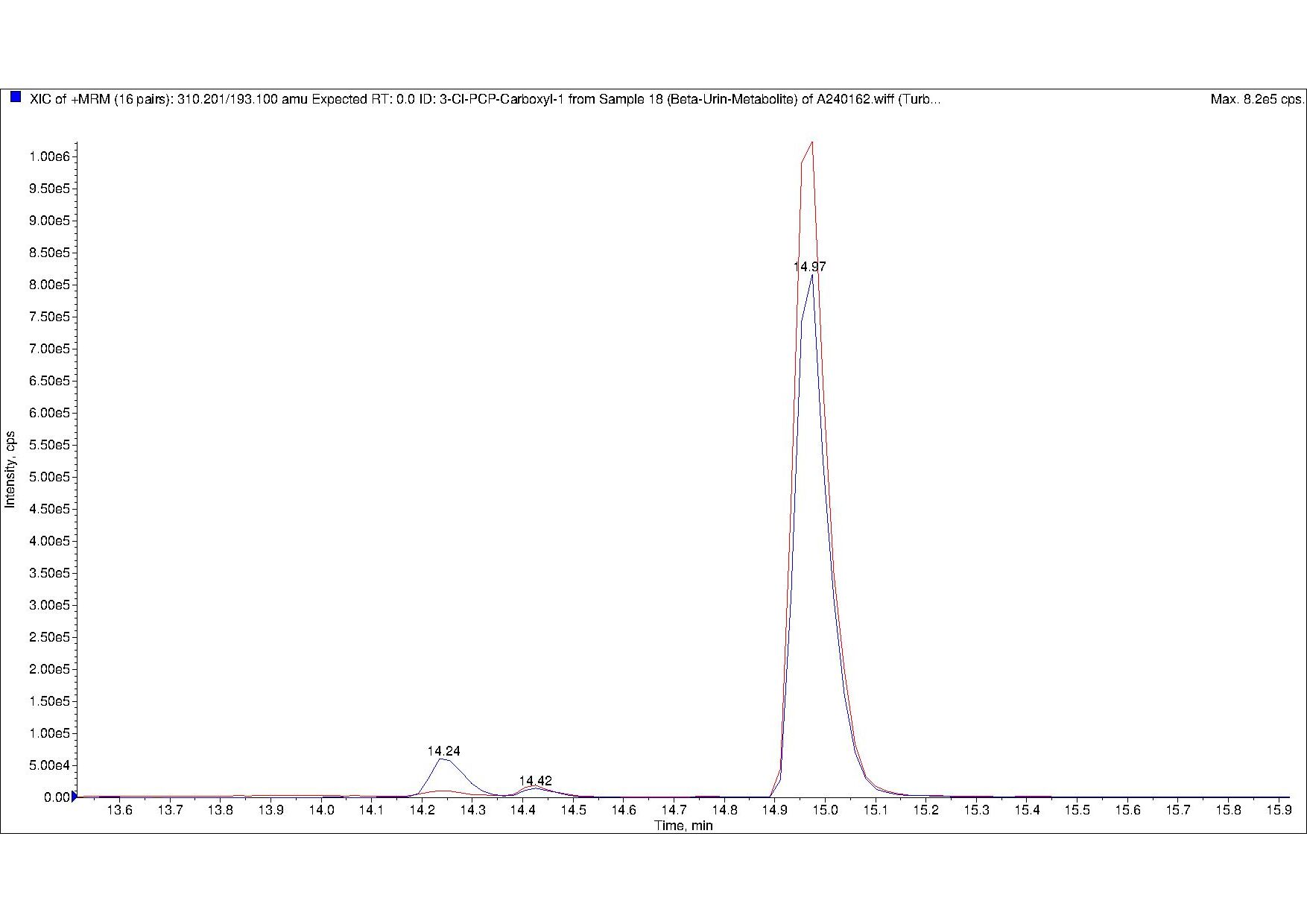  **Figure S20** Chromatogram of the two transitions of the carboxyl metabolite M6 in urine after β-glucuronidase hydrolysis (enlarged; blue: 310.2 → 193.0, red: 310.2 → 118.1) |
| 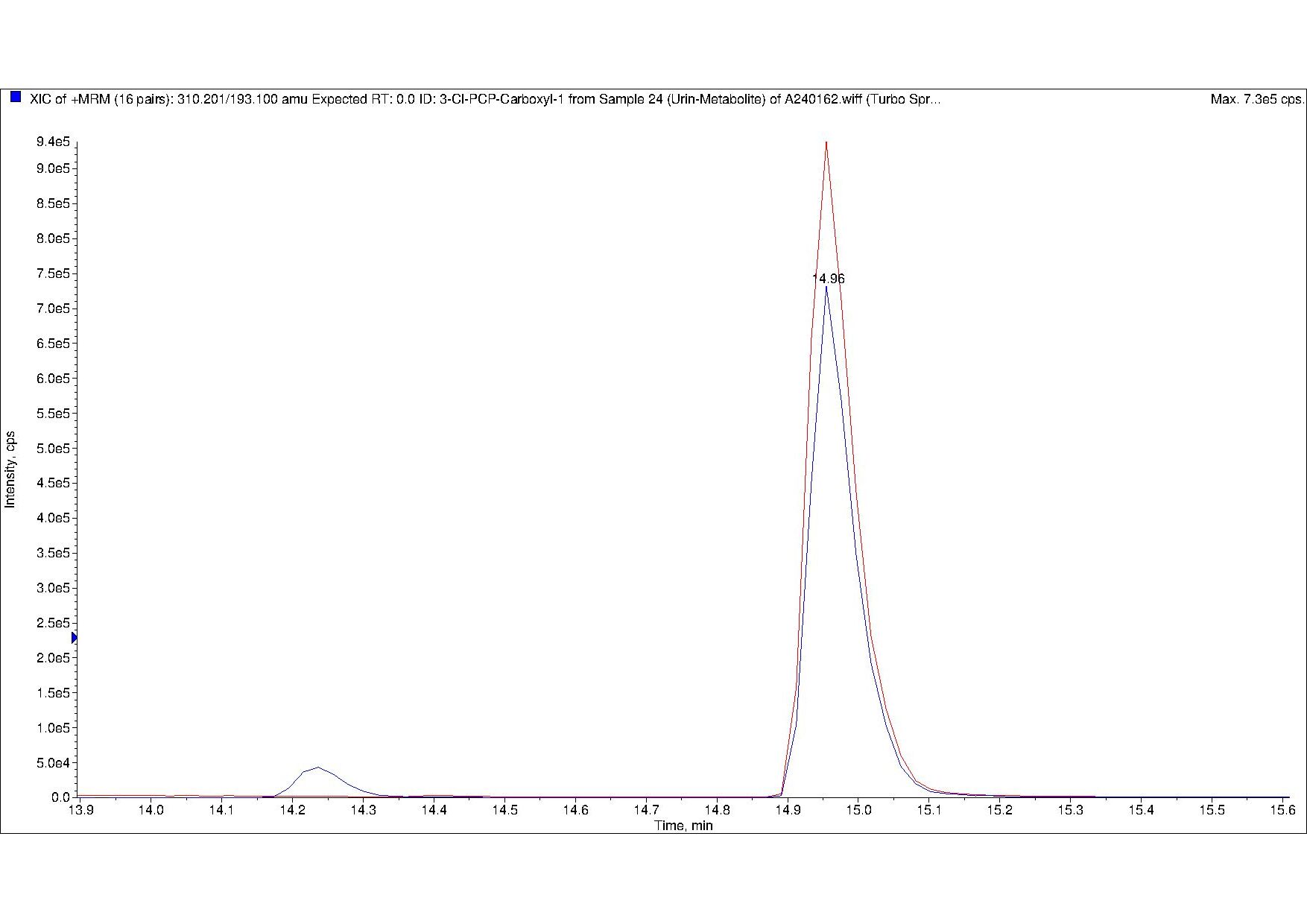  **Figure S21** Chromatogram of the two transitions of the carboxyl metabolite M6 in urine without β-glucuronidase hydrolysis (enlarged; blue: 310.2 → 193.0, red: 310.2 → 118.1) |
| 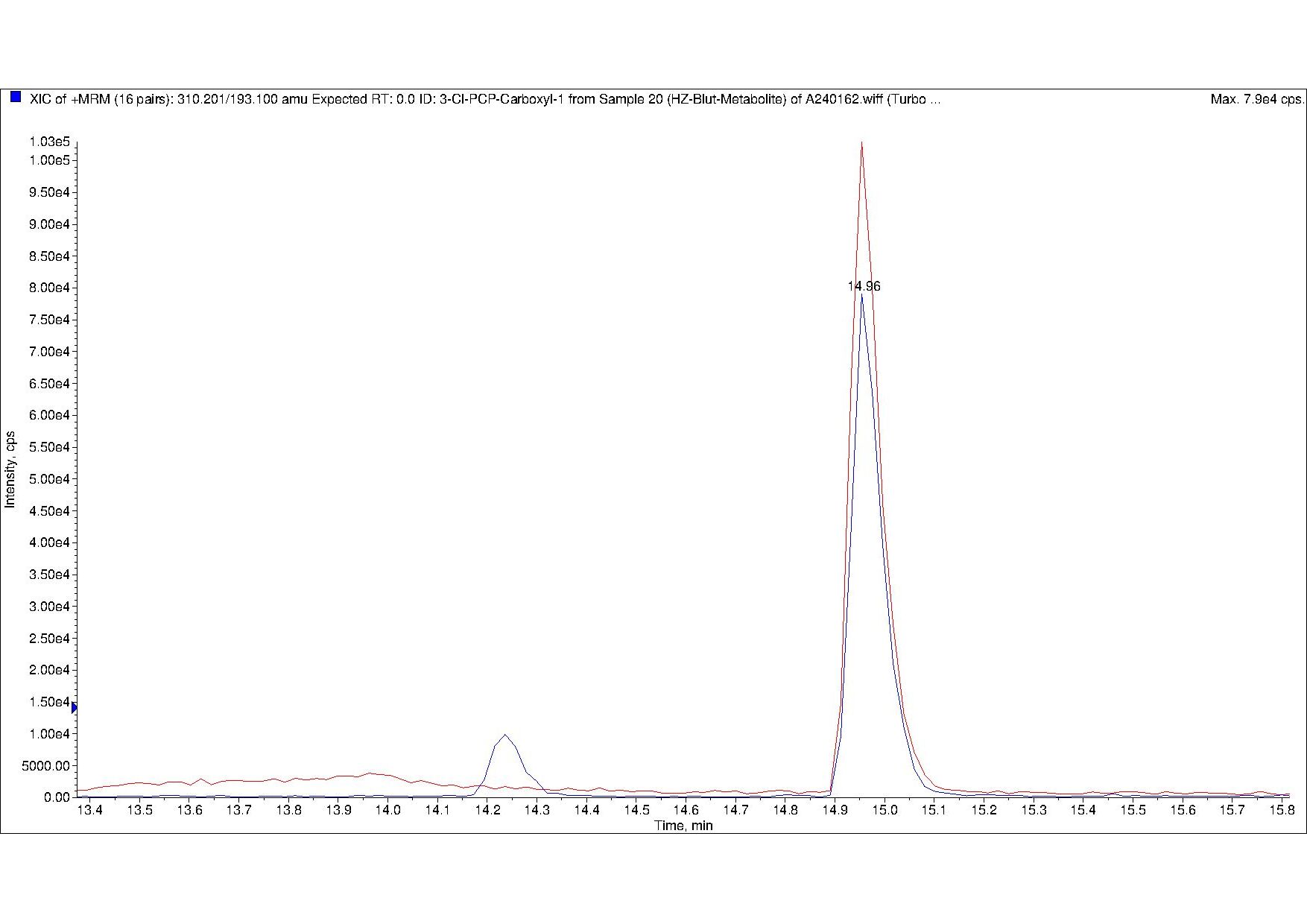  **Figure S22** Chromatogram of the two transitions of the carboxyl metabolite M6 in cardiac blood (enlarged; blue: 310.2 → 193.0, red: 310.2 → 118.1) |
| 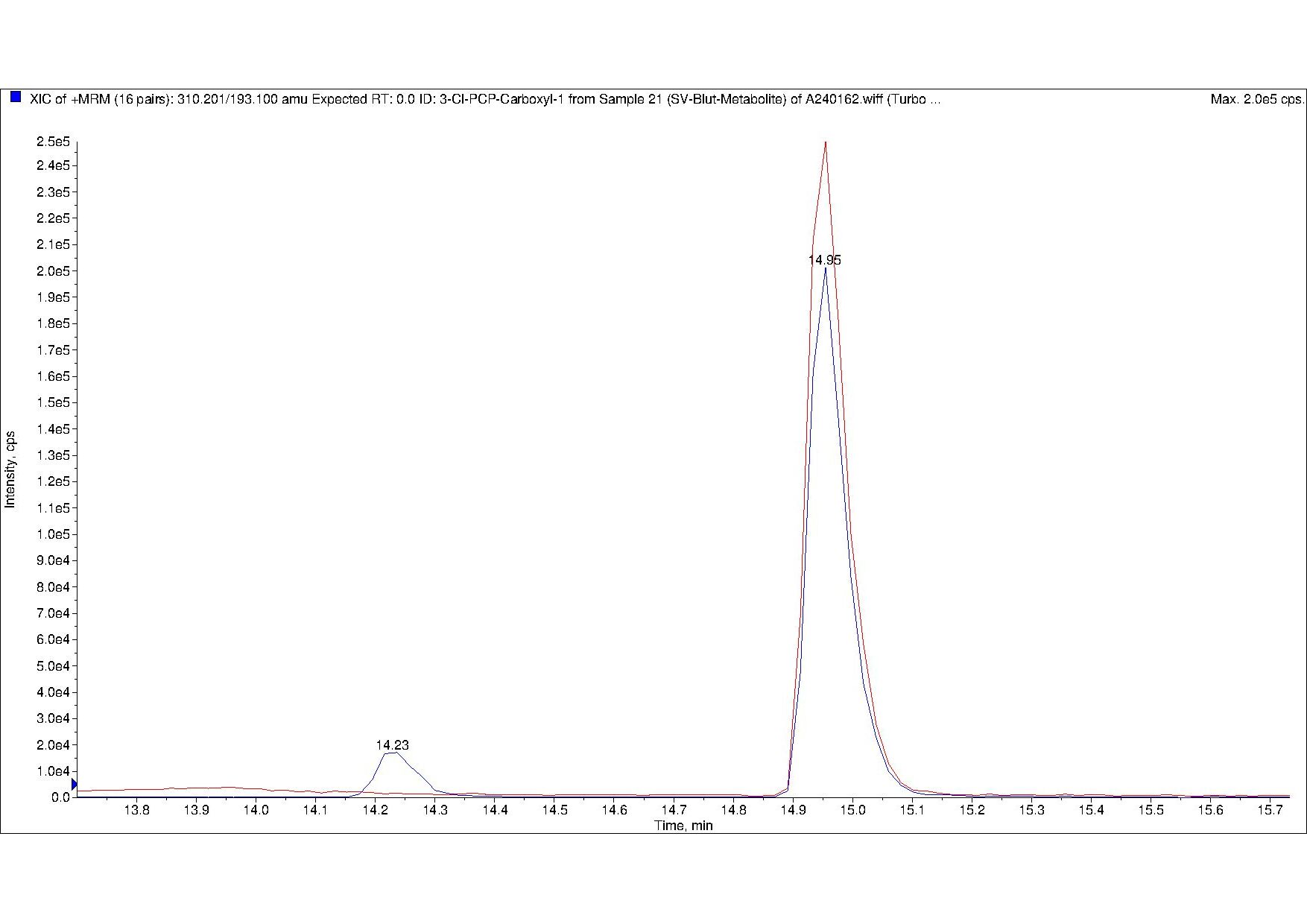  **Figure S23** Chromatogram of the two transitions of the carboxyl metabolite M6 in femoral blood (enlarged; blue: 310.2 → 193.0, red: 310.2 → 118.1) |
| 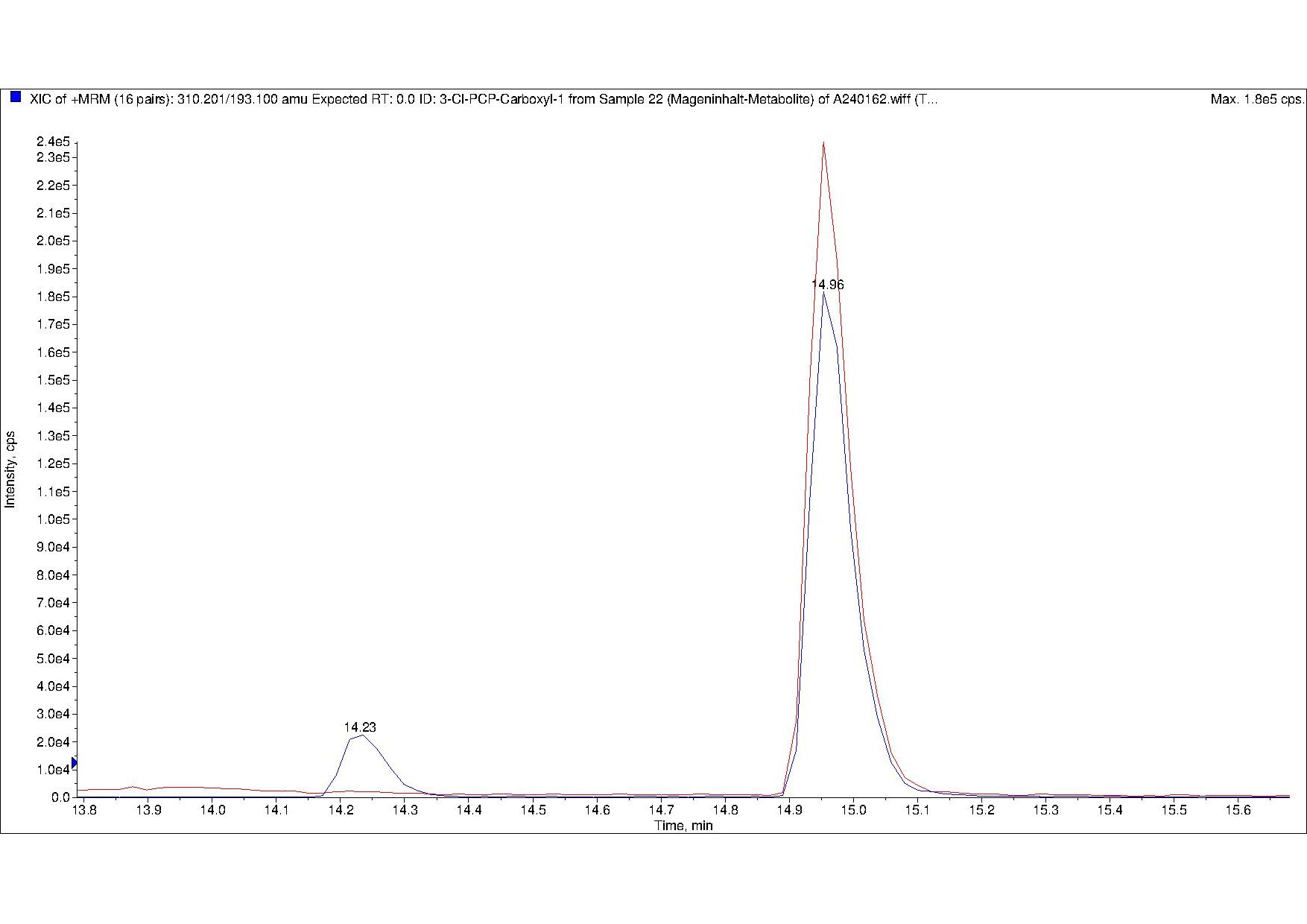  **Figure S24** Chromatogram of the two transitions of the carboxyl metabolite M6 in gastric content (enlarged; blue: 310.2 → 193.0, red: 310.2 → 118.1) |

| **5.4 Alcohol metabolite M7**  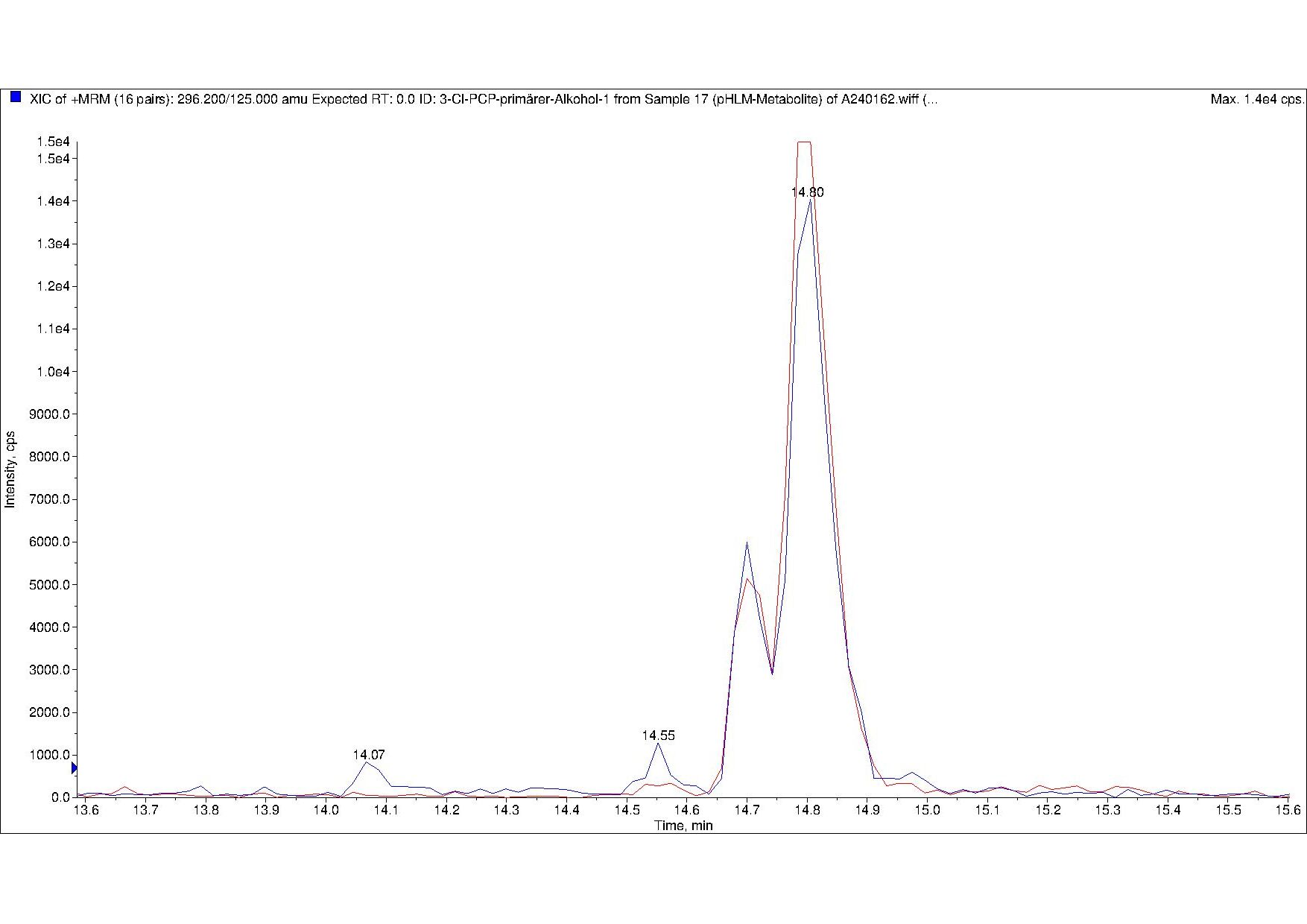  **Figure S25** Chromatogram of the two transitions of the alcohol metabolite M7 from the pHLM incubation (enlarged; blue: 296.2 → 125.0, red: 296.2 → 104.1) |
| --- |
| 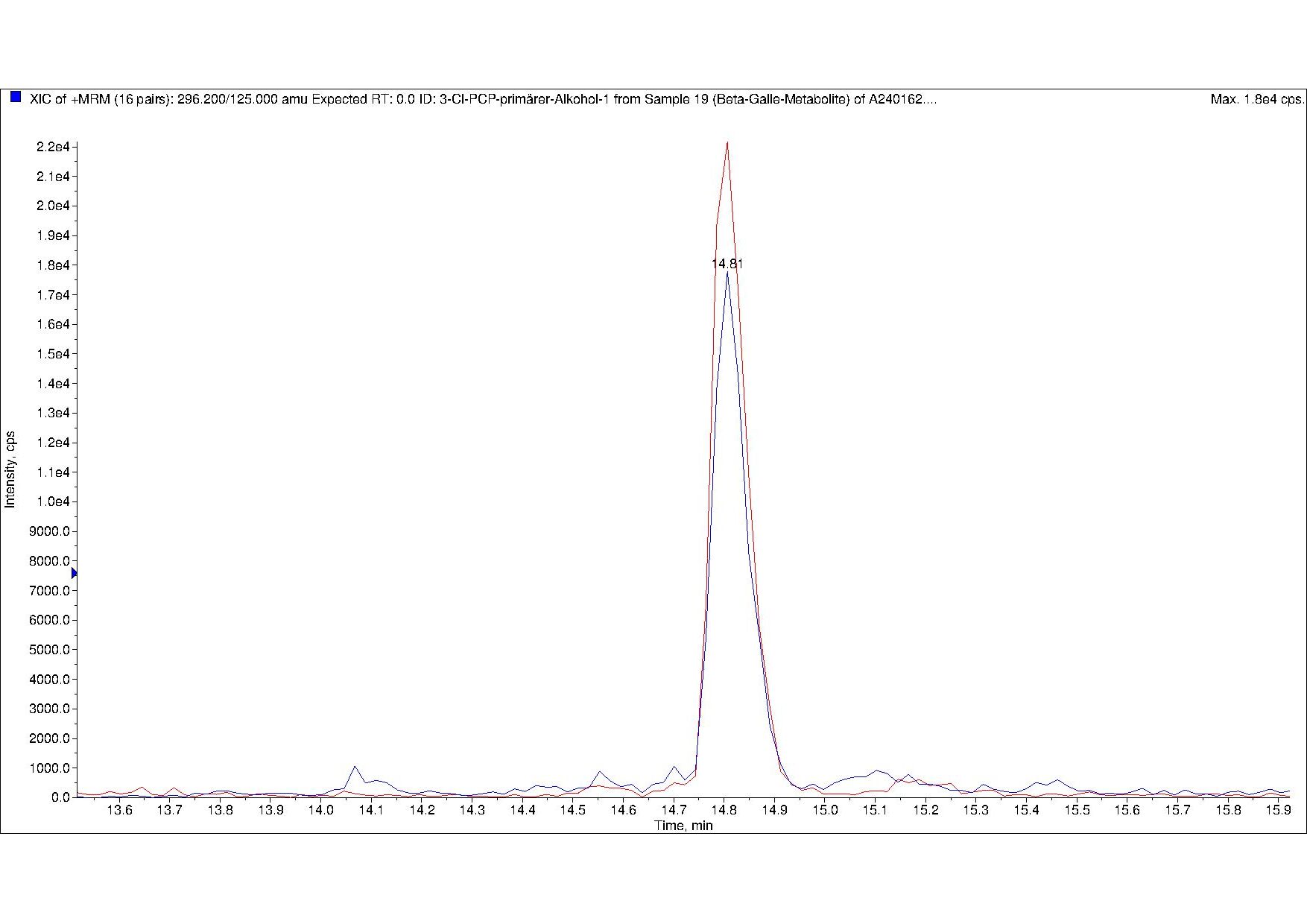  **Figure S26** Chromatogram of the two transitions of the alcohol metabolite M7 in bile with β-glucuronidase hydrolysis (enlarged; blue: 296.2 → 125.0, red: 296.2 → 104.1) |
| 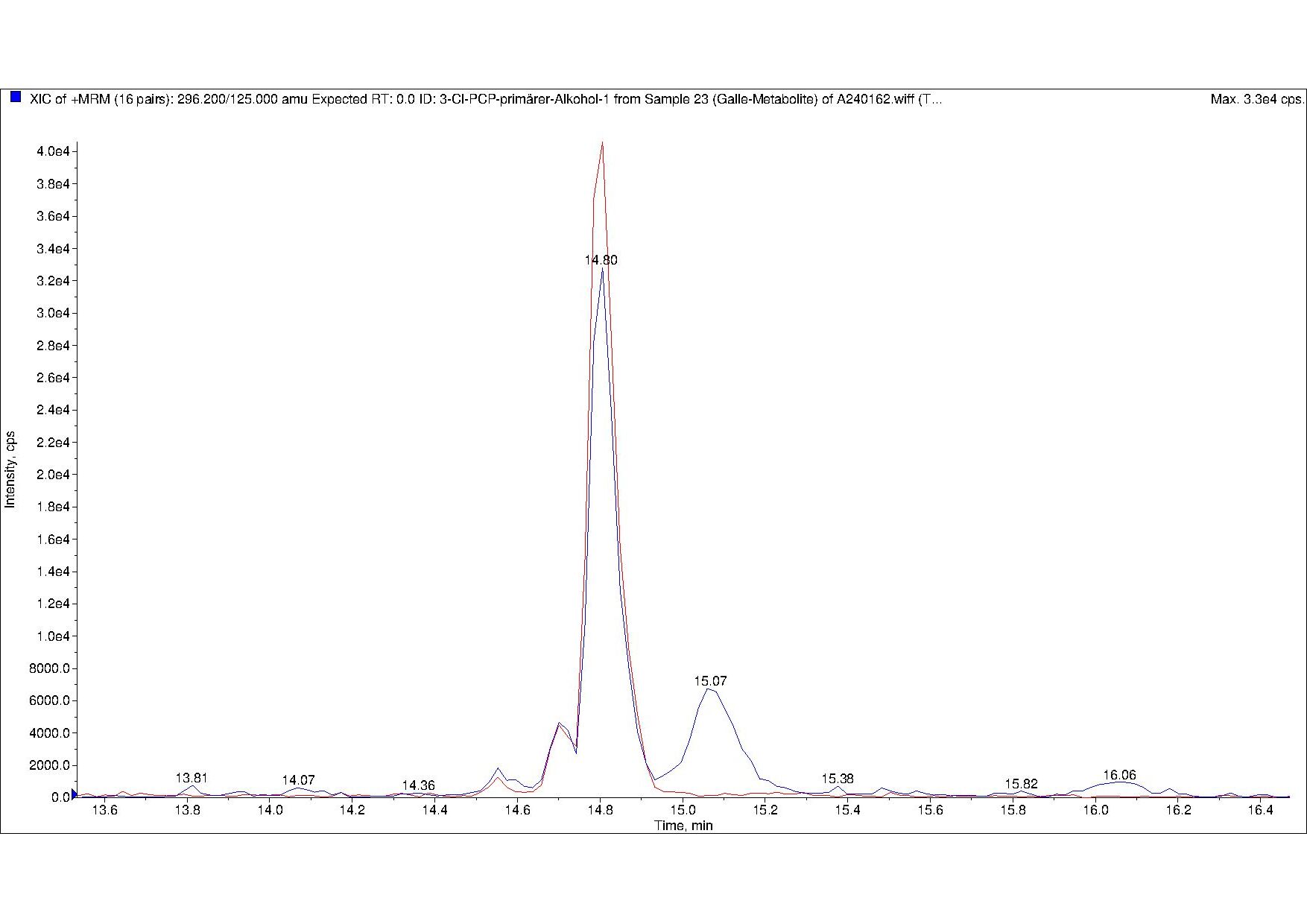  **Figure S27** Chromatogram of the two transitions of the alcohol metabolite M7 in bile without β-glucuronidase hydrolysis (enlarged; blue: 296.2 → 125.0, red: 296.2 → 104.1) |
| 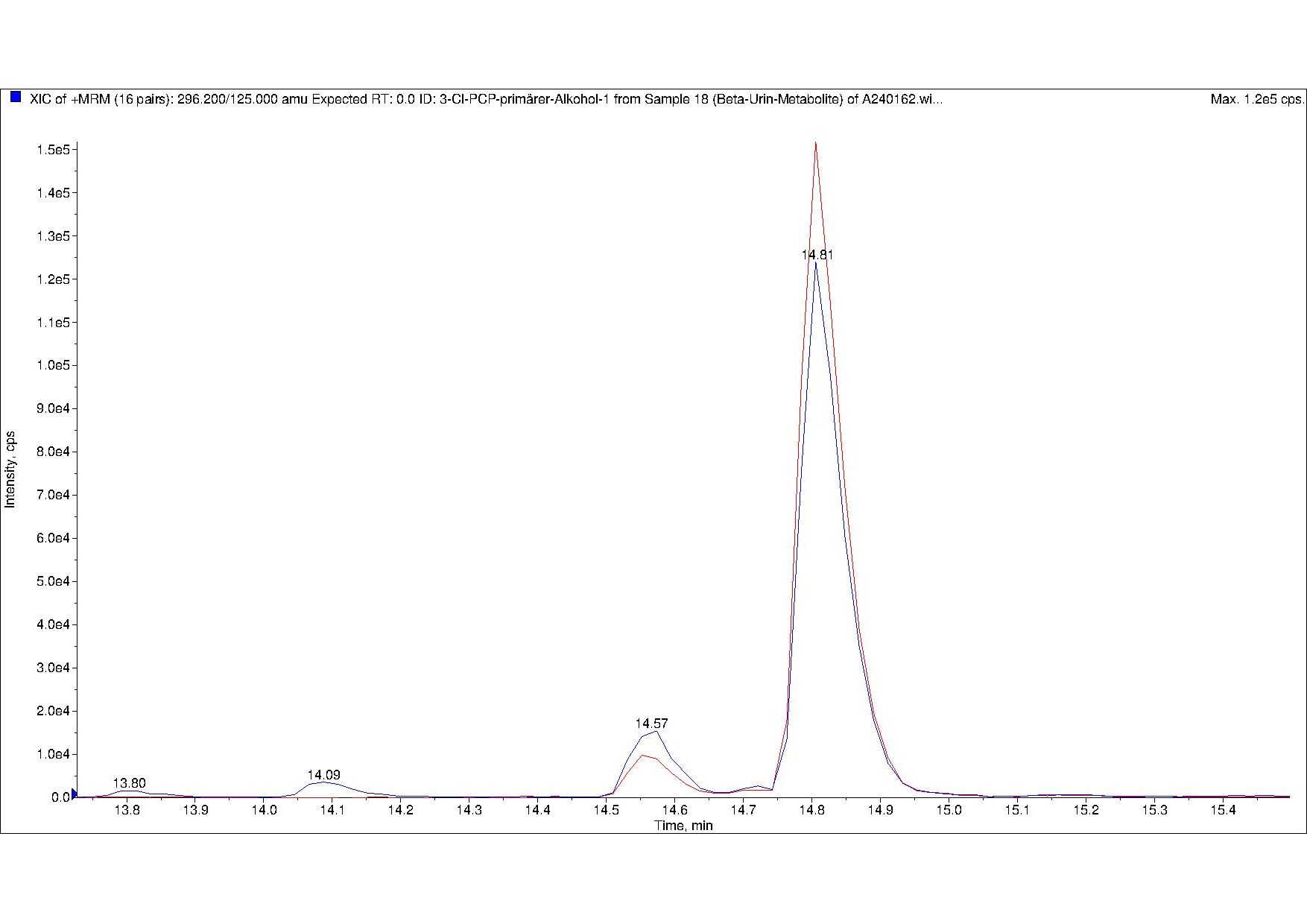  **Figure S28** Chromatogram of the two transitions of the alcohol metabolite M7 in urine with β-glucuronidase hydrolysis (enlarged; blue: 296.2 → 125.0, red: 296.2 → 104.1) |
| 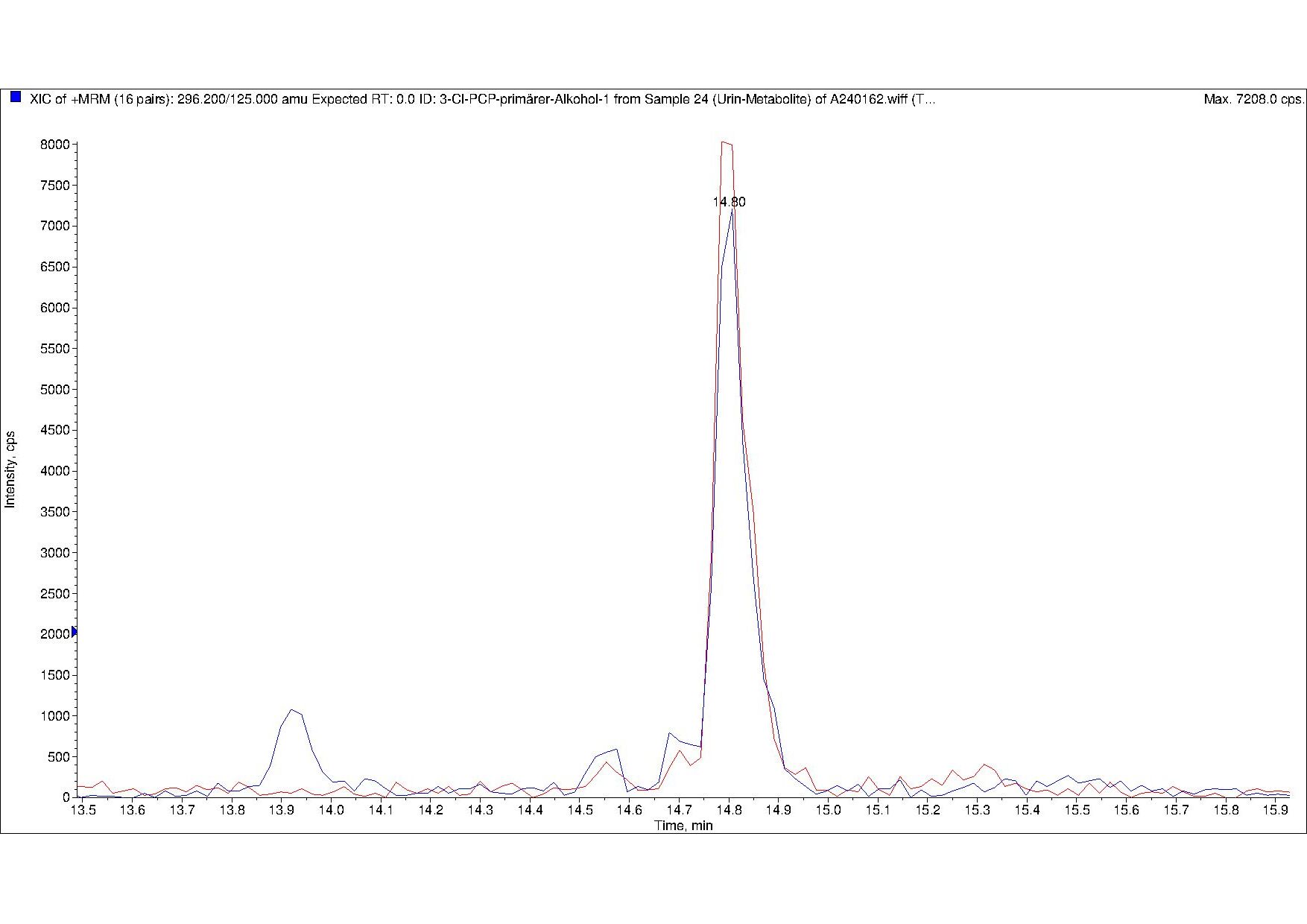  **Figure S29** Chromatogram of the two transitions of the alcohol metabolite M7 in urine without β-glucuronidase hydrolysis (enlarged; blue: 296.2 → 125.0, red: 296.2 → 104.1) |
| 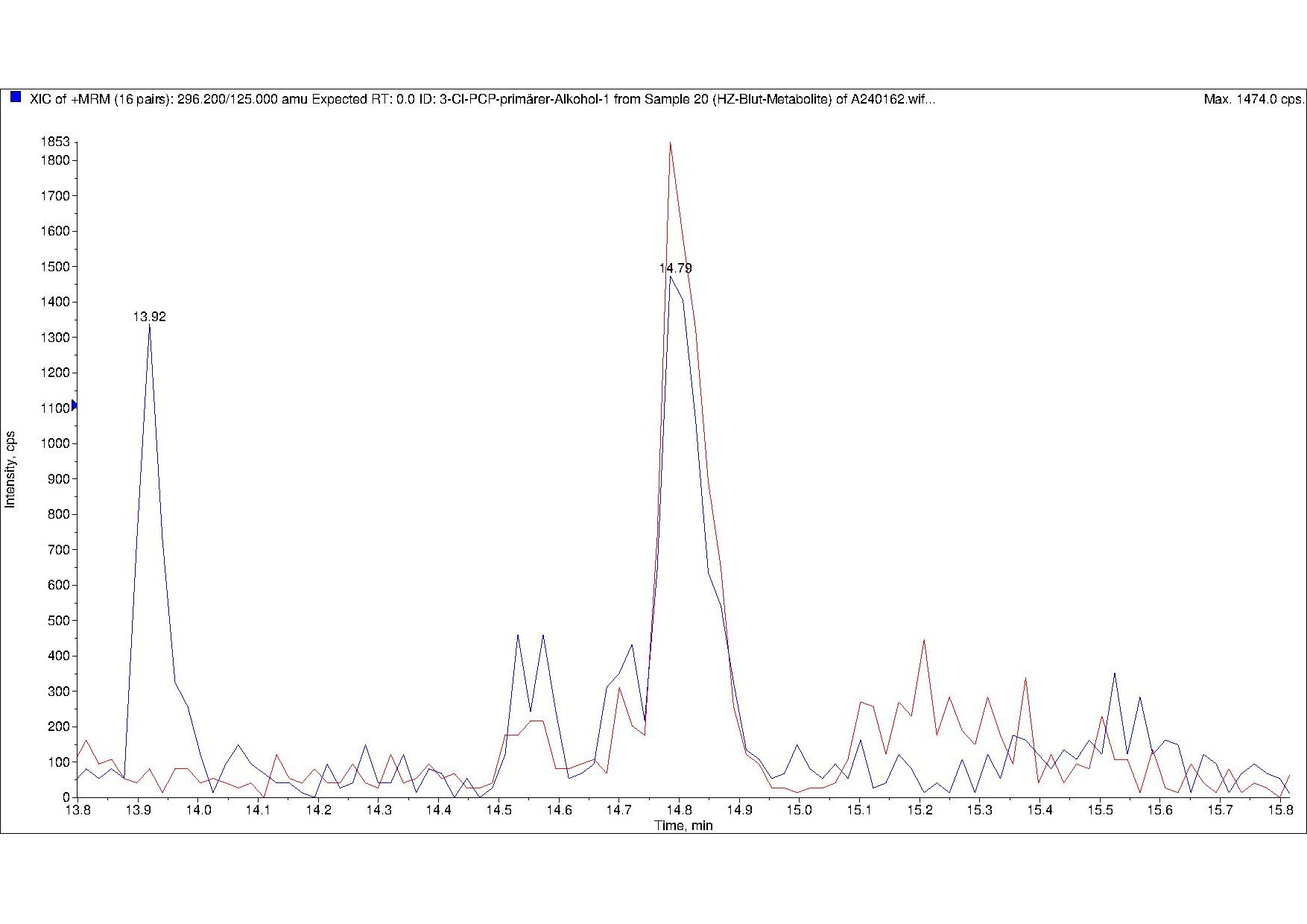  **Figure S30** Chromatogram of the two transitions of the alcohol metabolite M7 in cardiac blood (enlarged; blue: 296.2 → 125.0, red: 296.2 → 104.1) |
| 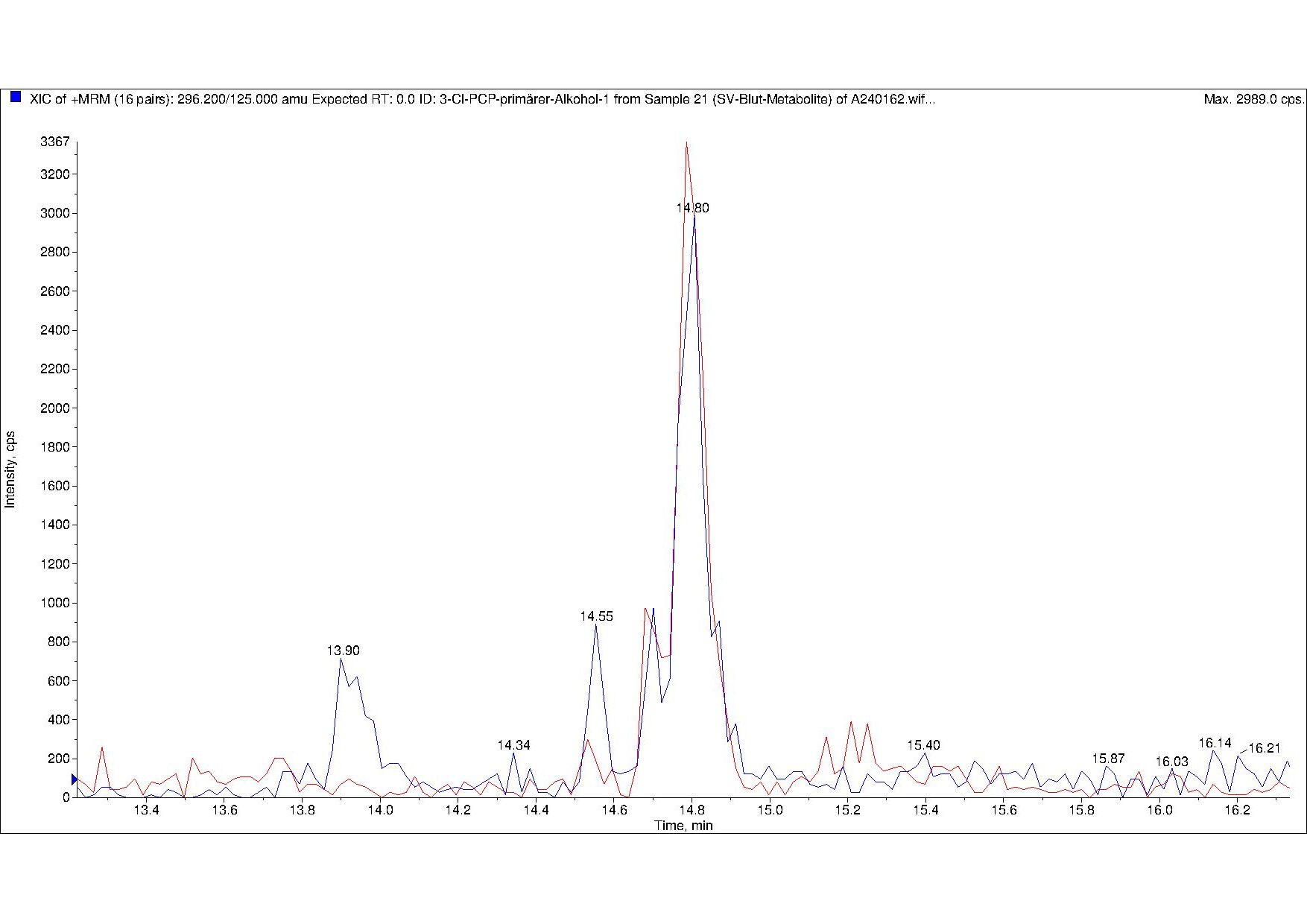  **Figure S31** Chromatogram of the two transitions of the alcohol metabolite M7 in femoral blood (enlarged; blue: 296.2 → 125.0, red: 296.2 → 104.1) |
| 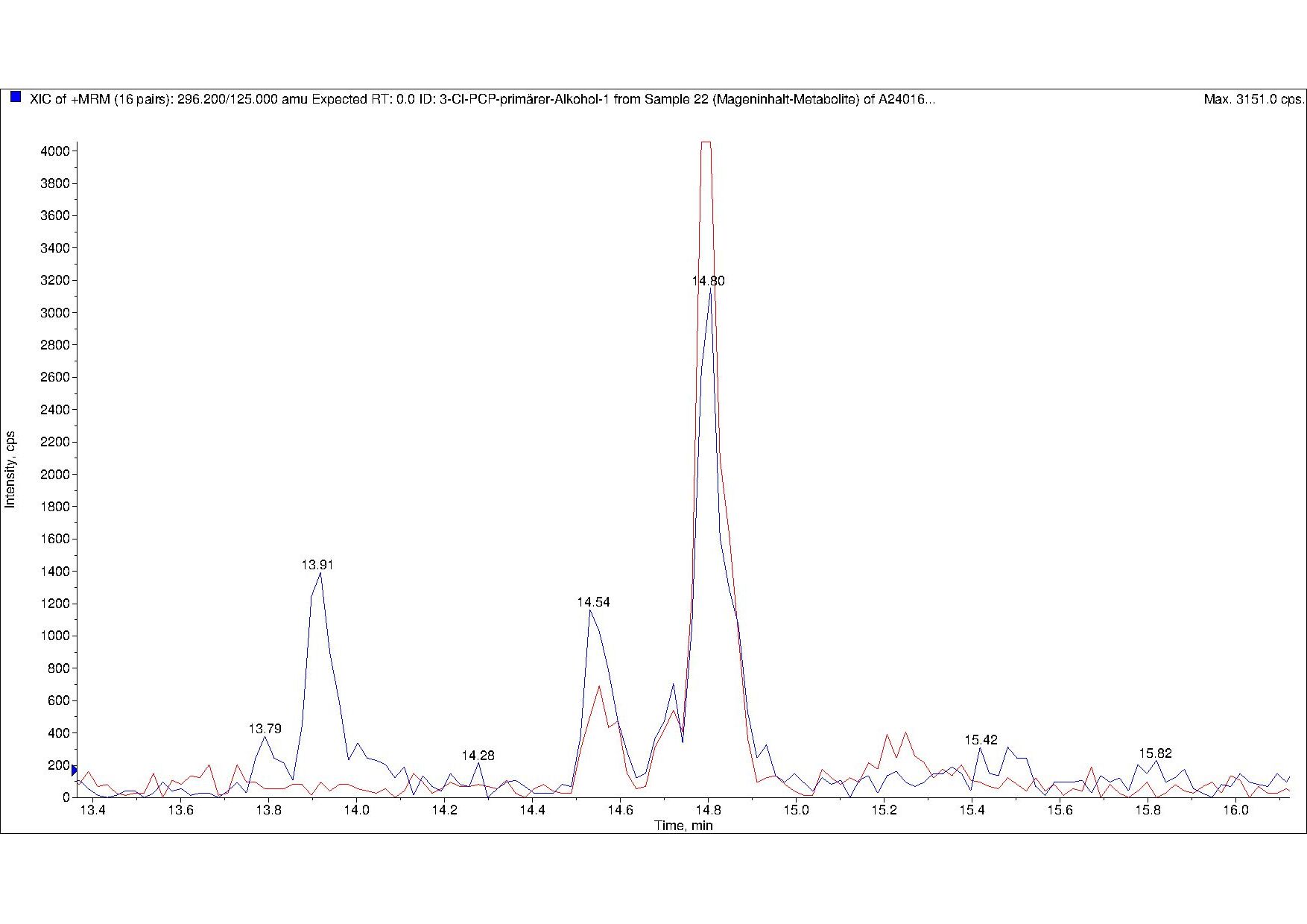  **Figure S32** Chromatogram of the two transitions of the alcohol metabolite M7 in gastric content (enlarged; blue: 296.2 → 125.0, red: 296.2 → 104.1) |
